# *Pi4ka* downregulation triggers Creb3l2-dependent lysosomal dysfunction to promote maladaptive tubular remodeling and immune activation in acute kidney injury

**DOI:** 10.1038/s41419-026-08794-y

**Published:** 2026-04-27

**Authors:** Zhimin Chen, Jingzhi Xie, Chengkun Wu, Keng Ye, Yue Chen, Yankun Song, Huabin Ma, Jianfeng Wu, Li Chen, Yanfang Xu

**Affiliations:** 1 Department of Nephrology, Blood Purification Research Center, the First Affiliated Hospital, Fujian Medical University, Fuzhou, 350005 China; 2 Research Center for Metabolic Chronic Kidney Disease, the First Affiliated Hospital, Fujian Medical University, Fuzhou, 350005 China; 3 Department of Nephrology, National Regional Medical Center, Binhai Campus of the First Affiliated Hospital, Fujian Medical University, Fuzhou, 350212 China; 4 School of Medicine, Nankai University, Tianjin, China; 5 Central Laboratory, the First Affiliated Hospital, Fujian Medical University, Fuzhou, 350005 China; 6 Department of Pathology, the First Affiliated Hospital, Fujian Medical University, Fuzhou, 350005 China; 7 Laboratory Animal Research Center, Xiamen University, Xiameng, 361000 China

**Keywords:** Acute kidney injury, Stress signalling

## Abstract

Acute kidney injury (AKI) is driven by maladaptive tubular responses, yet upstream regulators remain incompletely understood. Here, we identify phosphatidylinositol 4-kinase alpha (*Pi4ka*) as a critical determinant of proximal tubule cell (PTC) homeostasis and injury progression. *PI4KA* expression was reduced in human diseased kidneys and negatively correlated with renal function. Single-cell RNA sequencing in mouse models revealed that *Pi4ka* deficiency preferentially perturbs specific PTC states, including *Slc34a1*^+^*Ccn1*^+^, and *Slc34a1*^+^*Apob*^+^ populations, which diverge along distinct maladaptive trajectories. From these trajectories we derived a 40-gene injury signature enriched for lysosome-associated pathways, and functional assays showed that lysosomal dysfunction is an early event linking *Pi4ka* loss to ER stress, impaired autophagy, and proteostasis disruption. Transcriptional network analysis identified Creb3l2 as a central regulator of lysosomal activation. Notably, Creb3l2 perturbation suppressed stress and cell-death programs while promoting transcriptional programs associated with repair and phospholipid metabolism. Ligand–receptor inference further indicated that *Pi4ka*-deficient PTCs shape a pro-inflammatory immune microenvironment via immunomodulatory gene activation, an effect abolished by *Creb3l2* deletion. Collectively, these findings define a *Pi4ka*–lysosome–*Creb3l2* axis that coordinates tubular injury, maladaptive remodeling, and immune activation, highlighting potential therapeutic targets to limit AKI progression.

## Introduction

Acute kidney injury (AKI) is a common, serious syndrome marked by rapid loss of renal function and substantial morbidity and mortality. Despite improved early recognition and supportive care, AKI remains a major therapeutic challenge, particularly after surgery, during critical illness, and with nephrotoxic exposure [1]. AKI often progresses to chronic kidney disease (CKD), increasing the risk of long-term dysfunction and end-stage kidney disease. Tubular injury, especially in proximal tubular cells (PTCs), is a central feature of AKI. Because PTCs are essential for renal reabsorption, metabolism, and kidney homeostasis [2]. Defining the molecular mechanisms governing PTC injury and repair is critical for developing targeted therapies to limit damage and promote recovery [3].

Phosphatidylinositol 4-kinase alpha (PI4KA) is a key regulator of lipid signaling and membrane trafficking and participates in fundamental cellular processes, including vesicular transport, receptor signaling, and endocytosis [4]. Although PI4KA has been well characterized in multiple tissues [5, 6]. The role of PI4KA in the kidney, particularly in PTCs, remains poorly defined. Emerging evidence has linked lipid signaling pathways to renal physiology and pathophysiology, yet the specific contribution of PI4KA to tubular cell function has not been systematically investigated. Clinical observations have reported reduced PI4KA expression in hydronephrosis and renal dysfunction, suggesting a potential association with impaired kidney function [7, 8]. Together, these observations underscore the need to clarify the role of PI4KA in renal homeostasis and its contribution to kidney injury.

To address this knowledge gap, we employed single-cell RNA sequencing (scRNA-seq) to characterize the cellular and molecular responses to *Pi4ka* deficiency. Compared with bulk RNA sequencing, scRNA-seq provides transcriptomic profiling at single-cell resolution, capturing cell-to-cell heterogeneity and enabling the identification of rare populations and cell type–specific programs. In kidney research, scRNA-seq has proven instrumental in delineating renal cell diversity and dynamic injury responses [9, 10]. Prior studies leveraged this approach to define macrophage-mediated inflammatory pathways and to trace the transition of tubular epithelial cells from injury to repair in AKI models [11]. In CKD, scRNA-seq has uncovered key molecular mediators of fibrosis within fibroblast and myofibroblast subsets [12]. Building on these advances, we applied scRNA-seq to define how *Pi4ka* loss reshapes PTC states and associated injury–repair programs during AKI.

Using scRNA-seq, we delineated how *Pi4ka* deficiency in PTCs shapes injury-associated cellular states and identified downstream pathways engaged by its loss. We further assessed *Pi4ka*-dependent responses across renal cell populations to define their potential contributions to AKI progression. Together, these analyses clarify PI4KA function in kidney pathophysiology and support its evaluation as a therapeutic target in AKI and CKD, while providing a framework for biomarker discovery and strategies to promote renal protection and repair.

## Materials and methods

### Mice

*Pi4ka*^*fl/fl*^ and *Ksp*^*CreERT2*^ mice (GemPharmatech) were crossed to generate *Pi4ka*^*fl/fl*^*Ksp*^*CreERT2*^ mice on a C57BL/6 background, and male mice (10–12 weeks old) housed under SPF conditions were genotyped by tail-snip PCR and used for ischemia/reperfusion injury (IRI) and unilateral ureteral obstruction (UUO) models with blinded outcome assessment. Detailed methods were provided in the Supplementary Information.

### Patients

Control samples were obtained from non-tumorous adjacent renal tissue of patients undergoing nephrectomy for renal carcinoma. All patients were collected in accordance with Good Clinical Practice, the Declaration of Helsinki, and relevant ethical guidelines. Detailed methods were provided in the **Supplementary Information**. Clinical and pathological information of 6 AKI patients and 6 control subjects are provided in Supplementary Table 1.

### Western blot analysis and antibodies

Detailed methods, antibodies, and all original gels are provided in the **Supplementary Material**.

### Cell culture and immunostaining

Primary proximal tubule epithelial cells (PTECs) were freshly isolated as previously described [13, 14]. Detailed methods were provided in Supplementary information.

### Fluorescence imaging of lysosomal acidity, calcium, and ER in cells

LysoTracker™ Green staining (100 nM, 37 °C, 15 min) was used to assess lysosomal acidity, Fluo-4 AM (5 μM, 37 °C, 45 min plus 20–30 min de-esterification) to measure intracellular Ca^2+^ levels, and ER-Tracker Red (1 μM, 37 °C, 15–30 min) to label the endoplasmic reticulum, with fluorescence imaged on a ZEISS LSM800 confocal microscope. Detailed methods were provided in the Supplementary Information.

### Histologic and immunofluorescence analysis of kidney sections

Detailed methods were provided in Supplementary information.

### Preparation of single-cell suspensions and generation of single-cell libraries for sequencing

Mice were euthanized, and kidneys were promptly excised and placed individually into pre-chilled Petri dishes containing cold PBS to preserve tissue integrity. Preparation of single-cell suspensions from kidney tissue, as well as single-cell library construction and sequencing, was performed following established protocols as previously described [9, 10, 15].

### Single cell sequencing data analysis

The detailed methodology, including quality control; gene visualization and cell proportion analysis; unsupervised dimensionality reduction with batch-effect removal and cell type identification; trajectory inference and transcription factor (TF) regulatory network analysis; intercellular ligand–receptor communication analysis; gene enrichment analysis and pathway activity scoring for cell subpopulations; and gene perturbation analysis, is provided in the Supplementary Information.

### Statistical analyses

All results represent data from at least three independent experiments. Statistical analyses were performed using Prism software (GraphPad Software, Inc.). Data are expressed as mean ± SD. Group comparisons were made using an unpaired *t* test, and for multiple comparisons, one-way ANOVA was employed. Statistical significance was defined as *P* < 0.05.

## Results

### *Pi4ka* deficiency in renal tubule cells contributes to kidney injury

**Fig. 1:**
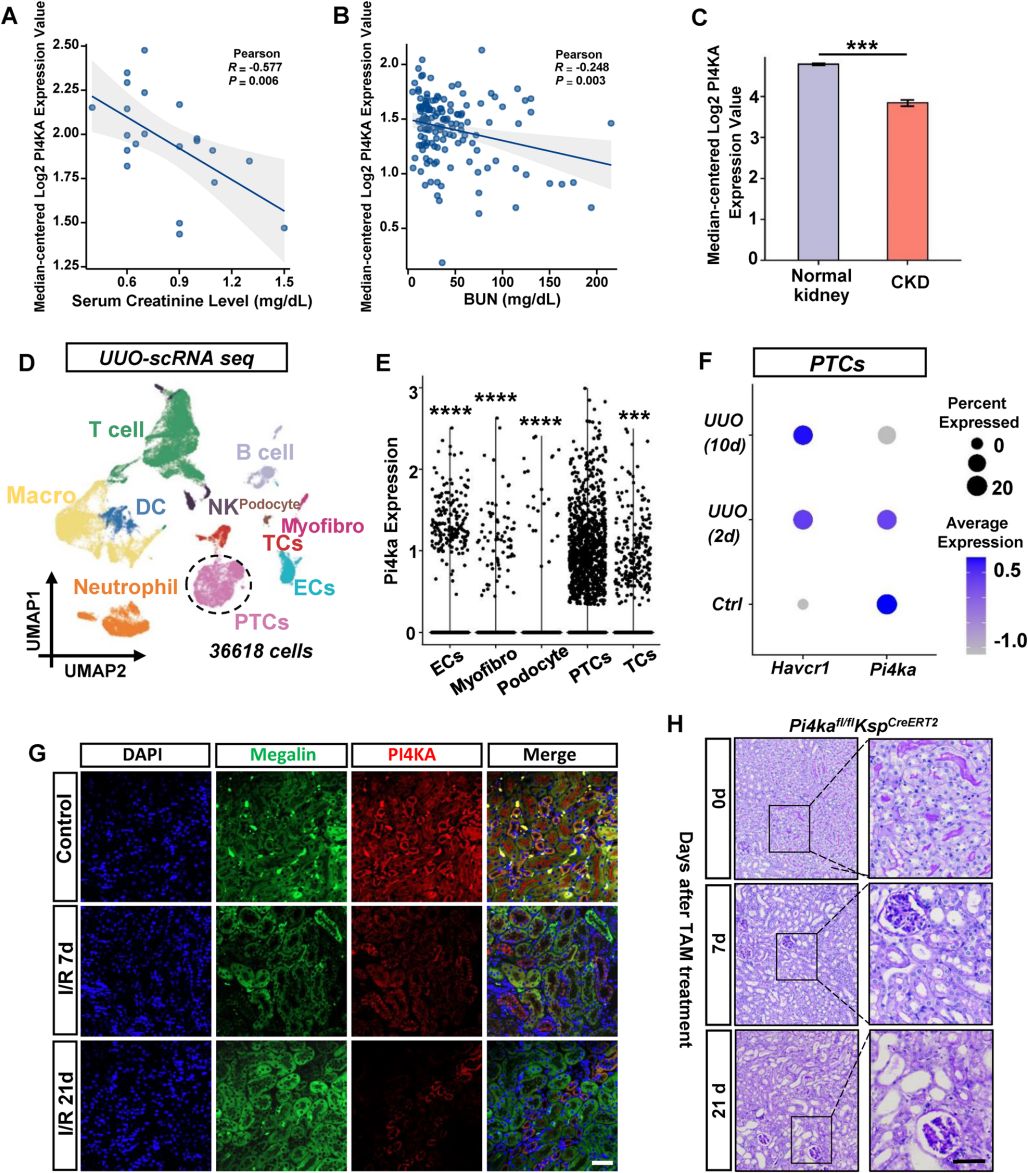
Downregulation of *Pi4ka* in renal tubule cells contributes to the progression of kidney injury. **A**, **B** Analysis of Nephroseq database revealed that *Pi4ka* expression was significantly negatively correlated with serum creatinine (**A**) and blood urea nitrogen (BUN) levels. **C** In an independent dataset, *Pi4ka* expression in the kidneys of CKD mice (*n* = 48) was significantly lower compared with normal kidneys (*n* = 5). Data are presented as mean ± SD. ***P < 0.001. **D** Single-cell transcriptomic data (36,618 cells) from the control and UUO-2d, UUO-10d mouse kidney (*n* = 2 per group) was visualized using UMAP. Macrophages (Macro), dendritic cells (DC), neutrophils, T cells, B cells, NK cells, podocytes, myofibroblasts (Myofibro), endothelial cells (ECs), Podocyte, tubular cells (TCs), and proximal tubular cells (PTCs) were labeled. **E** Abundance of *Pi4ka* expression across different parenchymal cell types. ****P < 0.0001. Data are presented as mean ± SD. ***P < 0.001 vs PTCs. **F** In UUO mice, *Pi4ka* expression in PTCs gradually decreased as the disease progressed (day 2 and day 10), while the expression of the kidney injury marker *Havcr1* significantly increased. **G** Representative immunofluorescence images of kidney sections from I/R mouse models stained for Megalin (green), PI4KA (red), and DAPI (blue) at indicated time points post-injury, *n* = 6 per group. Scale bar = 50 μm. **H** Histological changes in the kidneys of *Pi4ka*^*fl/fl*^*Ksp*^*CreERT2*^ mice at different time points (0, 7, and 21 days) following TAM treatment, with H&E staining, *n* = 6 per group. Scale bar = 50 μm.

We first examined PI4KA expression in kidneys from patients with CKD using Nephroseq database. PI4KA expression was negatively correlated with serum creatinine (Scr) and blood urea nitrogen (BUN) (Fig. 1A–C), indicating an association with impaired renal function and CKD progression. Consistently, immunofluorescence of human AKI biopsies showed reduced PI4KA protein in proximal tubules (Supplementary Fig. 1). To define *Pi4ka* dynamics during injury, we performed scRNA-seq on kidneys from mice subjected to UUO. Uniform manifold approximation and projection (UMAP) analysis of 36,618 cells identified 11 clusters, with *Pi4ka* predominantly expressed in PTCs (Fig. 1D, E). During UUO, *Pi4ka* expression in PTCs decreased early, whereas *Havcr1* was strongly induced [16]. showing an inverse relationship with injury severity (Fig. 1F). Similarly, in wild-type mice subjected to ischemia/reperfusion (I/R), immunofluorescence demonstrated a progressive reduction of Pi4ka (Fig. 1G).

To determine the spatiotemporal contribution of Pi4ka to renal injury, we generated *Pi4ka*^*fl/fl*^*Ksp*^*CreERT2*^ mice and induced proximal tubule–specific *Pi4ka* deletion by TAM administration. Immunoblotting of freshly isolated renal tubules confirmed a time-dependent reduction in PI4KA protein levels after induction (Supplementary Fig. 2A, B). Scr and BUN levels increased over time (Supplementary Fig. 2C, D). Histological examination further showed progressive tubular injury at days 7 and 21, as assessed by hematoxylin and eosin (H&E) staining (Fig. 1H).

Together, these data indicate that PI4KA is downregulated early in kidney injury and that proximal tubule–specific *Pi4ka* deletion is sufficient to trigger renal damage, supporting a causal role for PI4KA deficiency in initiating tubular injury.

### Single-cell transcriptomic profiling reveals progressive injury and compromised repair in PTCs with *Pi4ka* deficiency

**Fig. 2:**
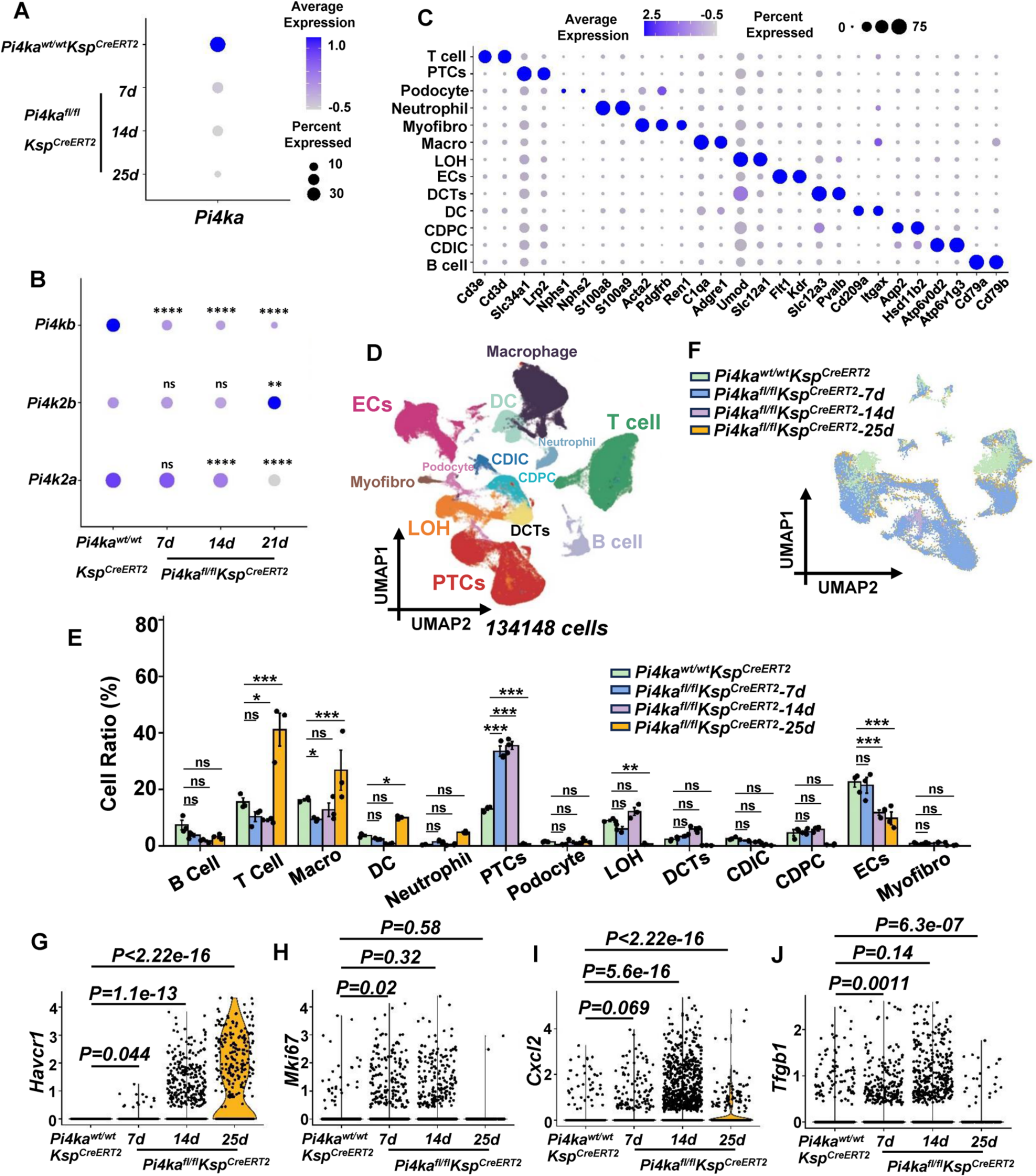
Single-cell transcriptomic profiling of *Pi4ka*^*fl/fl*^*Ksp*^*CreERT2*^ mouse kidney reveals progressive injury and compromised repair in PTCs. **A** Analysis of *Pi4ka* expression in PTCs, showing a gradual decline in *Pi4ka* expression at 7, 14, and 25 days, *n* = 3 per group. **B** Analysis of *Pi4kb*, *Pi4k2a* and *Pi4k2b* expression in *Pi4ka*-deficient PTCs. Data are expressed as mean ± SD. **P < 0.01,****P < 0.0001, *ns* = not significant. **C** Identification of different kidney cell types using marker genes. The dot plot shows the expression of each cell type in the single-cell transcriptome of the *Pi4ka*^*fl/fl*^*Ksp*^*CreERT2*^ mouse kidney, where the color intensity represents the average expression level and the size of the dot reflects the relative proportion of each cell type. Cell subpopulations include T cells, PTCs, podocyte, neutrophil, myofibroblast, macrophage, loop of henle (LOH), endothelial cells (ECs), distal tubule cells (DCTs), dendritic cells (DC), collecting duct principal cell (CDPC), collecting duct intercalated cell (CDIC), and B cells. **D** UMAP visualization of the single-cell transcriptomic analysis identifying 13 different kidney cell types. **E** Changes in the proportion of different cell types in the single-cell transcriptomic map of *Pi4ka*^*wt/wt*^*Ksp*^*CreERT2*^ kidney and *Pi4ka*^*fl/fl*^*Ksp*^*CreERT2*^ mouse kidneys at different time points (7, 14, and 25 days), *n* = 3 per group. Data are expressed as mean ± SD. *P < 0.05, **P < 0.01, ***P < 0.001, *ns* = not significant. **F** After isolating and reclustering PTCs, UMAP dimensionality reduction reveals the distribution of different PTC subgroups. **G** The expression of the injury marker *Havcr1* in PTCs significantly increases over time (7, 14, and 25 days). **H** Expression of the proliferation marker *Mki67* in PTCs at the indicated time points following injury. *Mki67* is elevated at day 7 (*P* < 0.05), shows a similar but non-significant trend at day 14, and declines by day 25. **I** The expression of the maladaptive repair marker *Cxcl2* in PTCs increases at 7 and 14 days but decreases at the final time point (25 days). **J** The expression of the fibrosis marker *Tgfβ* in PTCs significantly increases at 7 and 14 days but declines at the final time point (25 days).

To characterize the transcriptomic landscape after tubule-specific Pi4ka deletion, we performed scRNA-seq on kidneys from *Pi4ka*^*wt/wt*^*Ksp*^*CreERT2*^ and *Pi4ka*^*fl/fl*^*Ksp*^*CreERT2*^ mice at days 7, 14, and 25 after TAM. *Pi4ka* expression in PTCs declined progressively at days 14 and 25, confirming efficient deletion (Fig. 2A). In contrast, expression of other phosphatidylinositol 4-kinases (*Pi4kb*, *Pi4k2a*, and *Pi4k2b*) was not increased, suggesting limited compensatory upregulation following *Pi4ka* loss (Fig. 2B). Given the lack of compensatory induction, *Pi4ka*-deficient PTCs are predicted to have reduced phosphatidylinositol 4-phosphate (PI4P) availability.

Quality control metrics supported dataset integrity (Supplementary Fig. 3A). Using canonical markers, UMAP identified 14 renal populations [9, 10]. (Fig. 2C, D and Supplementary Fig. 4A, B). Cell composition changed over time: PTCs expanded at days 7 and 14 but dropped sharply by day 25, while ECs progressively declined. Immune populations increased, with T cells and macrophages showing the strongest rise at day 25 (Fig. 2E). PTC subclustering revealed time-dependent shifts in subpopulations (Fig. 2F) with marked *Havcr1* induction (Fig. 2G). The proliferation marker *Mki67* was strongly induced at day 7, showed a similar but non-significant trend at day 14, and returned toward baseline by day 25, consistent with an attenuated regenerative response during the chronic phase (Fig. 2H). In parallel, the maladaptive repair–associated chemokine *Cxcl2* and the fibrosis-related factor *Tgfb1* increased at days 7 and 14 but decreased by day 25, suggesting transient inflammatory and profibrotic programs [17]. (Fig. 2I, J).

### *Pi4ka* deficiency characterizes two PTC subpopulations with common injury signatures

**Fig. 3:**
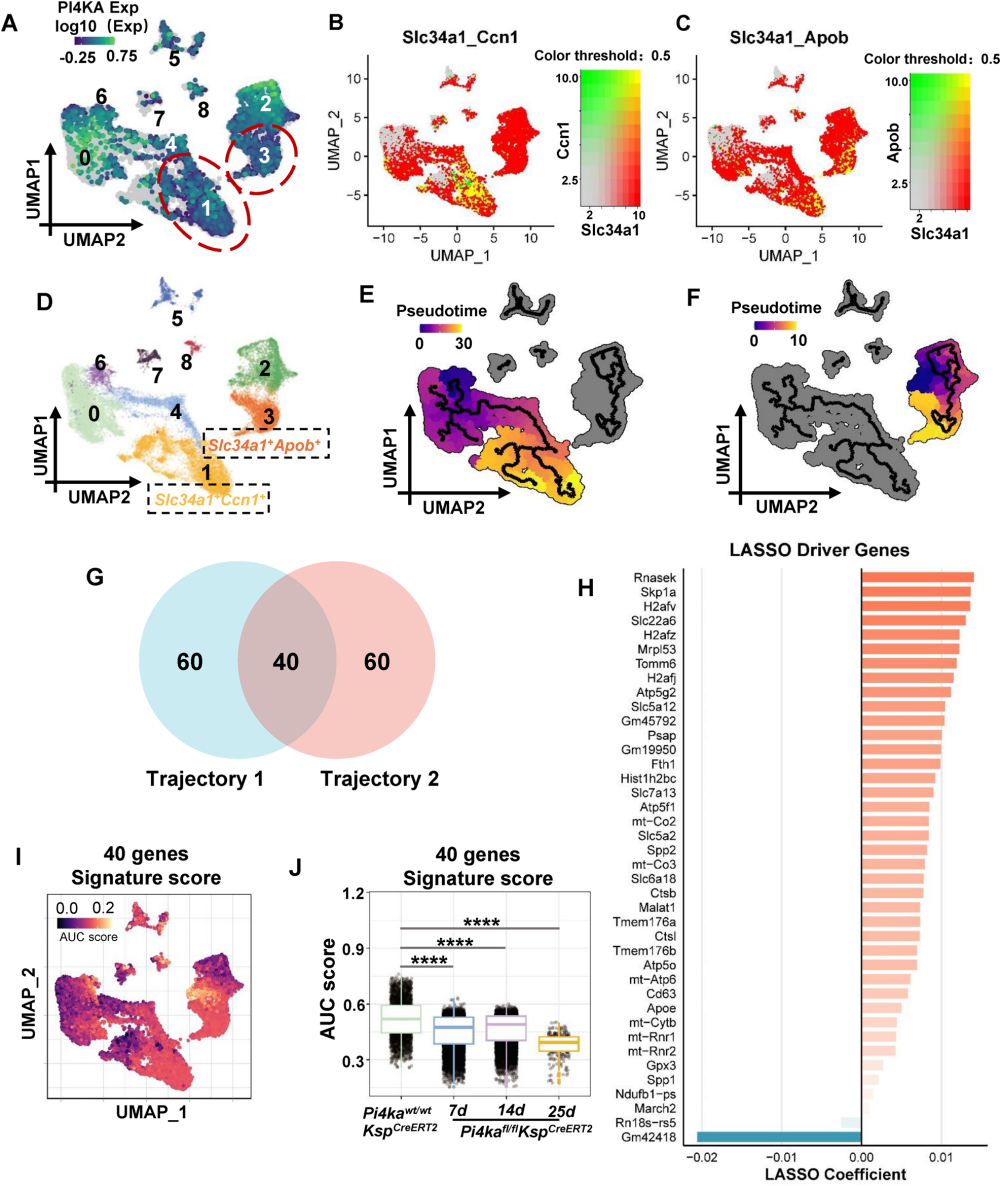
Single-cell transcriptomic dissection of PTC subpopulations reveals *Pi4ka*-dependent differentiation trajectories and injury-associated gene signatures. **A** UMAP projection of proximal tubule cells (PTCs) showing *Pi4ka* expression across subclusters, with pronounced downregulation in clusters 1 and 3. Reclustering and marker-based annotation identified cluster 1 as *Slc34a1*^+^*Ccn1*^+^ cells (**B**) and cluster 3 as *Slc34a1*^+^*Apob*^+^ cells (**C**), respectively. **D** Visualization of annotated PTC subclusters highlights the distinct localization of *Slc34a1*^+^*Ccn1*^+^ and *Slc34a1*^+^*Apob*^+^ populations. Pseudotime trajectory inference demonstrates that *Slc34a1*^+^*Ccn1*^+^ (**E**) and *Slc34a1*^+^*Apob*^+^ (**F**) subpopulations occupy terminal states along divergent differentiation lineages. **G** Venn diagram depicting the overlap of the top 100 trajectory-associated genes from both lineages, with 40 genes shared between trajectories. **H** LASSO regression identifies and ranks the relative contribution of the 40 shared genes. **I** UMAP embedding of a composite gene signature score derived from the 40 shared genes. **J** Quantification of signature scores across *Pi4ka*^*wt/wt*^*Ksp*^*CreERT2*^ and *Pi4ka*^*fl/fl*^*Ksp*^*CreERT2*^ mice at 7, 14, and 25 days post-induction.

To further delineate PTC heterogeneity after *Pi4ka* deletion, we subclustered the scRNA-seq data. *Pi4ka* expression was markedly reduced in specific PTC subclusters, with clusters 1 and 3 showing the strongest downregulation (Supplementary Fig. 5 and Fig. 3A). Marker-based reclustering identified cluster 1 as *Slc34a1*^+^*Ccn1*^+^ PTCs and cluster 3 as *Slc34a1*^+^*Apob*^+^ PTCs, indicating distinct injury-associated states (Fig. 3B–D and Supplementary Table 2). Immunofluorescence in an independent UUO model confirmed APOB and CCN1 expression in SLC34A1-marked proximal tubules during AKI (Supplementary Fig. 6). Pseudotime analysis suggested divergent state transitions: *Slc34a1*^+^*Ccn1*^+^ cells accumulated toward the terminal state of one trajectory, whereas *Slc34a1*^+^*Apob*^+^ cells localized to the endpoint of an alternative branch (Fig. 3E, F). Comparing the top 100 trajectory-associated genes from each branch identified 40 shared genes (Fig. 3G and Supplementary Table 3). LASSO regression prioritized candidate drivers within this set, including *Rnasek*, *H2afv*, and *Slc22a6* (Fig. 3H). A composite signature score based on these 40 genes stratified PTCs by injury-associated state, with lower scores enriched in *Pi4ka*-deficient PTCs (Fig. 3I) and progressively decreasing in *Pi4ka*^*fl/fl*^*Ksp*^*CreERT2*^ mice at days 7, 14, and 25 (Fig. 3J), indicating that this gene set captures the transcriptional burden of *Pi4ka*-related tubular injury.

### Lysosomal pathway activation and corresponding structural impairment are early events in *Pi4ka*-deficient PTC subpopulations

**Fig. 4:**
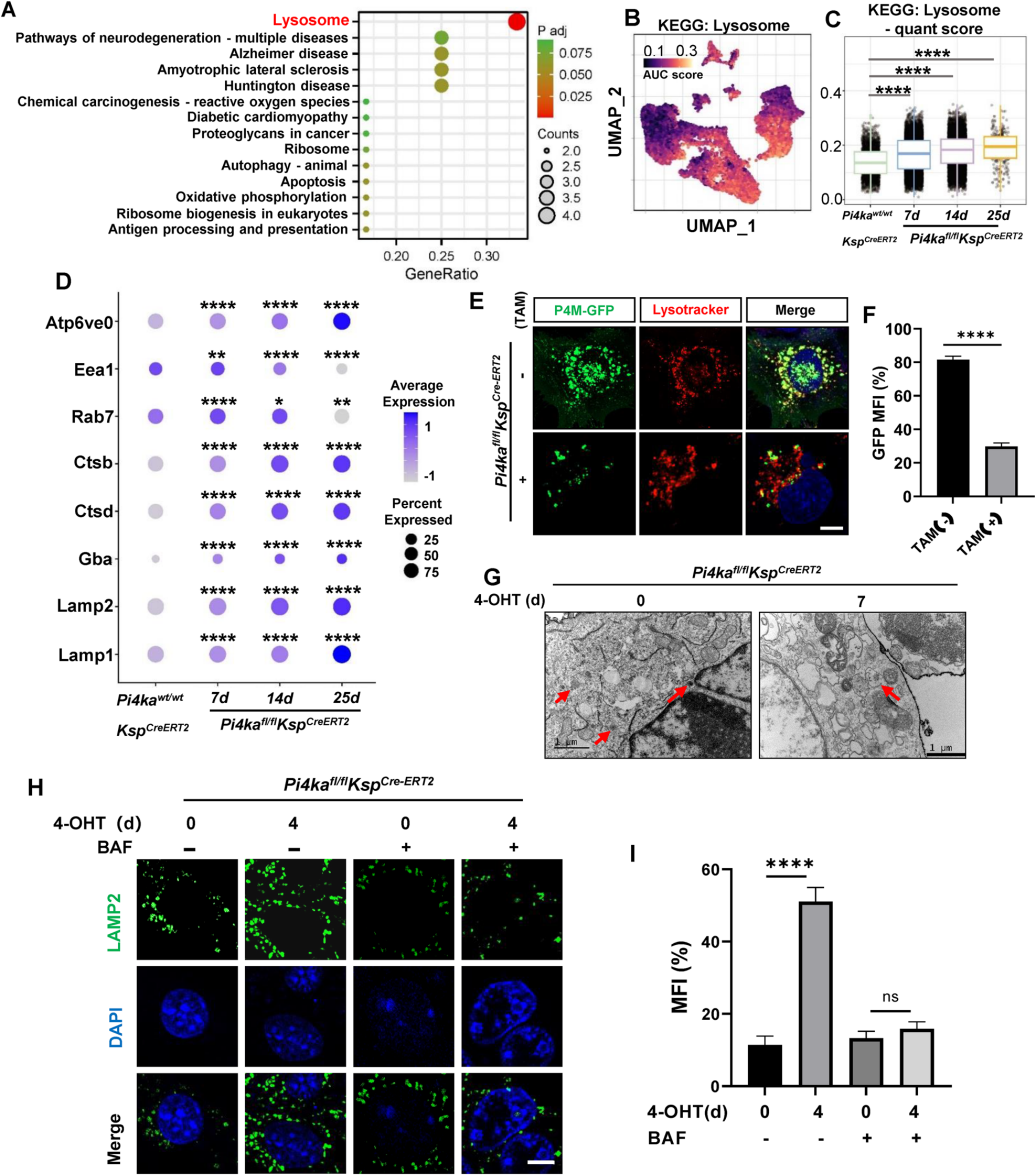
*Pi4ka* deletion induces lysosomal pathway activation and functional disruption in PTCs at early stage. **A** Gene ontology enrichment analysis of the 40 shared trajectory-associated genes revealed significant activation of the lysosome signaling pathway. **B** Gene set scoring demonstrated that lysosome-related transcriptional programs were predominantly enriched in *Slc34a1*^+^*Ccn1*^+^ and *Slc34a1*^+^*Apob*^+^ PTC subpopulations. **C** Quantitative analysis of lysosome signature scores across *Pi4ka*^*wt/wt*^*Ksp*^*CreERT2*^ and *Pi4ka*^*fl/fl*^*Ksp*^*CreERT2*^ mice at 7, 14, and 25 days post-induction. ****P < 0.0001. **D** Expression analysis of representative markers revealed widespread alterations in lysosomal and endosomal compartments, including impaired acidification (*Atp6v0e*), dysregulation of early (*Rab5a*, *Eea1*) and late (*Rab7*) endosomal markers, reduced hydrolytic enzyme expression (*Ctsb*, *Ctsd*), increased lysosomal dysfunction marker (*Gba*), and altered lysosomal membrane proteins (*Lamp1*, *Lamp2*). *P < 0.05, **P < 0.01, ****P < 0.0001. **E** Confocal images of primary tubule cell (*Pi4ka*^*fl/fl*^*Ksp*^*CreERT2*^) before and after 4-OH administration, labeled by P4M-GFP and Lysotracker, *n* = 6 per group. Scale bar = 0.5 μm. **F** Quantification of the P4M-GFP signal intensity in (**E**), showing reduced PI4P reporter signal upon *Pi4ka* deletion, *n* = 6 per group, ****P < 0.0001. **G** Transmission electron microscopy of proximal tubular cells (PTCs) in *Pi4ka*^*fl/fl*^*Ksp*^*CreERT2*^ kidneys at Day 0 (baseline control) and Day 7 (post-induction). Red arrows in the Day 7 indicate lysosomes with abnormal morphology, including swollen and structurally disrupted lysosomes. Scale bar = 1 μm. **H** Representative immunofluorescence images of LAMP2 in primary tubular cells (*Pi4ka*^*fl/fl*^*Ksp*^*CreERT2*^) treated with 4-OH (4 days) with or without bafilomycin A1 (BAF) as indicated. Nuclei were counterstained with DAPI, *n* = 6 per group. Scale bar = 10 μm. **I** Quantification of LAMP2 fluorescence intensity (MFI) corresponding to (**H**) under the indicated 4-OH and BAF treatment conditions, *n* = 6 per group. Data are expressed as mean ± SD. ****P < 0.0001, ns not significant.

To explore subcellular programs associated with *Pi4ka* deficiency in PTCs, we performed enrichment analysis of the 40 trajectory-associated genes identified above. Gene Ontology analysis revealed significant enrichment of lysosome-related pathways (Fig. 4A), suggesting that altered lysosomal programs represent a prominent downstream feature of *Pi4ka* loss. Consistently, gene set scoring showed that lysosome-associated transcriptional signatures were preferentially enriched in *Slc34a1*^+^*Ccn1*^+^ and *Slc34a1*^+^*Apob*^+^ subpopulations (Fig. 4B, C). To further characterize lysosome- and endosome-related changes over time, we examined the expression of canonical markers. *Eea1* and *Rab7* were downregulated, consistent with impaired endosomal maturation and endosome–lysosome trafficking. In contrast, *Atp6v0e* was upregulated, indicating enhanced lysosomal acidification [18, 19]. In parallel, expression of lysosomal hydrolases (*Ctsb*, *Ctsd*, *Gba*) [20, 21]. and lysosomal membrane proteins (*Lamp1*, *Lamp2*) increased [22]. (Fig. 4D). Notably, these transcriptional changes were already evident at the early time point (day 7).

To validate these findings in vitro, we next used primary tubular cells derived from *Pi4ka*^*fl/fl*^*Ksp*^*CreERT2*^ mice. Consistent with our interpretation, *Pi4ka* depletion markedly reduced the association between lysosomes and PI4P [23]. as assessed by P4M-SidM-GFP together with LysoTracker staining (Fig. 4E). Quantification of P4M–GFP signal further confirmed a significant decrease in PI4P abundance upon *Pi4ka* knockout (Fig. 4F). To assess organelle-level consequences, transmission electron microscopy revealed enlarged, structurally abnormal lysosome-like organelles in *Pi4ka*-deficient PTCs compared with controls (Fig. 4G). In parallel, we evaluated lysosomal homeostasis using LAMP2 immunofluorescence under bafilomycin A1 (BAF) challenge. Whereas *Pi4ka*-deficient cells showed robust accumulation of LAMP2-positive structures relative to control cells, yet this induction was markedly diminished upon BAF treatment (Fig. 4H), which was supported by quantitative analysis of LAMP2 signal intensity (Fig. 4I). Together, these assays demonstrate that PI4P depletion following *Pi4ka* loss is accompanied by early lysosomal structural and functional impairment, linking lysosome-pathway activation in injury-associated PTC subpopulations to organelle-level dysfunction.

### Lysosomal dysfunction acts as an upstream driver of ER stress in *Pi4ka*-deficient PTCs

**Fig. 5:**
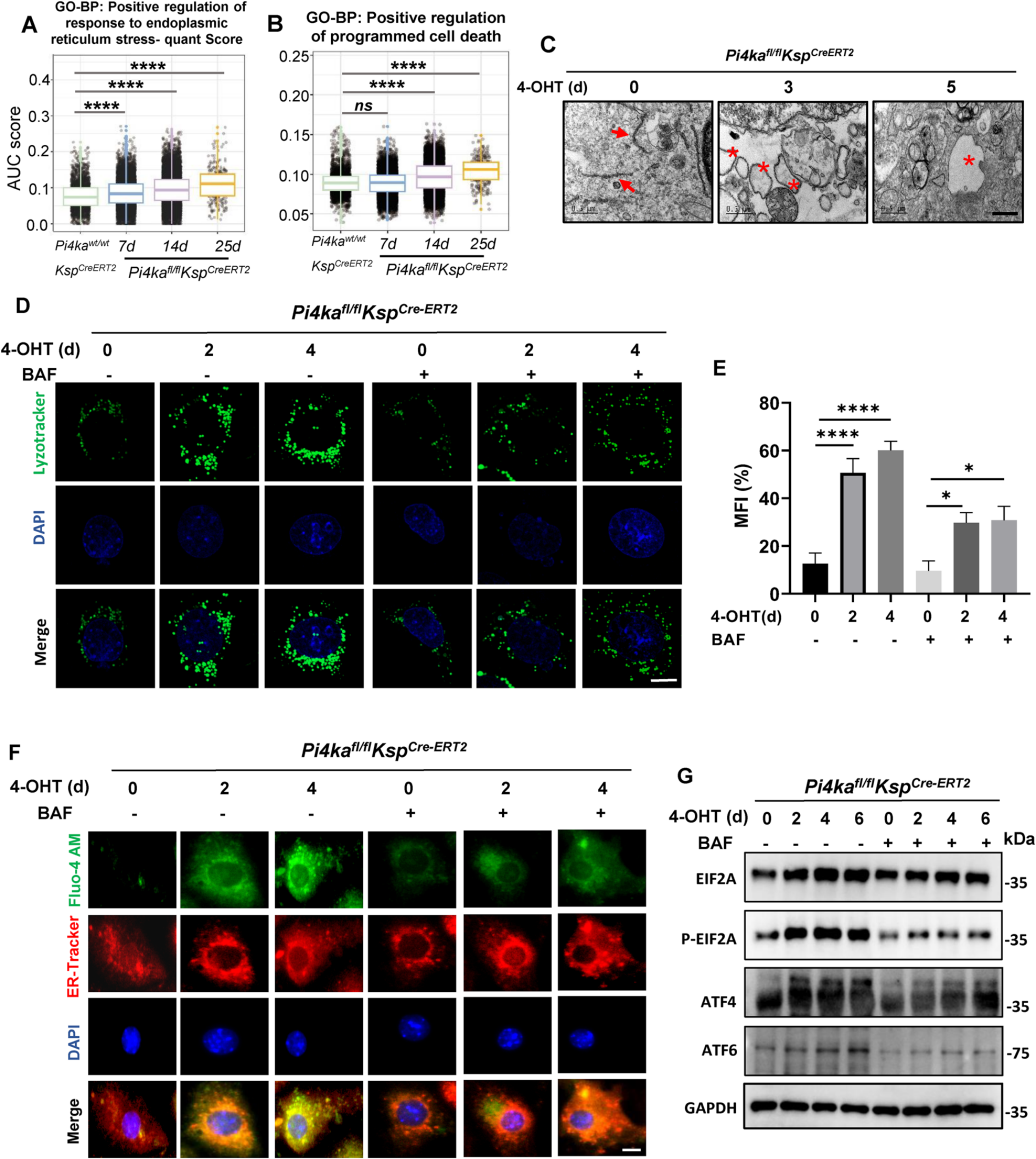
Lysosomal dysfunction acts as an upstream driver of ER stress in *Pi4ka*-deficient PTCs. **A** Gene set enrichment analysis of differentially expressed genes in PTCs at day 7 revealed significant activation of the endoplasmic reticulum (ER) stress response, with quantitative scoring, ****P < 0.0001. **B** At day 14, enrichment analysis demonstrated activation of regulated cell death programs, emerging as the significantly enriched pathway, ****P < 0.0001, *ns* = not significant. **C** Transmission electron microscopy images validated ER stress, showing ultrastructural alterations in cultured PTCs isolated from *Pi4ka*^*fl/fl*^*Ksp*^*CreERT2*^ mice with 4-OHT treatment at day 0, day 3 and day 5. Scale bar = 0.5 μm. **D** Lysotracker staining to assess lysosomal acidity in *Pi4ka*-deficient tubular cells after bafilomycin A1 (BAF) treatment for 0–4 day. *n* = 6 per group. Scale bars = 10 μm. **E** Quantification of LysoTracker fluorescence intensity from (**D**). Mean fluorescence intensity (MFI) was measured in *Pi4ka*-deficient tubular cells treated with 4-OHT for the indicated durations (0, 2, and 4 days) in the absence or presence of BAF, *n* = 6 per group. Data are presented as mean ± SD. *P < 0.05, ****P < 0.0001. **F** Confocal images of Fluo-4 AM (green) and ER-tracker (red). Fluo-4 AM staining showing a suppression of elevated intracellular Ca^2+^ in *Pi4ka*-deficient tubular cells after BAF treatment for 0–4 day, *n* = 6 per group. Scale bars = 10 μm. **G** Immunoblot analysis of ER stress-associated signaling in cultured PTCs isolated from *Pi4ka*^*fl/fl*^*Ksp*^*CreERT2*^ mice treated with 4-OHT for the indicated durations (0, 2, 4, and 6 days) in the absence or presence of BAF, *n* = 4 per group. GAPDH served as a loading control.

Given the marked lysosomal activation in *Pi4ka*-deficient PTCs, we asked whether lysosomal dysfunction is linked to additional pathogenic programs during injury progression. At day 7, enrichment analysis and gene set scoring showed robust induction of ER stress pathways (Fig. 5A and Supplementary Fig. 7). By day 14, cell-death programs—particularly selective autophagy and apoptosis—were prominently enriched, together with renal amyloidosis-like pathology (Fig. 5B and Supplementary Fig. 8, Supplementary Fig. 9A–C). Masson’s trichrome staining revealed progressive interstitial fibrosis (Supplementary Fig. 9D–F), and correlation analyses showed lysosomal activation positively associated with ER stress/UPR, selective autophagy, and fibrosis signatures (Supplementary Fig. 9G).

To validate ER stress in situ, transmission electron microscopy revealed ultrastructural changes at days 7 and 14 (Fig. 5C). Immunoblotting of freshly isolated tubules, consistent with transcriptomic signatures (Supplementary Fig. 7C, 8D and 9F), showed increased ER stress markers (p-eIF2α, ATF4, ATF6, CHOP, PERK) [24]. (Supplementary Fig. 10A) and upregulated autophagy proteins (AMPK, ULK1, LC3B, BECLIN1) [25]. (Supplementary Fig. 10B). Ferroptosis/oxidative stress mediators (GSDMD, NLRP3, NRF2, KEAP1, GPX4, ACSL4) were dysregulated (Supplementary Fig. 10C), together with activation of apoptosis/necrosis pathways (cleaved CASP3, BCL2, BAX, RIPK1/3, MLKL) (Supplementary Fig. 10D) [26].

To define causal hierarchy, in silico perturbation of lysosome-associated pathways reduced ER stress mediators and cell-death genes (Supplementary Fig. 11A, B), whereas perturbation of ER stress regulators suppressed cell-death genes but minimally affected lysosomal structural genes (*Lamp1/2*; *Ctsb/Ctsd/Ctsl*) (Supplementary Fig. 11C, D), supporting lysosomal activation upstream of ER stress and death programs. Next, we sought physiological evidence linking PI4KA depletion to lysosome-associated responses. PI4KA-depleted tubular cells showed increased LysoTracker fluorescence and elevated intracellular Ca^2+^, consistent with altered lysosomal acidification and Ca^2+^ dysregulation. Bafilomycin A1 (BAF), a V-ATPase inhibitor, abolished the LysoTracker signal and attenuated the PI4KA depletion–induced Ca^2+^ increase (Fig. 5D–F). We then asked whether blocking lysosomal acidification/function modulates ER stress. Immunoblotting showed that PI4KA depletion increased p-eIF2α and ATF4/ATF6, and these responses were partially suppressed by BAF (Fig. 5G, Supplementary Fig. 12). Together, these data support a hierarchy in which lysosomal dysfunction acts upstream to promote Ca^2+^ imbalance and ER stress, which then propagates maladaptive cell-death signaling.

### *Creb3l2* is a key transcriptional regulator linking *Pi4ka* deficiency to lysosomal activation in PTCs

**Fig. 6:**
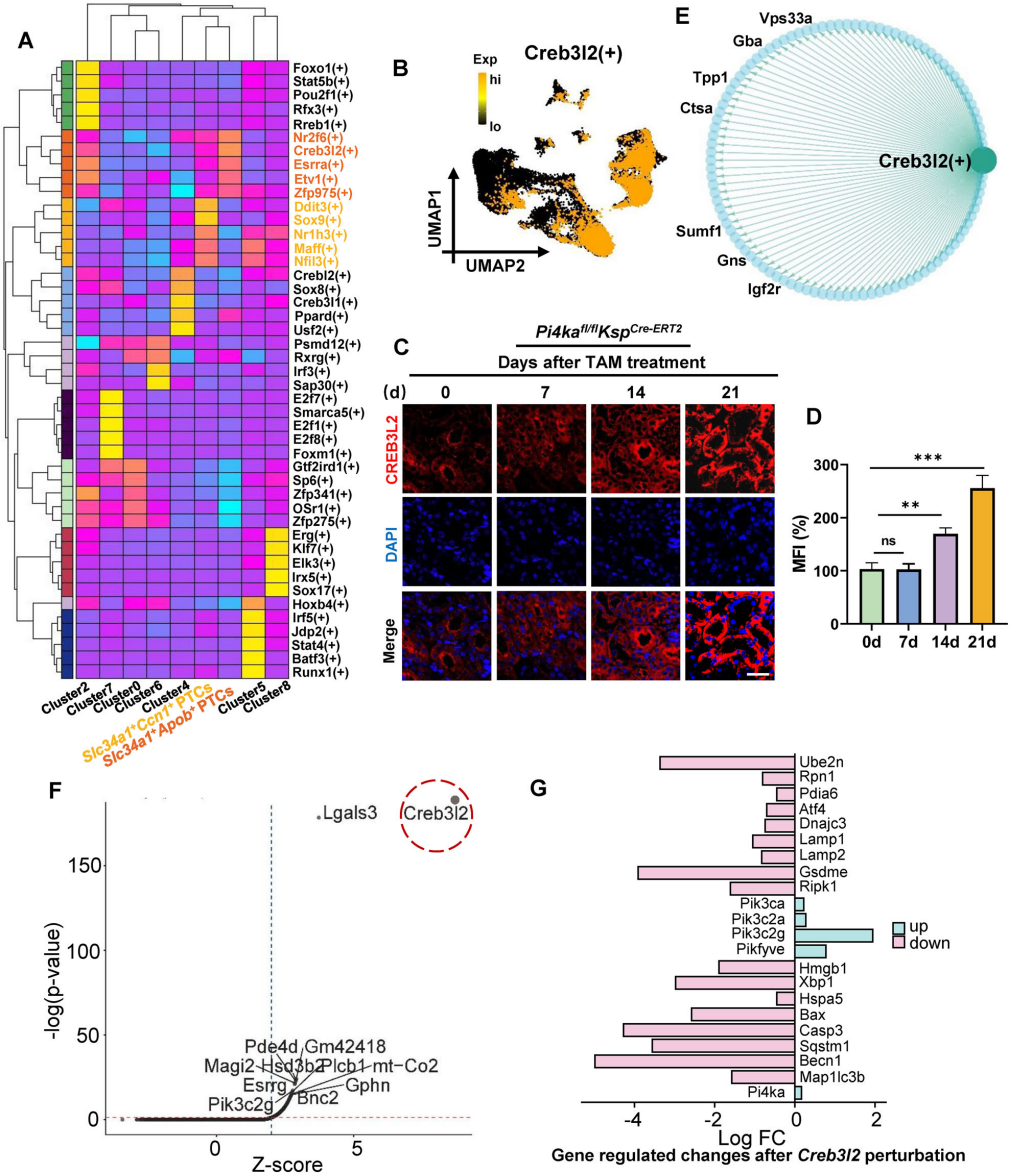
*Creb3l2* functions as a key transcriptional regulator linking *Pi4ka* deficiency to lysosomal activation and downstream pathogenic programs in PTCs. **A** SCENIC regulon analysis across PTC subclusters identified the top five transcription factors (TFs) regulating *Slc34a1*^+^*Ccn1*^+^ (orange) and *Slc34a1*^+^*Apob*^+^ (yellow) populations, highlighting distinct regulatory landscapes in these cell states. **B** UMAP visualization of regulon activity for the top 10 TFs revealed selective activation of *Creb3l2* in PTCs. **C** Representative immunofluorescence images showing CREB3L2 (red) with DAPI nuclear counterstaining (blue) at the indicated time points, *n* = 6 per group. Scale bar = 50 μm. **D** Quantification of CREB3L2 fluorescence intensity (MFI) corresponding to (**C**) at the indicated days after TAM treatment, *n* = 4 per group. Data are expressed as mean ± SD. **P < 0.01, ***P < 0.001, ns = not significant. **E** Transcriptional regulatory network analysis demonstrated that *Creb3l2* directly controls multiple lysosome-associated targets, including *Vps33a*, *Gba*, *Tpp1*, and *Ctsa*, thereby linking *Creb3l2* activity to lysosomal pathway activation. **F** Gene perturbation analysis using the scTenifoldKnk framework predicted that *Creb3l2* knockdown would significantly alter the expression of several regulatory genes, with *Creb3l2* ranking among the most critical upstream factors. **G** Differential expression analysis following *Creb3l2* perturbation showed broad changes across cell death, phospholipid metabolism, ER stress, and lysosome-associated genes.

To identify upstream transcriptional regulators of maladaptive responses in *Pi4ka*-deficient PTCs, we performed SCENIC regulon analysis across PTC subclusters. Regulon activity was preferentially enriched in *Slc34a1*^+^*Ccn1*^+^ and *Slc34a1*^+^*Apob*^+^ populations (Fig. 6A), and enrichment analysis of predicted targets from the top 10 TFs highlighted Creb3l2 as the only candidate linked to lysosome-related pathways (Supplementary Table 4, Supplementary Fig. 13). UMAP visualization confirmed selective enrichment of Creb3l2 regulon activity in both subpopulations (Fig. 6B). Consistently, CREB3L2 protein levels increased during tubular injury and after proximal tubule–specific *Pi4ka* deletion (Fig. 6C, D and Supplementary Fig. 14A, B), with similar induction in IRI and UUO models (Supplementary Fig. 14C–F). Network reconstruction further predicted Creb3l2 regulation of lysosome-associated genes (*Vps33a*, *Gba*, *Tpp1*, *Ctsa*) (Fig. 6E).

**Fig. 7:**
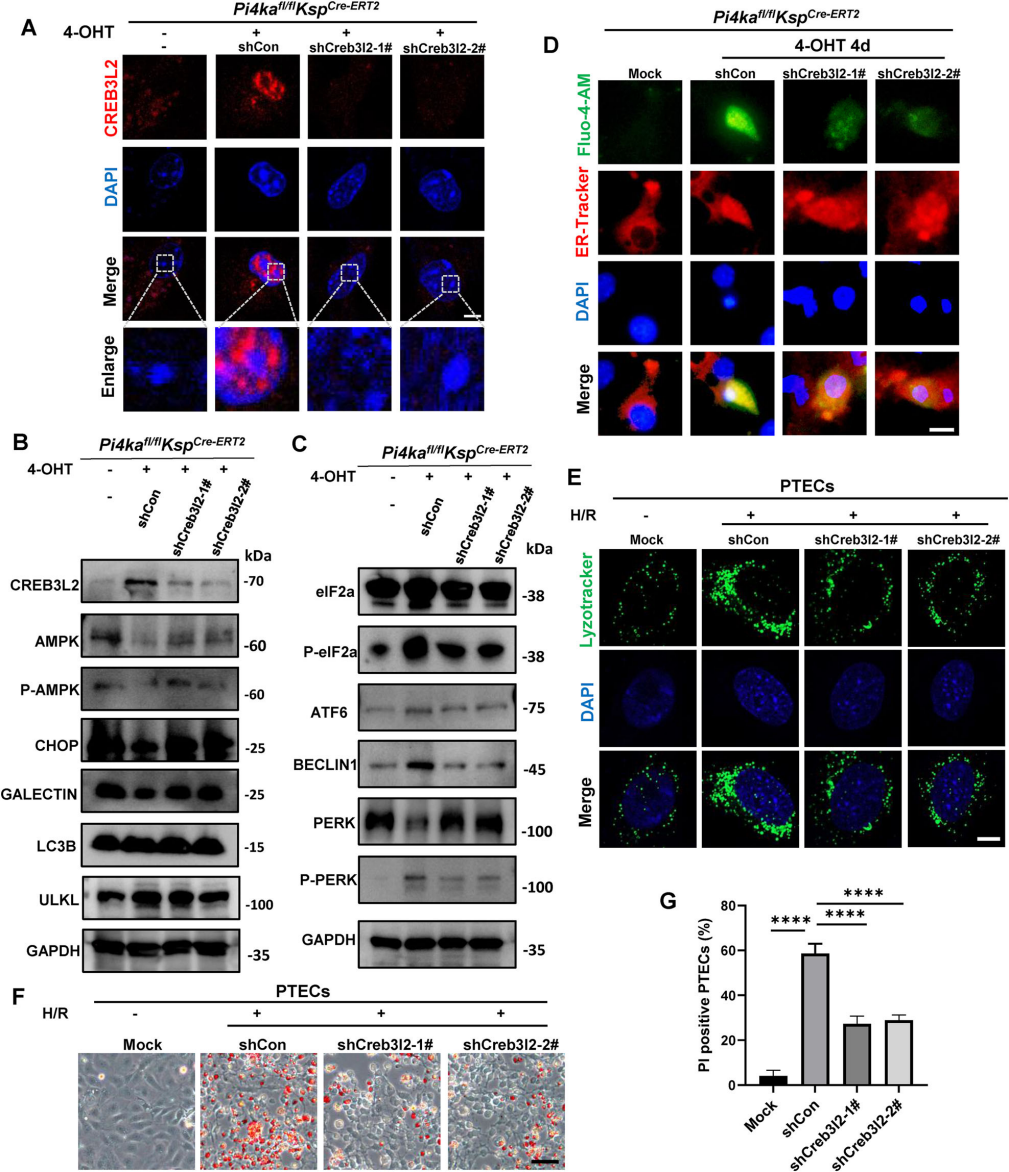
*CREB3L2* knockdown attenuates stress responses downstream of PI4KA loss in tubular cells. **A** Immunofluorescence analysis of CREB3L2 in 4-OHT–treated primary tubular cells (PTECs) from *Pi4ka*^*fl/fl*^*Ksp*^*CreERT2*^ mice transduced with control shRNA (shCon) or two independent Creb3l2 shRNAs. In shCon cells, CREB3L2 shows a nuclear-enriched signal indicative of nuclear translocation, whereas *Creb3l2* knockdown reduces both overall CREB3L2 staining and the nuclear signal, *n* = 4 per group. Scale bars = 10 μm. **B**, **C** Immunoblot analyses of CREB3L2 and indicated stress/autophagy markers in *Pi4ka*-deficient tubular cells transduced with control shRNA or two independent Creb3l2 shRNAs, *n* = 4 per group. GAPDH serves as a loading control. **D** Fluo-4 AM and ER-Tracker staining in *Pi4ka*^*fl/fl*^*Ksp*^*CreERT2*^ tubular cells 4 days after 4-hydroxytamoxifen (4-OHT) treatment with control shRNA or Creb3l2 shRNAs. *n* = 6 per group. Scale bar = 50 μm. **E** LysoTracker staining in primary proximal tubule epithelial cells (PTECs) under basal conditions or after hypoxia/reoxygenation (H/R) with control shRNA or Creb3l2 shRNAs, *n* = 6 per group. Scale bar = 10 μm. **F** Representative images of propidium iodide (PI) staining to visualize cell death under the indicated conditions, *n* = 6 per group. Scale bar = 100 μm. **G** Quantification of PI-positive PTECs, *n* = 6 per group. Data are presented as mean ± SD, ****P < 0.0001.

We next used scTenifoldKnk to simulate *Creb3l2* knockdown, which predicted broad transcriptional effects and positioned *Creb3l2* as an upstream regulatory node (Fig. 6F). Simulated knockdown altered modules including lysosomal genes (*Lamp1/2*), cell-death regulators (*Casp3*, *Bax*, *Ripk1*), phospholipid metabolism genes (*Pik3ca*, *Pikfyve*) [4]. and ER stress markers (*Atf4*, *Hspa5*, *Xbp1*) (Fig. 6G). To test these predictions, we silenced *Creb3l2* with two independent shRNAs in primary PTECs from *Pi4ka*^*fl/fl*^*Ksp*^*CreERT2*^ mice. *Creb3l2* knockdown reduced CREB3L2 expression and diminished its nuclear-enriched signal (Fig. 7A), attenuated injury-associated stress responses (Fig. 7B, C), and partially improved intracellular Ca^2+^ homeostasis (Fig. 7D). In parallel, immunoblotting of freshly isolated tubules detected both full-length CREB3L2 and a cleaved N-terminal form, and the cleaved form increased following TAM-induced Pi4ka deletion (Supplementary Fig. 14A), consistent with CREB3L2 activation. To evaluate generalizability, we also knocked down *Creb3l2* in wild-type primary PTECs subjected to hypoxia/reoxygenation (H/R), which similarly reduced stress responses and improved Ca^2+^ homeostasis (Supplementary Fig. 15). Under H/R, Creb3l2 silencing also decreased lysosome-associated abnormal accumulation (Fig. 7E) and partially alleviated the cell-death (Fig. 7F, G). Collectively, these functional data align with scTenifoldKnk predictions and support Creb3l2 as a regulator linking tubular stress programs to maladaptive injury in *Pi4ka*-deficiency–associated renal injury.

### *Creb3l2* is required for the pro-inflammatory response in *Pi4ka*-deficient PTCs

Within the PTC-to–immune cell signaling axis, Pi4ka-deficient PTCs showed increased intercellular communication at days 14 and 25, with strongest interactions involving macrophages, T cells, and dendritic cells (Supplementary Fig. 16A, B). These PTCs upregulated chemokines (CCL2/3/4/5/7) with predicted engagement of CCR2 and CCR5, and CCL2–CCR2 and CCL5–CCR5 emerged as dominant ligand–receptor pairs. Pi4ka-deficient PTCs also exhibited enhanced pro-inflammatory signaling (APP–TNFRSF21, APP–CD74, RARRES2–CCR2) and enrichment of CD47–SIRPα/SIRPβ1 interactions, consistent with checkpoint-like “don’t-eat-me” signaling and possible chemerin involvement in macrophage activation. Collectively, these data indicate that *Pi4ka*-deficient PTCs actively remodel the immune microenvironment through chemokine-driven recruitment and immune regulatory signaling. PTC–macrophage communication was the most abundant and time-dependent interaction. Consistently, immunofluorescence showed increased macrophage accumulation adjacent to *Megalin*^*+*^ tubules over time, and flow cytometry confirmed increased T cells and dendritic cells after TAM-induced *Pi4ka* deletion (Supplementary Fig. 17).

Dot plots further showed sustained upregulation of ligand-related genes in Pi4ka-deficient PTCs across time points, including *Icam1*, *Cxcl10*, *Ccl4*, *Cd47*, *Cxcl16*, *Rarres2*, *Lipa*, *App*, *Dhcr24*, and *Sirpa* (Supplementary Fig. 16C). To test Creb3l2 dependence, we analyzed *Creb3l2*-deficient PTCs and found reduced expression of many ligand genes induced by *Pi4ka* loss (Supplementary Fig. 16D). In parallel, *Creb3l2* silencing decreased secretion of pro-inflammatory cytokines, including TNFα, IL-1β, and IL-18 (Supplementary Fig. 18). Thus, Creb3l2 supports pro-inflammatory ligand–receptor programs in *Pi4ka*-deficient PTCs, and its suppression mitigates the inflammatory microenvironment.

## Discussion

This study identifies *Pi4ka* as an upstream determinant of PTC fate and reveals how its deficiency promotes AKI progression. Integrating human datasets, mouse models, scRNA-seq, computational perturbation analyses, and functional assays, we define a *Pi4ka*–lysosome–*Creb3l2* axis linking epithelial stress to cell death, immune microenvironment remodeling, and fibrotic progression. Collectively, these findings advance understanding of AKI pathogenesis by positioning *Pi4ka* and lysosome-dependent downstream programs as central drivers of maladaptive tubular responses.

A major strength of this study is showing that *Pi4ka* downregulation is an early, consistent feature of kidney injury. Patient transcriptomes revealed reduced *Pi4ka* in CKD kidneys, correlating with impaired renal function, and models confirmed its progressive decline in ischemic and obstructive injury, supporting the view that tubular dysfunction can initiate maladaptive cascades [27–29]. Unlike HAVCR1 or NGAL, which mark established damage, *Pi4ka* loss appears to precede overt histological changes, consistent with known roles of phosphoinositide kinases in trafficking/signaling required for epithelial integrity [22, 30]. Single-cell analyses indicate selective reprogramming of PTC subpopulations, with *Slc34a1*^+^*Ccn1*^+^ and *Slc34a1*^+^*Apob*^+^ cells most vulnerable and linked to fibrotic and inflammatory programs. CCN1 promotes matrix remodeling/profibrotic signaling [31]. whereas APOB is essential for apoB-lipoprotein (VLDL/LDL) assembly and lipid-particle organization [32]. These shifts provide a cellular framework for heterogeneous tubular responses and identify Pi4ka deficiency as an upstream driver of tubular-state diversity noted in prior scRNA-seq studies [33].

**Fig. 8:**
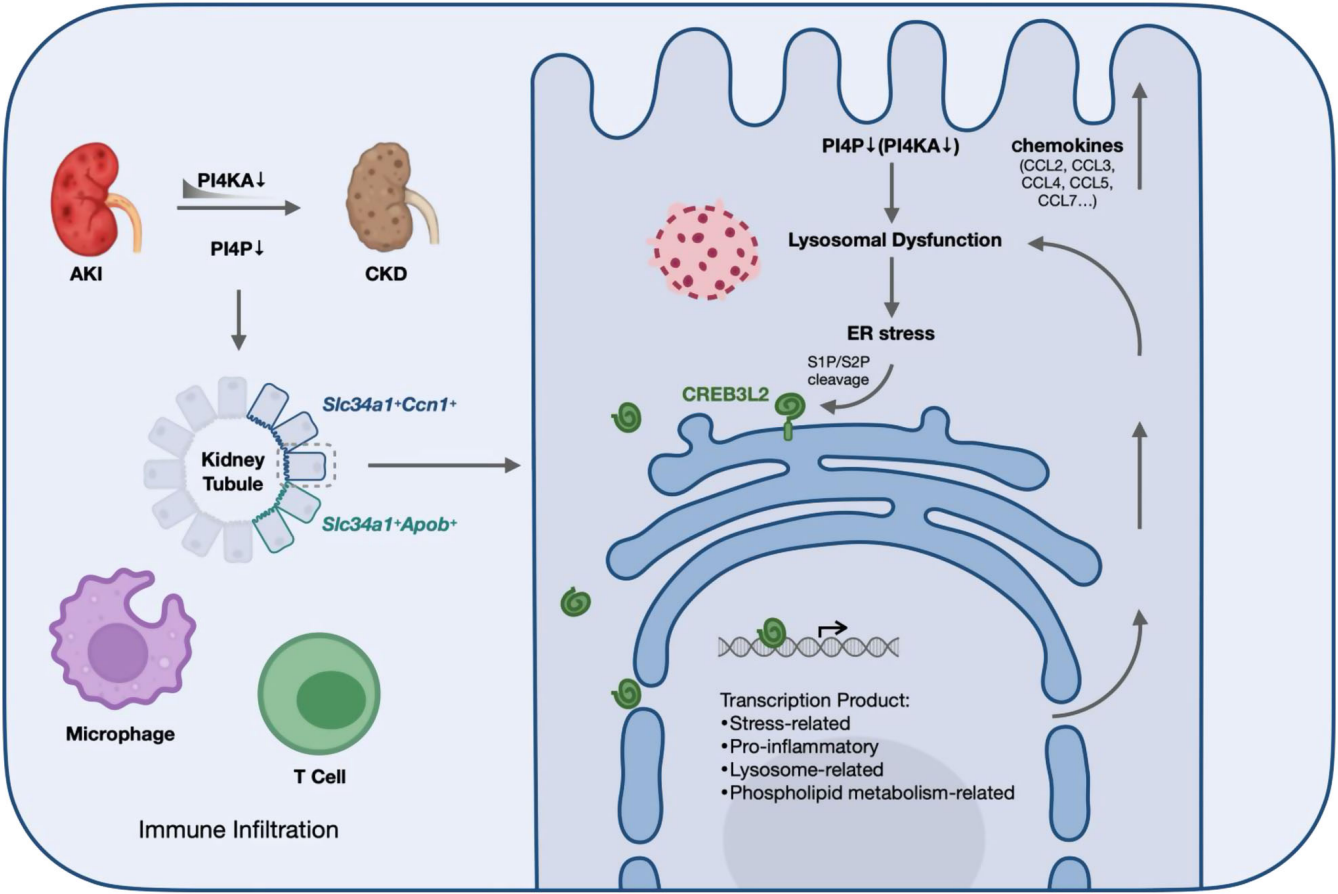
Schematic model illustrating the *Pi4ka*–lysosome–*Creb3l2* axis driving maladaptive tubular remodeling and kidney injury progression. Downregulation of PI4KA in PTCs during AKI leads to the emergence of maladaptive subpopulations, including *Slc34a1*^+^*Ccn1*^+^ and *Slc34a1*^+^*Apob*^+^ cells. *Pi4ka* deficiency precipitates lysosomal dysfunction, which in turn induces ER stress. This stress activates *Creb3l2*, a factor that promotes the expression of genes involved in stress response, pro-inflammation, lysosomal function, and phospholipid metabolism. These combined maladaptive responses facilitate immune cell infiltration and sustain inflammatory signaling, culminating in renal fibrosis and the progression from acute kidney injury (AKI) to chronic kidney disease (CKD).

Our findings identify lysosomal dysfunction as a central node linking *Pi4ka* loss to downstream injury. Lysosomes maintain proteostasis and organelle quality control; their impairment is well recognized in neurodegenerative and metabolic disorders but remains underexplored in AKI [34, 35]. *Pi4ka*-deficient PTCs showed defective acidification, reduced lysosomal hydrolases, and compromised integrity, consistent with impaired clearance and proteostatic failure. This defect was followed by ER stress and autophagy activation, which may be initially compensatory but becomes maladaptive with sustained signaling [36, 37]. Thus, lysosomal dysfunction likely acts as a convergent upstream trigger that enables multiple stress and cell-death programs to co-exist in *Pi4ka*-deficient kidneys [38]. Network analyses highlighted Creb3l2 as a regulator associated with lysosomal activation; although CREB3 factors are linked to ER stress and secretion, their tubular roles are unclear [39, 40]. We propose that Creb3l2 sustains a pro-injury state by regulating lysosome-related genes, and perturbation modeling predicted that *Creb3l2* knockdown suppresses lysosomal and stress signatures; importantly, this was validated by in *vitro* experiments, supporting *Creb3l2* as a maladaptive driver and potential gatekeeper of regeneration [41, 42]. (Fig. 8).

Our study also shows that *Pi4ka*-deficient PTCs can remodel the immune microenvironment. Ligand–receptor analyses revealed increased ICAM1, CXCL10, and CD47, consistent with enhanced macrophage/T-cell recruitment, persistent inflammation, and reduced myeloid clearance, indicating that injured epithelia can transcriptionally program ligand cues that amplify immune infiltration [43, 44]. Suppressing *Creb3l2* attenuated these interaction signatures, supporting epithelial–immune crosstalk as a contributor to injury propagation and paralleling cancer-like immune reshaping by diseased cells [45]. Translationally, this points to potential interventions including preserving *Pi4ka*, targeting the Creb3l2 regulon, using the 40-gene trajectory signature for early risk stratification/monitoring, and modulating axes such as CD47–SIRPα and CCL2–CCR2, suggesting combined epithelial and immune targeting to limit AKI progression. This study has limitations. First, the biochemical mechanisms by which PI4KA maintains lysosomal homeostasis remain unclear; future work should test whether PI4KA-driven changes in phosphoinositide pools directly disrupt endolysosomal dynamics or act via secondary signaling. Second, fibrosis is multicellular: although we focus on epithelial roles, defining reciprocal interactions among PTCs, myofibroblasts, endothelial cells, and infiltrating immune cells will be essential to understand long-term outcomes.

In conclusion, this study identifies a *Pi4ka*–lysosome–*Creb3l2* axis as a central pathway underlying tubular injury and immune remodeling in AKI. By integrating molecular, cellular, and immune analyses, we propose a mechanistic framework that links epithelial stress to inflammation and fibrotic progression. These findings advance current understanding of AKI pathogenesis and suggest new therapeutic opportunities to preserve tubular homeostasis, modulate epithelial–immune crosstalk, and limit the transition from acute kidney injury to chronic kidney disease.

## Supplementary information

Supplementary materials

Supplemental Table2

Supplemental Table3

Supplemental Table4

Original WB images

## Data Availability

The sequencing data has been deposited in the National Center for Biotechnology Sequence Read Archive, https://www.ncbi.nlm.nih.gov/sra (PRJNA1348878). and the GEO database, https://www.ncbi.nlm.nih.gov/geo/ (GSE131685).

## References

[1] Li ZL, Li XY, Zhou Y, Wang B, Lv LL, Liu BC. Renal tubular epithelial cells response to injury in acute kidney injury. EBioMedicine. 2024;107:105294. 39178744 10.1016/j.ebiom.2024.105294 PMC11388183

[2] Ostermann M, Lumlertgul N, Jeong R, See E, Joannidis M, James M. Acute kidney injury. Lancet. 2025;405:241–56. 39826969 10.1016/S0140-6736(24)02385-7

[3] Taguchi K, Sugahara S, Elias BC, Pabla NS, Canaud G, Brooks CR. IL-22 is secreted by proximal tubule cells and regulates DNA damage response and cell death in acute kidney injury. Kidney Int. 2024;105:99–114. 38054920 10.1016/j.kint.2023.09.020 PMC11068062

[4] Burke JE. Structural Basis for Regulation of Phosphoinositide Kinases and Their Involvement in Human Disease. Mol Cell. 2018;71:653–73. 30193094 10.1016/j.molcel.2018.08.005

[5] Barlow-Busch I, Shaw AL, Burke JE. PI4KA and PIKfyve: Essential phosphoinositide signaling enzymes involved in myriad human diseases. Curr Opin Cell Biol. 2023;83:102207. 37453227 10.1016/j.ceb.2023.102207

[6] Salter CG, Cai Y, Lo B, Helman G, Taylor H, McCartney A, et al. Biallelic PI4KA variants cause neurological, intestinal and immunological disease. Brain. 2021;144:3597–3610. 34415310 10.1093/brain/awab313 PMC8719846

[7] Verdura E, Rodríguez-Palmero A, Vélez-Santamaria V, Planas-Serra L, de la Calle I, Raspall-Chaure M, et al. Biallelic PI4KA variants cause a novel neurodevelopmental syndrome with hypomyelinating leukodystrophy. Brain. 2021;144:2659–69. 34415322 10.1093/brain/awab124 PMC8557332

[8] Pagnamenta AT, Howard MF, Wisniewski E, Popitsch N, Knight SJ, Keays DA, et al. Germline recessive mutations in PI4KA are associated with perisylvian polymicrogyria, cerebellar hypoplasia and arthrogryposis. Hum Mol Genet. 2015;24:3732–41. 25855803 10.1093/hmg/ddv117 PMC4459391

[9] Chen Z, Li Y, Yuan Y, Lai K, Ye K, Lin Y, et al. Single-cell sequencing reveals homogeneity and heterogeneity of the cytopathological mechanisms in different etiology-induced AKI. Cell Death Dis. 2023;14:318. 37169762 10.1038/s41419-023-05830-z PMC10175265

[10] Chen Z, Lin G, Ye K, Wang J, Tang M, Lai K, et al. Single-cell analysis of diquat-induced oxidative stress and its impact on organ-specific toxicity. Ecotoxicol Environ Saf. 2025;297:118246. 40327929 10.1016/j.ecoenv.2025.118246

[11] Yao W, Chen Y, Li Z, Ji J, You A, Jin S, et al. Single Cell RNA Sequencing Identifies a Unique Inflammatory Macrophage Subset as a Druggable Target for Alleviating Acute Kidney Injury. Adv Sci (Weinh). 2022;9:e2103675. 35112806 10.1002/advs.202103675 PMC9036000

[12] Zhang YL, Tang TT, Wang B, Wen Y, Feng Y, Yin Q, et al. Identification of a Novel ECM Remodeling Macrophage Subset in AKI to CKD Transition by Integrative Spatial and Single-Cell Analysis. Adv Sci (Weinh). 2024;11:e2309752. 39119903 10.1002/advs.202309752 PMC11481374

[13] Lai K, Chen Z, Lin S, Ye K, Yuan Y, Li G, et al. The IDH1-R132H mutation aggravates cisplatin-induced acute kidney injury by promoting ferroptosis through disrupting NDUFA1 and FSP1 interaction. Cell Death Differ. 2025;32:242–55. 10.1038/s41418-024-01381-8 PMC11802792 39306640

[14] Chen C, Xie J, Chen Z, Ye K, Wu C, Dai X, et al. Role of Z-DNA binding protein 1 sensing mitochondrial Z-DNA and triggering necroptosis in oxalate-induced acute kidney injury. J Am Soc Nephrol. 2025;36:361–77. 39374087 10.1681/ASN.0000000516 PMC11888962

[15] Wang Y, Li Y, Chen Z, Yuan Y, Su Q, Ye K, et al. GSDMD-dependent neutrophil extracellular traps promote macrophage-to-myofibroblast transition and renal fibrosis in obstructive nephropathy. Cell Death Dis. 2022;13:693. 35941120 10.1038/s41419-022-05138-4 PMC9360039

[16] Mori Y, Ajay AK, Chang JH, Mou S, Zhao H, Kishi S, et al. KIM-1 mediates fatty acid uptake by renal tubular cells to promote progressive diabetic kidney disease. Cell Metab. 2021;33:1042–61.e1047. 33951465 10.1016/j.cmet.2021.04.004 PMC8132466

[17] Park J, Shrestha R, Qiu C, Kondo A, Huang S, Werth M, et al. Single-cell transcriptomics of the mouse kidney reveals potential cellular targets of kidney disease. Science. 2018;360:758–63. 29622724 10.1126/science.aar2131 PMC6188645

[18] Mauvezin C, Neufeld TP. Bafilomycin A1 disrupts autophagic flux by inhibiting both V-ATPase-dependent acidification and Ca-P60A/SERCA-dependent autophagosome-lysosome fusion. Autophagy. 2015;11:1437–38. 26156798 10.1080/15548627.2015.1066957 PMC4590655

[19] Abu-Remaileh M, Wyant GA, Kim C, Laqtom NN, Abbasi M, Chan SH, et al. Lysosomal metabolomics reveals V-ATPase- and mTOR-dependent regulation of amino acid efflux from lysosomes. Science. 2017;358:807–13. 29074583 10.1126/science.aan6298 PMC5704967

[20] Chae CW, Yoon JH, Lim JR, Park JY, Cho JH, Jung YH, et al. TRIM16-mediated lysophagy suppresses high-glucose-accumulated neuronal Aβ. Autophagy. 2023;19:2752–68. 37357416 10.1080/15548627.2023.2229659 PMC10472864

[21] Wang K, Fu S, Dong L, Zhang D, Wang M, Wu X, et al. Periplocin suppresses the growth of colorectal cancer cells by triggering LGALS3 (galectin 3)-mediated lysophagy. Autophagy. 2023;19:3132–50. 37471054 10.1080/15548627.2023.2239042 PMC10621285

[22] Choi J, Jang H, Xuan Z, Park D. Emerging roles of ATG9/ATG9A in autophagy: implications for cell and neurobiology. Autophagy. 2024;20:2373–87. 39099167 10.1080/15548627.2024.2384349 PMC11572220

[23] Tan JX, Finkel T. A phosphoinositide signalling pathway mediates rapid lysosomal repair. Nature. 2022;609:815–21. 36071159 10.1038/s41586-022-05164-4 PMC9450835

[24] Chen X, Shi C, He M, Xiong S, Xia X. Endoplasmic reticulum stress: molecular mechanism and therapeutic targets. Signal Transduct Target Ther. 2023;8:352. 37709773 10.1038/s41392-023-01570-w PMC10502142

[25] Glick D, Barth S, Macleod KF. Autophagy: cellular and molecular mechanisms. J Pathol. 2010;221:3–12. 20225336 10.1002/path.2697 PMC2990190

[26] Newton K, Strasser A, Kayagaki N, Dixit VM. Cell death. Cell. 2024;187:235–56. 38242081 10.1016/j.cell.2023.11.044

[27] Gerhardt LMS, Koppitch K, van Gestel J, Guo J, Cho S, Wu H, et al. Lineage Tracing and Single-Nucleus Multiomics Reveal Novel Features of Adaptive and Maladaptive Repair after Acute Kidney Injury. J Am Soc Nephrol. 2023;34:554–71. 36735940 10.1681/ASN.0000000000000057 PMC10103206

[28] Gerhardt LMS, Liu J, Koppitch K, Cippà PE, McMahon AP. Single-nuclear transcriptomics reveals diversity of proximal tubule cell states in a dynamic response to acute kidney injury. Proc Natl Acad Sci USA. 2021;118:e2026684118. 10.1073/pnas.2026684118 PMC8271768 34183416

[29] Zou Z, Ye K, Chen F, Lin K, Song Y, Li G, et al. ZBP1 promotes RIPK1-dependent apoptosis in aristolochic acid nephropathy. Zool Res. 2026;47:577–92. 10.24272/j.issn.2095-8137.2025.358 PMC13430209 42011760

[30] Backer JM. Phosphoinositide 3-kinases and the regulation of vesicular trafficking. Mol Cell Biol Res Commun. 2000;3:193–204. 10891392 10.1006/mcbr.2000.0202

[31] Fischer AG, Elliott EM, Brittian KR, Garrett L, Sadri G, Aebersold J, et al. Matricellular protein CCN1 promotes collagen alignment and scar integrity after myocardial infarction. Matrix Biol. 2024;133:14–32. 39098433 10.1016/j.matbio.2024.08.001 PMC11476287

[32] Fogelstrand P, Borén J. Retention of atherogenic lipoproteins in the artery wall and its role in atherogenesis. Nutr Metab Cardiovasc Dis. 2012;22:1–7. 22176921 10.1016/j.numecd.2011.09.007

[33] Rudman-Melnick V, Adam M, Potter A, Chokshi SM, Ma Q, Drake KA, et al. Single-cell profiling of AKI in a murine model reveals novel transcriptional signatures, profibrotic phenotype, and epithelial-to-stromal crosstalk. J Am Soc Nephrol. 2020;31:2793–2814. 33115917 10.1681/ASN.2020010052 PMC7790221

[34] Deus CM, Yambire KF, Oliveira PJ, Raimundo N. Mitochondria-lysosome crosstalk: from physiology to neurodegeneration. Trends Mol Med. 2020;26:71–88. 31791731 10.1016/j.molmed.2019.10.009

[35] Udayar V, Chen Y, Sidransky E, Jagasia R. Lysosomal dysfunction in neurodegeneration: emerging concepts and methods. Trends Neurosci. 2022;45:184–99. 35034773 10.1016/j.tins.2021.12.004 PMC8854344

[36] Minami S, Yamamoto T, Yamamoto-Imoto H, Isaka Y, Hamasaki M. Autophagy and kidney aging. Prog Biophys Mol Biol. 2023;179:10–15. 36849016 10.1016/j.pbiomolbio.2023.02.005

[37] Livingston MJ, Shu S, Fan Y, Li Z, Jiao Q, Yin XM, et al. Tubular cells produce FGF2 via autophagy after acute kidney injury leading to fibroblast activation and renal fibrosis. Autophagy. 2023;19:256–77. 35491858 10.1080/15548627.2022.2072054 PMC9809951

[38] Ye K, Chen Z, Xu Y. The double-edged functions of necroptosis. Cell Death Dis. 2023;14:163. 36849530 10.1038/s41419-023-05691-6 PMC9969390

[39] Kondo Y, Fu J, Wang H, Hoover C, McDaniel JM, Steet R, et al. Site-1 protease deficiency causes human skeletal dysplasia due to defective inter-organelle protein trafficking. JCI Insight. 2018;3:e121596. 10.1172/jci.insight.121596 PMC6124414 30046013

[40] Smith BS, Hewitt T, Bakovic M, Lu R. ER stress-associated transcription factor CREB3 is essential for normal Ca(2+), ATP, and ROS homeostasis. Mitochondrion. 2023;69:10–17. 36627030 10.1016/j.mito.2023.01.001

[41] Yoo EJ, Oh KH, Piao H, Kang HJ, Jeong GW, Park H, et al. Macrophage transcription factor TonEBP promotes systemic lupus erythematosus and kidney injury via damage-induced signaling pathways. Kidney Int. 2023;104:163–80. 37088425 10.1016/j.kint.2023.03.030

[42] Huang J, Meng P, Liang Y, Li X, Zhou S, Li J, et al. Tubular CD44 plays a key role in aggravating AKI through NF-κB p65-mediated mitochondrial dysfunction. Cell Death Dis. 2025;16:119. 39979265 10.1038/s41419-025-07438-x PMC11842857

[43] Barbir EB, Kitchlu A, Herrmann SM. Immune checkpoint inhibitor-associated nephritis-treatment standard. Nephrol Dial Transplant. 2024;39:1785–98. 39138117 10.1093/ndt/gfae184

[44] Lee K, Jang HR, Rabb H. Lymphocytes and innate immune cells in acute kidney injury and repair. Nat Rev Nephrol. 2024;20:789–805. 39095505 10.1038/s41581-024-00875-5

[45] Yang Z, Zhou B, Guo W, Peng Y, Tian H, Xu J, et al. Genomic characteristics and immune landscape of super multiple primary lung cancer. EBioMedicine. 2024;101:105019. 38364701 10.1016/j.ebiom.2024.105019 PMC10878856

