# Supplementary material for: Pi4ka downregulation triggers Creb3l2-dependent lysosomal dysfunction to promote maladaptive tubular remodeling and immune activation in acute kidney injury
Source: Cell Death Dis. 2026 Apr 27;17(1):557. doi: 10.1038/s41419-026-08794-y (PMC13250169; doi:10.1038/s41419-026-08794-y)
Supplement: Supplementary file 1 — Supplementary materials [file 41419_2026_8794_MOESM1_ESM.pdf]

Supplementary Materials for

Pi4ka downregulation triggers Creb3l2-dependent lysosomal dysfunction to promote maladaptive tubular remodeling and immune activation in acute kidney injury

Zhimin Chen[1,2,3,8], Jingzhi Xie[1,2,3,8], Chengkun Wu[4,8], Keng Ye[1,2,3], Yue Chen[5], Yankun Song[6], Huabin Ma[5], Jianfeng Wu[7], Li Chen[1,2,5]✉, Yanfang Xu[1,2,3,5]✉

[1]Department of Nephrology, Blood Purification Research Center, the First Affiliated Hospital, Fujian Medical University, Fuzhou 350005, China

[2]Research Center for Metabolic Chronic Kidney Disease, the First Affiliated Hospital, Fujian Medical University, Fuzhou 350005, China

[3]Department of Nephrology, National Regional Medical Center, Binhai Campus of the First Affiliated Hospital, Fujian Medical University, Fuzhou 350212, China.

[4]School of Medicine, Nankai University, Tianjin, China.

[5]Central Laboratory, the First Affiliated Hospital, Fujian Medical University, Fuzhou 350005, China

[6]Department of Pathology, the First Affiliated Hospital, Fujian Medical University, Fuzhou 350005, China

[7]Laboratory Animal Research Center, Xiamen University, Xiameng 361000, China

[8]Zhimin Chen, Jingzhi Xie and Chengkun Wu contributed equally to this work.

***Correspondence**

Chenli_16@163.com (L.C.)

xuyanfang99@hotmail.com (Y.X.)

This file includes:

MATERIALS AND METHODS

Supplemental Table 1

Supplementary Fig. 1-18

Caption of Supplemental Table 2: Differentially expressed genes for each cluster of reclustered PTCs in *Pi4ka*$^{fl/fl}$*Ksp*^{CreERT2} mice.

Caption of Supplemental Table 3: The differentiation-related genes of the two trajectories (Trajectory 1 and Trajectory 2) of PTCs injury differentiation.

Caption of Supplemental Table 4: Key transcription factors and their regulated target genes.

MATERIALS AND METHODS

Mice

Both *Pi4ka*$^{fl/fl}$ and *Ksp*^{CreERT2} mice were obtained from GemPharmatech Co., Ltd. These Ksp^{CreERT2} mice were genetically engineered to delete floxed genes specifically in tubular epithelial cells upon tamoxifen (TAM) induction, through activation of the TAM-responsive ERT fusion protein. To generate *Pi4ka*$^{fl/fl}$*Ksp*^{CreERT2} mice, intercrossing was performed between *Ksp*^{CreERT2} and *Pi4ka*$^{fl/fl}$ mice on a C57BL/6 background. All mice were housed in a specific pathogen-free (SPF) facility with a 12-hour light/dark cycle. Male mice (10-12 weeks old, weighing 24-28g) were used in this study. Mice genotyping was performed via tail-snip PCR amplification. Investigators were blinded to the experimental groups during outcome assessments, including histological analysis, cell morphology, proteomics, and Western blot. The ischemia/reperfusion injury (IRI) and unilateral ureteral obstruction (UUO) mouse models were established as previously described [1]. All animal procedures were conducted in strict accordance with ethical guidelines and were approved by the Laboratory Animal Management and Ethics Committee of Fujian Medical University (Approval No. IACUC FJMU 2024-Y-0875). The experiments adhered to the "China Guide for the Protection and Use of Laboratory Animals."

Patients

This study was approved by the Ethics Committee of the First Affiliated Hospital of Fujian Medical University (approval number [2023]247), and written informed consent was obtained from all participants. All procedures were conducted in accordance with the principles of Good Clinical Practice, the Declaration of Helsinki, and relevant ethical guidelines for biomedical research involving human subjects. Control samples were obtained from non-tumorous adjacent renal tissue of patients undergoing nephrectomy for renal carcinoma. Clinical and pathological information of 6 AKI patients and 6 control subjects are provided in **Supplementary Table S1**. Due to the abrupt onset of AKI, baseline serum creatinine values were unavailable for these patients; therefore, AKI staging was retrospectively determined according to the 2012 KDIGO Clinical Practice Guidelines, based on serum creatinine levels obtained during

a 6-months follow-up.

Western blot analysis and antibodies

For protein extraction from kidney tissue, approximately 50 mg of tissue was homogenized in 400–500μL of radioimmunoprecipitation assay buffer supplemented with protease and phosphatase inhibitor cocktails. The total protein concentration was quantified using the Bradford assay. Subsequently, sodium dodecyl sulfate (SDS) was added to the lysates. For in vitro studies, cultured cells were directly collected and lysed using 1.2× SDS sample buffer. All protein samples were resolved by SDS–polyacrylamide gel electrophoresis (SDS–PAGE) and transferred onto polyvinylidene difluoride membranes (EMD Millipore) for immunoblotting. Membranes were blocked in 5% bovine serum albumin for 1 hour at room temperature and then incubated overnight at 4°C with specific primary antibodies. Following three washes with Tris-buffered saline containing 0.1% Tween-20, membranes were incubated with horseradish peroxidase-conjugated secondary antibodies—either Goat Anti-Mouse IgG (H+L) (ABclonal, AS003) or Goat Anti-Rabbit IgG (H+L) (ABclonal, AS014)—for 1 hour at room temperature. Protein bands were detected using enhanced chemiluminescence reagents and visualized with the ChemiDoc Imaging System (BIO-RAD).

The following antibodies were used in this study: anti-RIPK1 (Cell Signaling Technology, 3493T), anti-Phospho-RIPK1 (Ser166) (Cell Signaling Technology, 31122S), anti-Caspase3 (Cell Signaling Technology, 9662), anti-Caspase8 (Cell Signaling Technology, 4790S), anti-β-actin (EM21002), anti-β-tubulin (ET1602-4), anti-Caspase1 (ET1608-69), anti-Bcl-2 (ST46-03), anti-SQSTM1/p62 (HA721171), anti-LC3B (ET1701-65), anti-Galectin-3 (ET1702-48); anti-RIPK3 (ab62344), anti-MLKL (ab243142), anti-Keap1 (ab227828), anti-ACSL4 (ab155282), anti-GSDMD (ab219800), anti-GSDME (ab215191), anti-Bax (ab32503), anti-Phalloidin-iFluor-488 (ab176753), anti-Lrp2/Megalin (ab184676), anti-SCL34A1 (ab151129), anti-F4/80 (ab16911), anti-CD3 (ab16669), Goat Anti-Mouse IgG H&L (Alexa Fluor® 488) (ab150113), Goat Anti-Mouse IgG H&L (Alexa Fluor® 594) (ab150116), Goat Anti-Rabbit IgG H&L (Alexa Fluor® 488) (ab150077), Goat Anti-Rabbit IgG H&L (Alexa

Fluor® 568) (ab175471) (Abcam); anti-GAPDH (60004-1-G), anti-GPX4 (67763-1-Ig), anti-Nrf2 (16396-1-AP), anti-GFP (66002-1-Ig); anti-APOB (Proteintech, 20578-1-AP), anti-CCN1 (HUABIO, ER1905-70), anti-CREB3L2 (HUABIO, ER63122), anti-CD11C (Invitrogen, 17-0114-82), anti-LAMP2 (ABclonal, A26781PM).

Cell culture and immunostaining

Primary proximal tubule epithelial cells (PTECs) were freshly isolated as previously described [2, 3]. After 4-5 days of culture, the PTECs were used for experimentation. Cells were cultured in Dulbecco's Modified Eagle's Medium (DMEM), supplemented with 10% fetal bovine serum, 4 mM L-glutamine, 100 IU/mL penicillin, and 100 mg/mL streptomycin. Cultures were maintained at 37°C in a humidified incubator with 5% CO2. After fixation, samples were rinsed twice with phosphate-buffered saline (PBS) and then permeabilized for 10 minutes using PBS containing 0.25% Triton X-100 (0.25% PT). To minimize nonspecific antibody binding, cells were blocked with 10% goat serum diluted in PBS containing 0.05% Tween-20 (0.05% PBST) for 1 hour. Primary antibodies were applied at a 1:200 dilution in blocking solution and incubated overnight at 4°C. On the following day, cells were washed three times with 0.05% PBST and then incubated with fluorophore-conjugated secondary antibodies (1:200 dilution in blocking buffer) for 45 minutes at room temperature. Nuclear staining was performed using DAPI for 15 minutes at room temperature. Fluorescent images were acquired using a ZEISS LSM800 confocal laser scanning microscope.

For the hypoxia-reoxygenation experiment (H/R), PTECs were seeded in 12-well plates and allowed to attach overnight at 37°C in a humidified incubator with 5% CO_2. To induce hypoxia, cells were switched to serum-free DMEM/F12 and placed in a hypoxic chamber equilibrated to 1% O_2, 94% N_2, and 5% CO_2 for 24 h. For reoxygenation, the medium was replaced with regular DMEM/F12 culture medium, and cells were returned to normoxic conditions (95% air and 5% CO_2) for an additional 36 h before downstream analyses.

For gene silencing experiments, PTECs were cultured in complete medium consisting of DMEM/F12 supplemented with 10% fetal bovine serum and antibiotics.

To induce *Pi4ka* deletion in vitro, PTECs were treated with 4-OHT (1 μM) for 4 consecutive days. Cells treated with vehicle (DMSO) were used as mock controls. PTECs were transfected with shRNA constructs targeting Creb3l2 (shCreb3l2-1# and shCreb3l2-2#) or a non-targeting control shRNA (shCon) using a calcium phosphate–based transfection method. Culture medium was replaced with fresh complete medium 24 h after transfection. For fluorescence imaging, cells were incubated with Fluo-4 AM to detect intracellular Ca^{2+} (green) and ER-Tracker Red to label the endoplasmic reticulum at 37°C for 30 min. Nuclei were counterstained with DAPI (blue) for 15 min prior to imaging.

Fluorescence imaging of lysosomal acidity, calcium, and ER in cells

Lysosomal acidity was assessed using lysotracker staining. Cells were incubated with 100 nM LysoTracker™ Green (Thermo Fisher, L7526) at 37°C for 15 minutes, following the manufacturer's guidelines. After incubation, cells were washed twice with pre-warmed PBS. Fluorescence was measured using a maximum excitation wavelength of 488 nm and a maximum emission wavelength of 510 nm. Images were acquired using a ZEISS LSM800 confocal microscope. Intracellular calcium concentration was measured using Fluo-4 AM. Cells were incubated with 5 μM Fluo-4 AM (Beyotime, S1060) at 37°C for 45 minutes. After incubation, cells were washed, then incubated for an additional 20-30 minutes to ensure complete conversion of Fluo-4 AM to Fluo-4. Fluorescence was measured using a maximum excitation wavelength of 494 nm and a maximum emission wavelength of 516 nm. Images were acquired using a ZEISS LSM800 confocal microscope. ER-tracker was used as fluorescent labeling of endoplasmic reticulum. Cell culture medium was removed and washed with warm HBSS buffer. Then cells were incubated 1 μM ER-tracker red (Beyotime, C1041S) at 37°C for 15-30 minutes. After incubation, cells were washed three times with warm HBSS buffer and imaged using a ZEISS LSM800 confocal microscope. The excitation wavelength was 587 nm, and the emission wavelength was 615 nm.

Histologic and immunofluorescence analysis of kidney sections

Kidney tissues were embedded either in paraffin or in optimal cutting temperature compound (Leica, 4538) for histological analysis. Paraffin-embedded sections were

stained with periodic acid–Schiff (PAS) reagent to evaluate renal tubular injury. For quantitative morphological assessment, at least 10 randomly selected fields per kidney section were examined at 100× magnification, following the methodology established in our previous work [1, 3-6]. Cryosections (4μm) were used for immunofluorescence staining. Sections were washed with Phosphate-Buffered Saline (PBS) after fixation with ice-cold acetone for 15min, then incubated with the following different primary antibodies: anti-PI4KA (12411-1-AP), anti-F4/80 (ab16911), and antibody for 2 − 4 h. As a detection antibody, AlexaFluor® 488 and Alexa Fluor® 594 labeled secondary antibodies (abcam) were used. Nucleus was labeled with 4',6-diamidino-2-phenylindole (DAPI, D3571, Invitrogen). All histologic sections were analyzed in a blinded manner. For morphologic quantifications, 3 random visual fields were analyzed per kidney section. The number of protein-positive cells were determined with Image J software.

Preparation of single-cell suspensions and generation of single-cell libraries for sequencing

Mice were euthanized, and kidneys were promptly excised and placed individually into pre-chilled Petri dishes containing cold PBS to preserve tissue integrity. Preparation of single-cell suspensions from kidney tissue, as well as single-cell library construction and sequencing, was performed following established protocols as previously described [1, 4, 7].

Quality control of single cell sequencing data

For kidney single-cell data, exclusion criteria were applied to remove cells that: (1) expressed ≤ 500 or ≥ 5000 genes per cell; (2) expressed ≤ 500 or ≥ 25000 UMIs per cell; (3) had a log10GenesPerUMI score ≤ 0.8; (4) displayed a mitochondrial ratio exceeding 30%. Following data quality control and filtering, low-quality single-cell data were eliminated.

Gene visualization and cell proportion analysis

Gene expression patterns were visualized utilizing the built-in visualization functions available in the Seurat package, including DotPlot, VlnPlot, and FeaturePlot.

To assess the spatial co-expression of two genes within the same cellular population, the "blend" parameter in the FeaturePlot function was set to TRUE, enabling the generation of composite expression maps. Comparative analysis of gene expression levels across multiple groups was conducted using one-way analysis of variance (ANOVA). For quantification of cell-type distribution, the proportion of each identified cell type was calculated within individual biological replicates across different experimental conditions. To evaluate the statistical significance of differences in cell composition between groups, a two-way ANOVA was performed.

Unsupervised dimensionality reduction, removal of batch effects, and cell type identification

The process of unsupervised dimensionality reduction and batch effect removal was consistent with the previous description [1, 7]. Cell types were identified by integrating top expressed genes and referencing the CellMarker database (http://117.50.127.228/CellMarker/). Clustering patterns were validated using heatmap analysis. Specific cell types were isolated using the Subset function, and analyses were performed to refine batch effect adjustments and identify clusters driven by biological differences.

Cell trajectory and transcription factor (TF) regulatory networks analysis

Cell trajectory analysis was conducted following the previously established protocol [2]. For transcription factor (TF) regulatory network inference, the PTC subpopulation was isolated and reclustered for downstream analysis. The pyscenic package (version 0.12.1) was employed in Python for regulon detection, with visualization performed in R. Log-normalized single-cell RNA-seq data were used as the input expression matrix. TF–target interactions were inferred using both GENIE3 and GRNBoost algorithms. Enrichment of TF binding motifs within target gene sets was identified via RcisTarget, and only modules containing significantly enriched motifs were retained to construct the TF–target regulatory network. Each transcription factor and its associated target genes constituted a "regulon" To quantify regulon activity across individual cells, the AUCell algorithm was applied, allowing the identification of cells in which specific regulons were active ("on").

Analysis of intercellular ligand-receptor communication

Intercellular communication via ligand–receptor interactions was analyzed using the CellPhoneDB Python package (version 5.0.1) [8]. Visualization of interaction networks was carried out in R using the ktplots package (version 2.0.0). Within the normalized single-cell RNA-seq dataset, we calculated the average expression levels of known ligand–receptor pairs between all combinations of cell types. To assess the statistical significance of these interactions, cell-type labels were randomly permuted 1,000 times to generate a null distribution, against which observed mean expression values were compared. Utilizing the Seurat-normalized expression matrix, statistically significant ligand–receptor interactions were identified based on a threshold of $P < 0.05$. Multiple hypothesis testing correction was applied using the Benjamini–Hochberg procedure to control the false discovery rate. Only interactions with statistically significant and biologically relevant expression levels were retained for further analysis and interpretation.

Gene enrichment analysis and scoring of pathway activity for cell subpopulations

GO term and KEGG pathway enrichment analyses were conducted using R with the clusterProfiler package (version 4.2.2). A list of differentially expressed genes (DEGs) was used as input, with gene symbols or Entrez IDs converted via the org.Mm.eg.db annotation package. Enrichment was performed separately for the Biological Process (BP), Molecular Function (MF), and Cellular Component (CC) categories in GO, as well as for KEGG pathways. The significance threshold was set at an adjusted p-value (Benjamini–Hochberg correction) < 0.05. Visualization of enrichment results was performed using ggplot2 and enrichplot packages, with dot plots and bar plots used to represent the top enriched terms. To assess pathway activity at the single-cell level within PTC subpopulations, we employed the AUCell package (version 1.16.0) to calculate the area under the curve (AUC) scores for predefined gene sets. AUCell evaluates whether a given gene set is enriched within the top-expressed genes of each individual cell, thereby estimating the relative activity of that pathway across cells. Normalized gene expression data were extracted from the RNA assay of the Seurat object. Gene sets representing specific biological pathways or cellular

programs (e.g., curated from MSigDB or literature sources) were compiled for input. AUCell rankings were generated using the AUCell_buildRankings() function, which ranks genes for each cell based on their expression levels. Subsequently, AUC scores were computed using the AUCell_calcAUC() function, quantifying the enrichment of each gene set in individual cells. The resulting AUC matrix was then incorporated into the metadata or assay slot of the Seurat object for downstream analysis and visualization. Pathway activity scores were visualized across PTC subclusters using dimensionality reduction plots (e.g., FeaturePlot) or violin plots (VlnPlot) to identify subpopulation-specific functional signatures.

Gene perturbation analysis

To evaluate the transcriptional role of *Creb3l2* in PTCs. we employed scTenifoldKnk (version 1.0.2) [9], a computational framework for virtual gene knockout analysis at single-cell resolution. This approach estimates the effect of gene deletion by reconstructing and comparing gene regulatory networks (GRNs) between perturbed and unperturbed conditions. Single-cell RNA-seq data from *Pi4ka*-deficient and control kidneys were preprocessed using Seurat, including quality control, normalization, log-transformation, scaling, and clustering. PTC subsets were extracted based on canonical marker expression (*Slc34a1*, *Lrp2*), and expression matrices were used as input for perturbation analysis. A virtual knockout of *Creb3l2* was simulated in PTCs, and GRNs were inferred using low-rank tensor decomposition to capture cell–gene interactions with reduced noise. Differential regulatory networks were generated by subtracting baseline GRNs from perturbed GRNs. The resulting Δregulon activity scores quantified the transcriptional shifts attributable to *Creb3l2* deletion. Genes showing significant deviations in regulon activity (false discovery rate [FDR] < 0.05) were defined as *Creb3l2*-dependent DEGs, representing direct or indirect transcriptional consequences of *Creb3l2* perturbation. These DEGs were subsequently used for downstream analyses, including pathway enrichment and regulatory module assessment.

Statistical analyses

All results represent data from at least three independent experiments. Statistical

analyses were performed using Prism software (GraphPad Software, Inc.). Data are expressed as mean ± SD. Group comparisons were made using an unpaired t-test, and for multiple comparisons, one-way ANOVA was employed. Statistical significance was defined as $P < 0.05$.

Supplemental Table 1: Clinical and Pathological Characteristics of Patients with AKI or without AKI

Variable	A	B	C	D	E	F	G	H	I	J	K	L
Age (years)	35	64	52	57	57	39	64	45	52	26	37	67
Sex (M/F)	M	F	M	M	M	M	F	F	M	F	M	M
Hypertension	0	1	0	1	1	1	1	0	1	0	0	0
Diabetes mellitus	0	0	0	0	0	0	0	0	0	0	1	0
RAASi	0	0	0	0	0	0	0	0	0	0	0	0
Diuretic use	1	0	0	0	0	0	0	0	0	0	0	0
Serum urea nitrogen (mmol/L)	15.07	25.60	15.26	18.40	11.42	34.70	6.20	3.96	7.50	3.90	4.89	4.10
Serum creatinine (µmol/L)	746.0	1285.0	532.0	373.1	322.0	872.2	60.2	52.0	65.2	51.7	56.0	51.70
eGFR(ml/min.1.73m^2)	7.47	2.36	9.84	14.59	17.55	5.97	92.69	112.73	106.20	128.17	126.49	105.14
AKI stage (2012 KDIGO)	3	3	3	3	2	3	-	-	-	-	-	-
Hemoglobin (g/L)	97	77	94	104	103	80	146	127	150	131	157	130
Serum albumin (g/L)	32	32.5	37.8	37.6	42.6	36.5	48.1	45.3	46.0	47.3	42.6	38.6
Total cholesterol (mmol/L)	4.17	5.12	3.63	3.88	3.99	5.52	6.28	4.45	4.89	4.34	4.07	2.69
Serum potassium (mmol/L)	5.08	5.65	4.81	4.12	3.33	5.27	4.09	4.40	3.84	4.09	5.30	5.04
Urine ACR (mg/g)	158.76	100.62	25.71	241.96	50.25	121.8	-	-	-	-	-	-
Urine occult blood (positive)	0	1	0	1	1	0	1	1	0	0	0	1

Renal pathology												
Tubular injury	1	1	1	1	1	1	0	0	0	0	0	0
Crystal deposition	1	0	1	0	0	0	0	0	0	0	0	0
Interstitial inflammation	1	1	1	1	1	1	0	0	0	0	0	0
Vascular lesions	0	0	0	0	0	0	0	0	0	0	0	0
Hemodialysis required	1	1	0	0	0	0	0	0	0	0	0	0
Off dialysis at 6 months after admission	1	1	1	1	1	1	-	-	-	-	-	-
Serum creatinine at 6 months (μmol/L) after admission	100	107	179	82.1	157	152.3	-	-	-	-	-	-

Notes: Individuals A–F represent patients with acute kidney injury (AKI); all underwent percutaneous renal biopsy. Individuals G–L represent non-AKI (Control) patients with clear cell renal cell carcinoma (ccRCC), and the adjacent non-tumor tissues were as used as control for further analysis. Unless otherwise specified, values are coded as 1 = yes/present, 0 = no/absent and '-' means not available.

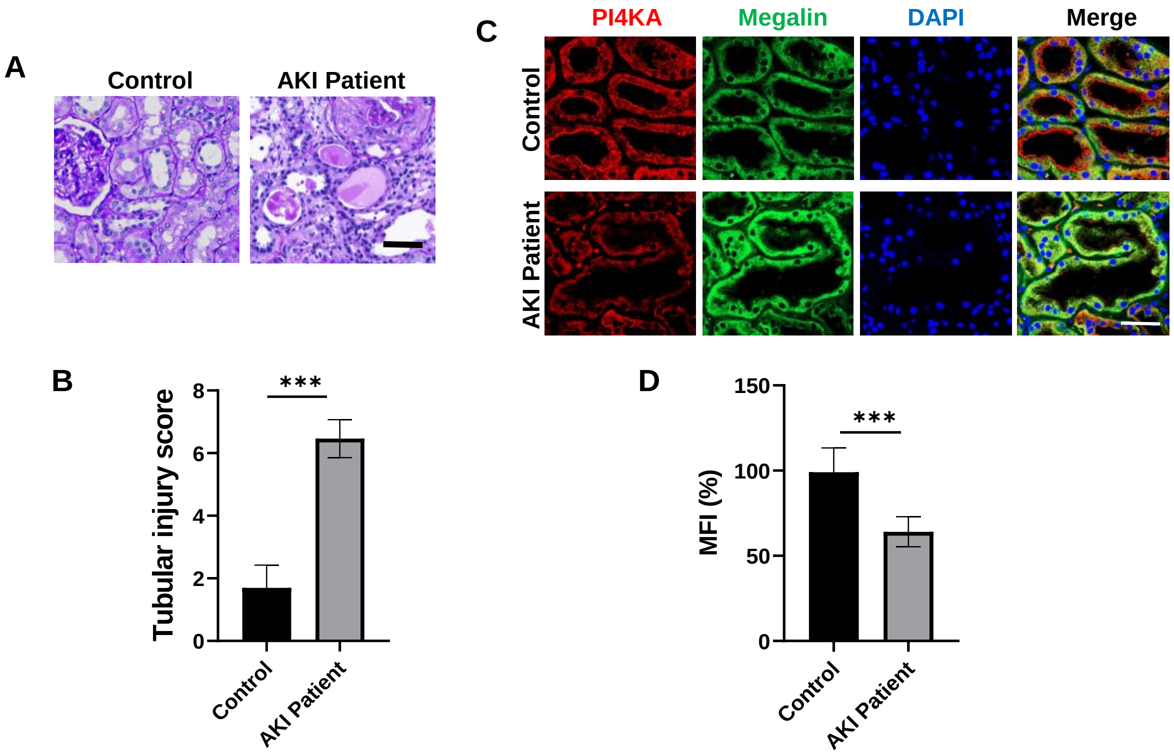

Supplementary Fig. 1. Tubular PI4KA protein is reduced in human AKI renal biopsy specimens.

(A) Representative PAS staining of renal biopsy sections from control and AKI patients, *n*=6 per group. Scale bars =100 μm . Control samples were obtained from non-tumorous adjacent renal tissue of patients undergoing nephrectomy for clear cell renal cell carcinoma (ccRCC).

(B) Quantification of tubular injury score corresponding to (A) in control and AKI patient renal biopsy specimens, $n = 6$ per group. Data are expressed as mean ± SD. ***$P < 0.001$.

(C) Representative immunofluorescence images showing PI4KA (red) and the proximal tubule marker megalin (green), with nuclei counterstained by DAPI (blue). Merged images indicate decreased PI4KA signal in megalin-positive proximal tubules in the AKI patients compared with the control patients, *n*=6 per group. Scale bars =50 μm .

(D) Quantification of PI4KA fluorescence intensity (MFI) corresponding to (C) in megalin-positive proximal tubules from control and AKI patient renal biopsy specimens, $n = 6$ per group. Data are expressed as mean ± SD. ***$P < 0.001$.

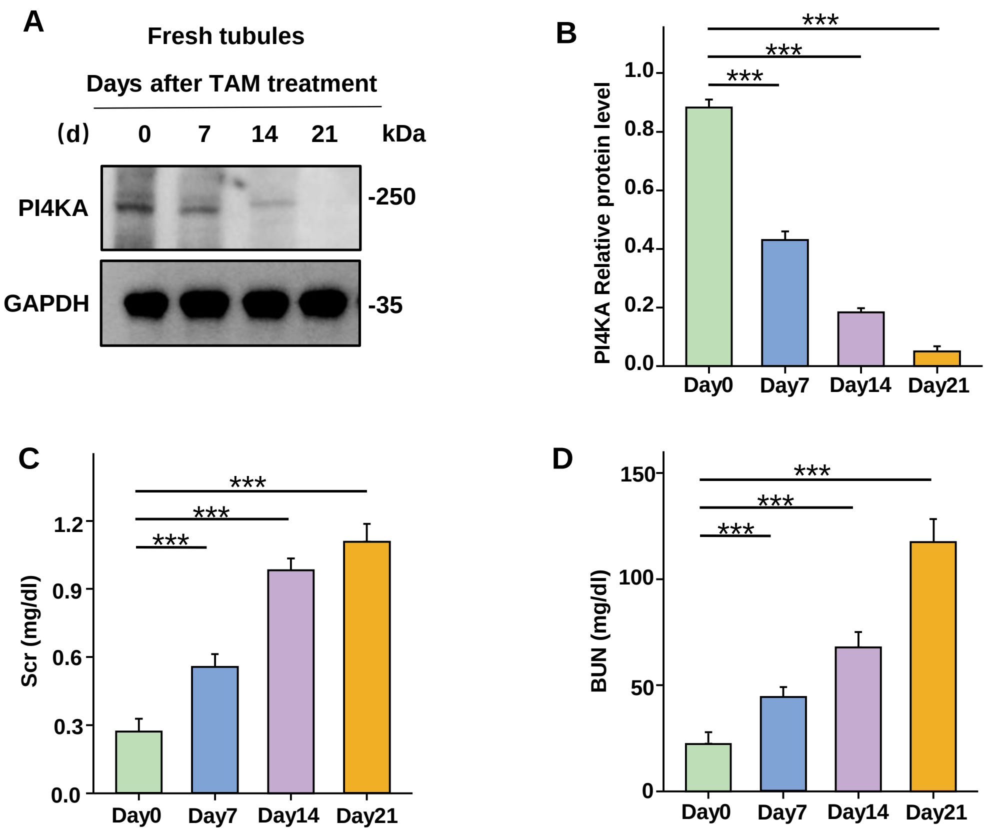

Supplementary Fig. 2. Tamoxifen-induced proximal tubule-specific *Pi4ka* deletion reduces PI4KA protein and impairs renal function.

(A) Representative immunoblot of freshly isolated renal tubules from *Pi4ka*$^{fl/fl}$ *Ksp*^{CreERT2} mice at the indicated days after tamoxifen administration, showing PI4KA protein levels, *n*=4 per group. GAPDH served as a loading control.

(B) Densitometric quantification of the immunoblot in panel A. PI4KA signals were normalized to GAPDH and expressed relative to Day 0, *n*=4 per group. Data are presented as mean ± SD, ****P* < 0.001.

(C-D) Serum creatinine (Scr) and blood urea nitrogen (BUN) levels at Day 0, 7, 14, and 21 after tamoxifen treatment, *n*=6 per group. Data are shown as mean ± SD, ****P* < 0.001.

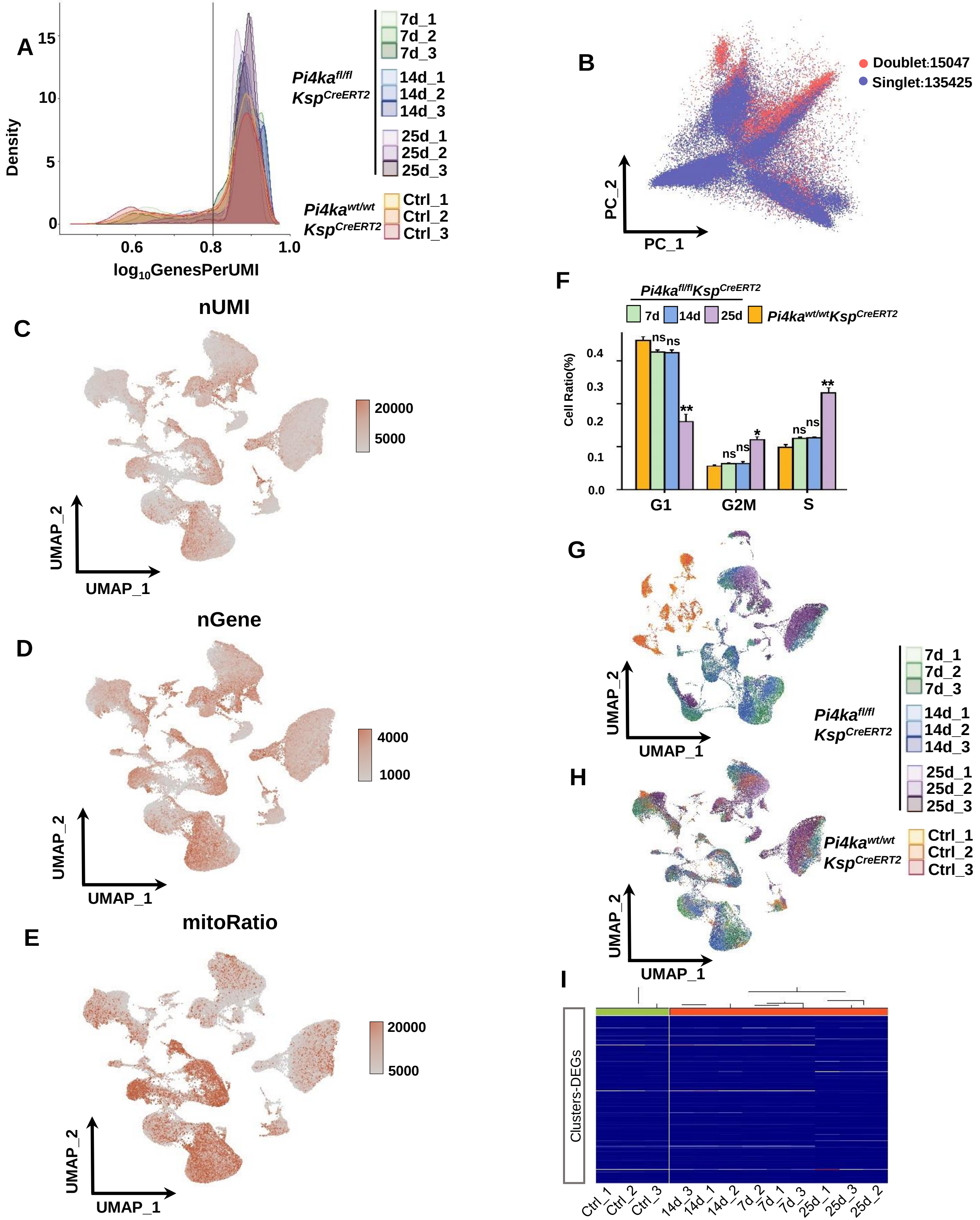

A
Density
log10GenesPerUMI
7d_1
7d_2
7d_3
14d_1
14d_2
14d_3
25d_1
25d_2
25d_3
Ctrl_1
Ctrl_2
Ctrl_3
Pi4ka^fl/fl Ksp^CreERT2
Pi4ka^wt/wt Ksp^CreERT2
B
Doublet:15047
Singlet:135425
PC_1
PC_2
C
nUMI
D
nGene
E
mitoRatio
UMAP_1
UMAP_2
F
Cell Ratio(%)
7d
14d
25d
G1
G2M
S
ns
*
**
G
H
I
Clusters-DEGs

Supplementary Fig. 3. Comprehensive quality control of scRNA data in *$Pi4ka^{fl/fl}Ksp^{CreERT2}$* mice.

(A) Density plot of $\log_{10}$ genes per UMI from single-cell RNA-seq data across different time points (7d, 14d, 25d) in *$Pi4ka^{fl/fl}Ksp^{CreERT2}$* and *$Pi4ka^{wt/wt}Ksp^{CreERT2}$* mice. The density plot shows the distribution of gene expression within each sample group, with distinct time points colored accordingly, n=3 per group.

(B) PCA plot showing the separation of doublets (red) and singlets (blue) within the dataset.

(C) UMAP plot showing the distribution of cells based on the number of unique molecular identifiers (nUMI) per cell, which represents cellular complexity and transcriptional activity.

(D) UMAP plot displaying the distribution of cells based on the number of genes detected per cell (nGene).

(E) UMAP plot showing the distribution of cells based on mitochondrial ratio (mitoRatio), a metric used to assess cell quality and potential stress or damage.

(F) Bar plot depicting the percentage of cells in different cell cycle phases (G1, G2M, and S) for *$Pi4ka^{fl/fl}Ksp^{CreERT2}$* and control samples at different time points (7d, 14d, 25d). *$P < 0.05$, **$P < 0.01$,ns: not significant.

(G) UMAP plot of *$Pi4ka^{fl/fl}Ksp^{CreERT2}$* cells colored by time point, showing the distribution of cells before batch effect removal.

(H) UMAP plot of control (*$Pi4ka^{wt/wt}Ksp^{CreERT2}$*) cells colored by time point, showing the distribution of cells after batch effect removal.

(I) Heatmap of differentially expressed genes (DEGs) across clusters and time points, with clustering based on DEGs, effectively distinguishing between control and knockout samples. The clustering within the knockout group is driven by their respective differences in gene expression.

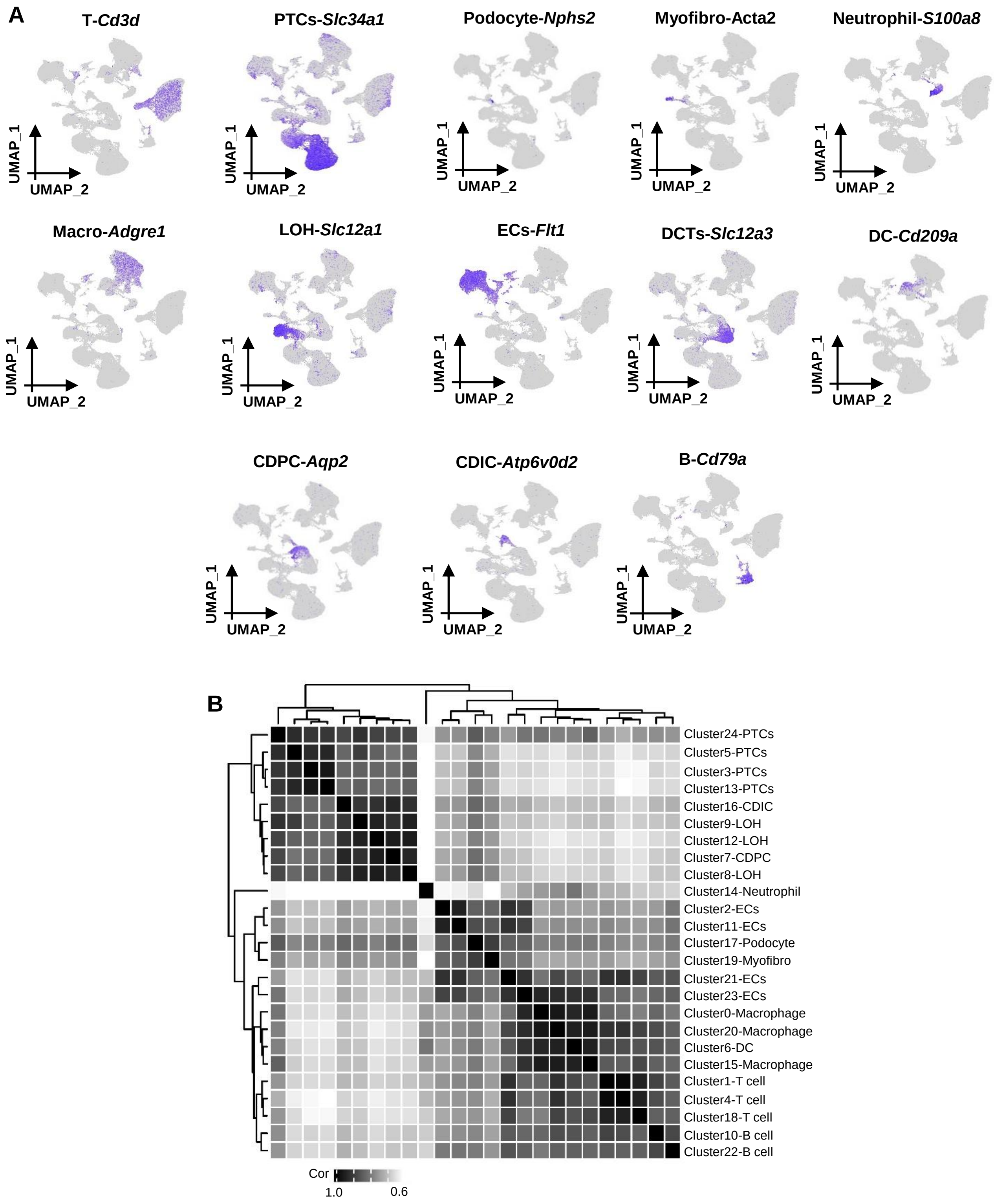
A
T-Cd3d
PTCs-Slc34a1
Podocyte-Nphs2
Myofibro-Acta2
Neutrophil-S100a8
Macro-Adgre1
LOH-Slc12a1
ECs-Flt1
DCTs-Slc12a3
DC-Cd209a
CDPC-Aqp2
CDIC-Atp6v0d2
B-Cd79a
UMAP_1
UMAP_2
B
Cluster24-PTCs
Cluster5-PTCs
Cluster3-PTCs
Cluster13-PTCs
Cluster16-CDIC
Cluster9-LOH
Cluster12-LOH
Cluster7-CDPC
Cluster8-LOH
Cluster14-Neutrophil
Cluster2-ECs
Cluster11-ECs
Cluster17-Podocyte
Cluster19-Myofibro
Cluster21-ECs
Cluster23-ECs
Cluster0-Macrophage
Cluster20-Macrophage
Cluster6-DC
Cluster15-Macrophage
Cluster1-T cell
Cluster4-T cell
Cluster18-T cell
Cluster10-B cell
Cluster22-B cell
Cor
1.0
0.6

Supplementary Fig. 4. Identification of cell types based on marker gene expression in *Pi4ka*$^{fl/fl}$*Ksp*^{CreERT2} mice.

(A) UMAP plots showing the expression of specific marker genes for various cell types in PIK4A deficient mice. Each plot represents the expression of a marker gene in different cell populations, with high expression shown in purple.

(B) Heatmap showing the correlation of gene expression between different clusters identified in the scRNA-seq data. Clusters represent different cell types and their gene expression profiles, with PTCs and other immune cells, such as macrophages and T cells, showing distinct expression patterns. The correlation matrix highlights the relationships between these clusters based on shared gene expression features.

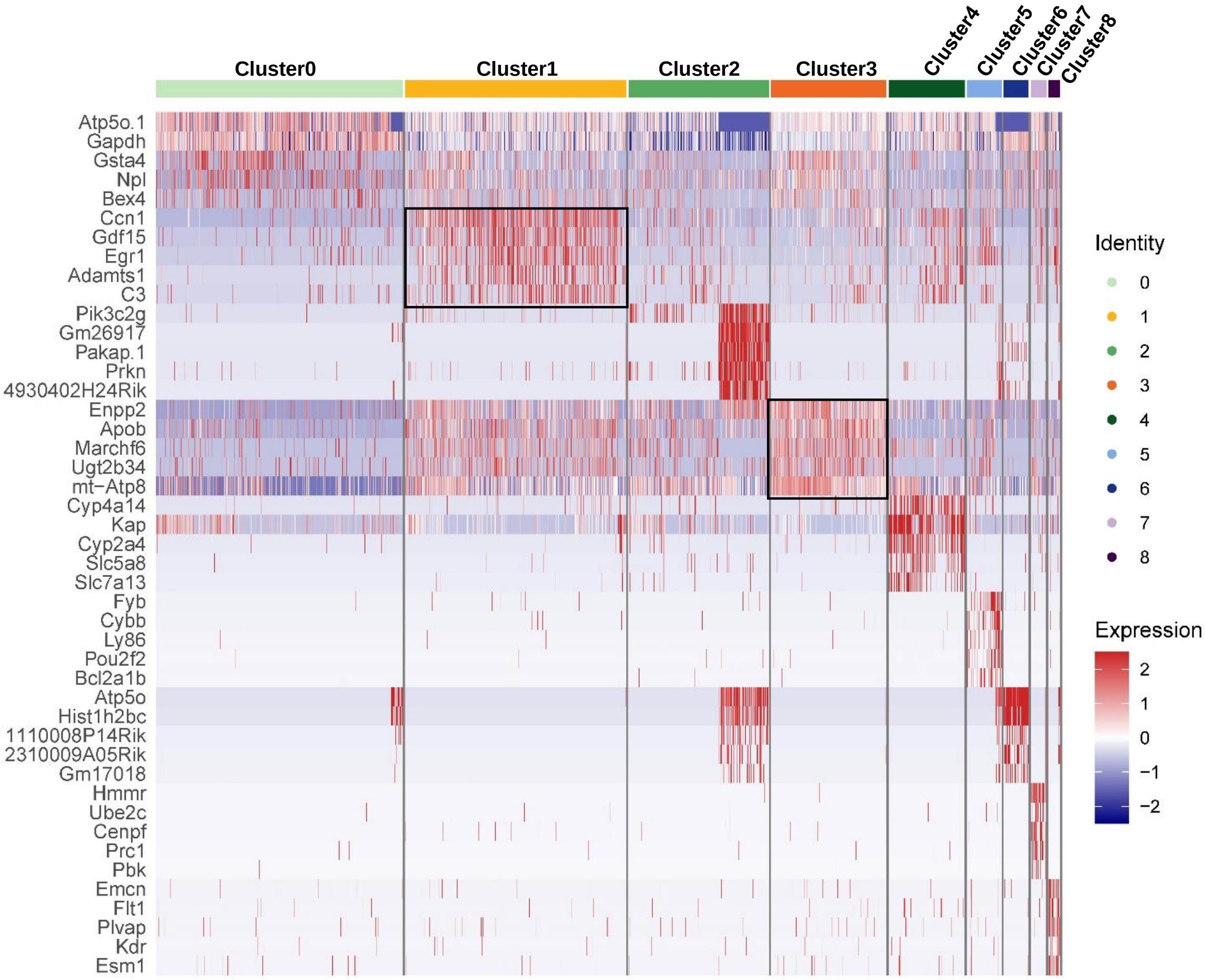

Supplementary Fig. 5. Molecular profiling of PTCs subpopulations through reclustering analysis

Unsupervised clustering of PTCs identified 9 transcriptionally distinct subpopulations, with the top five differentially expressed genes (DEGs) for each cluster visualized using the DoHeatmap function.

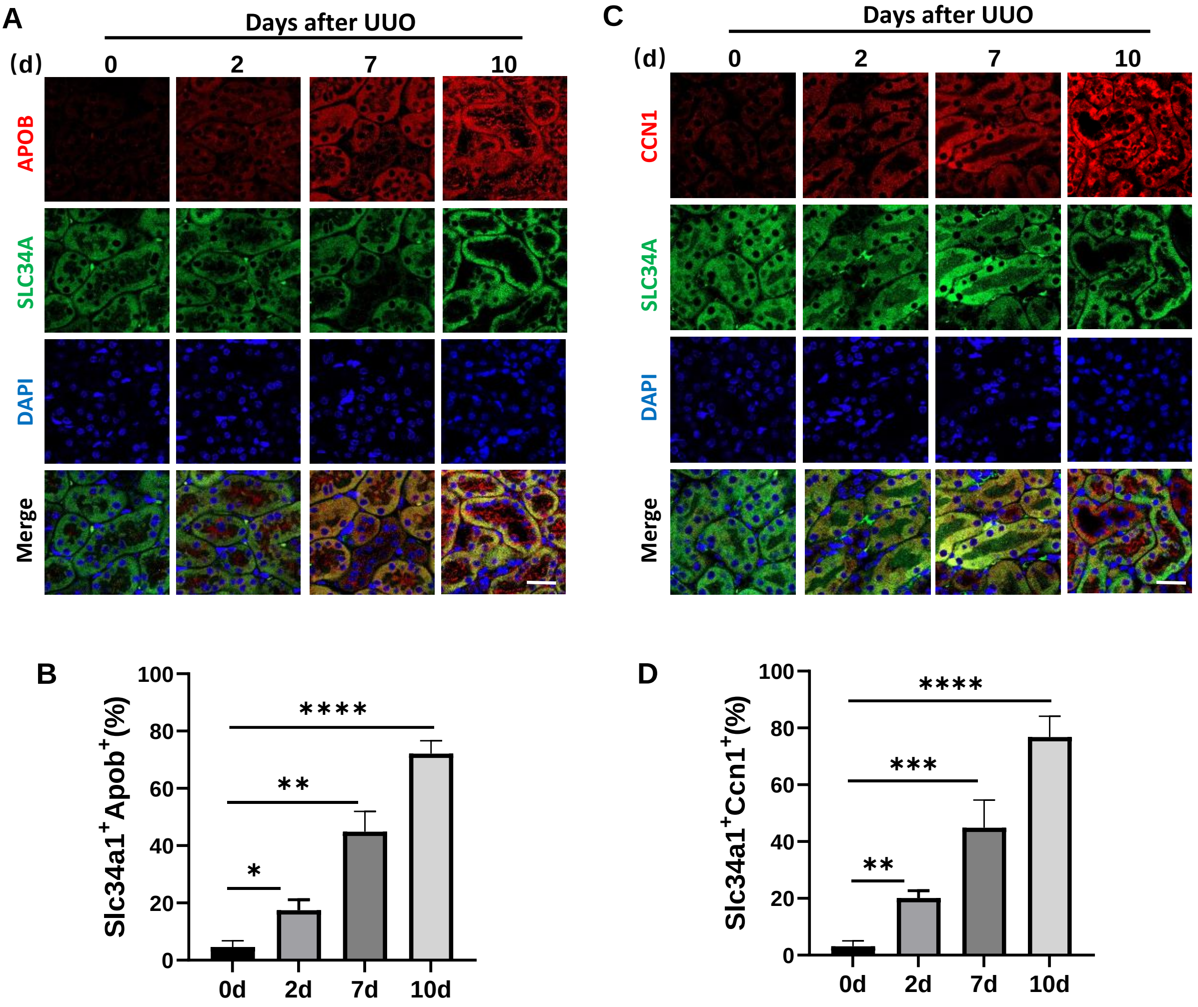

Supplementary Fig. 6. Immunofluorescence validation of *Slc34a1*$^{+}$*Apob*$^{+}$ and *Slc34a1*$^{+}$*Ccn1*$^{+}$ proximal tubule cell states during UUO.

(A, C) Representative immunofluorescence images of kidney sections collected at the indicated days after unilateral ureteral obstruction (UUO), stained for the proximal tubule marker SLC34A1 (green) together with either APOB (red, A) or CCN1 (red, panel B). Nuclei were counterstained with DAPI (blue). Merged images show APOB or CCN1 signals within SLC34A1-positive proximal tubules, *n*=6 per group. Scale bars =50μm.

(B, D) Quantification of *Slc34a1*$^{+}$*ApoE*$^{+}$ and *Slc34a1*$^{+}$*Ccn1*$^{+}$ cells corresponding to (A) and (C), respectively, at the indicated days after UUO, *n* = 6 per group. Data are expressed as mean ± SD. *$P < 0.05$, **$P < 0.01$, ***$P < 0.001$, ****$P < 0.0001$.

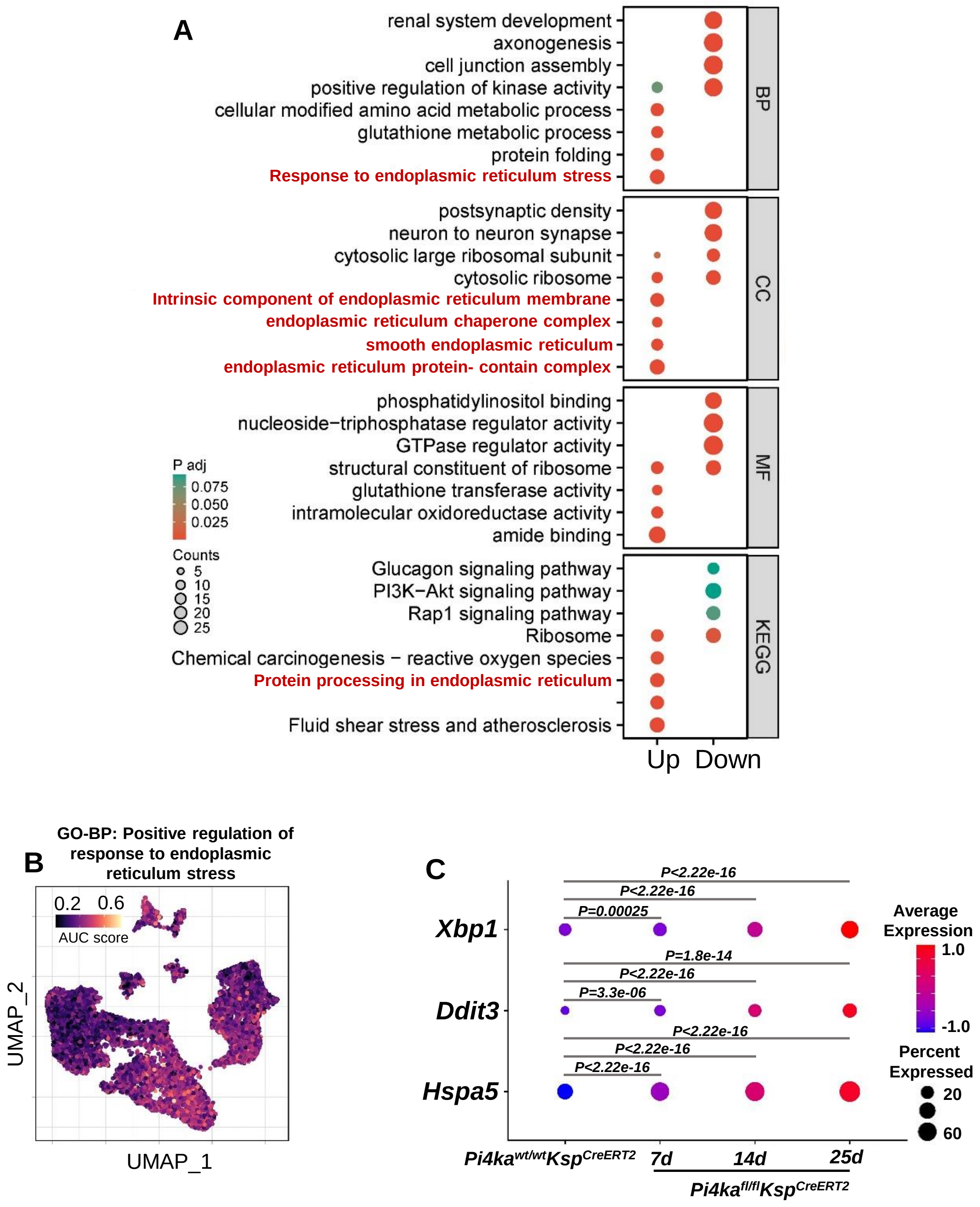
A
renal system development
axonogenesis
cell junction assembly
positive regulation of kinase activity
cellular modified amino acid metabolic process
glutathione metabolic process
protein folding
Response to endoplasmic reticulum stress
postsynaptic density
neuron to neuron synapse
cytosolic large ribosomal subunit
cytosolic ribosome
Intrinsic component of endoplasmic reticulum membrane
endoplasmic reticulum chaperone complex
smooth endoplasmic reticulum
endoplasmic reticulum protein- contain complex
phosphatidylinositol binding
nucleoside−triphosphatase regulator activity
GTPase regulator activity
structural constituent of ribosome
glutathione transferase activity
intramolecular oxidoreductase activity
amide binding
Glucagon signaling pathway
PI3K−Akt signaling pathway
Rap1 signaling pathway
Ribosome
Chemical carcinogenesis − reactive oxygen species
Protein processing in endoplasmic reticulum
Fluid shear stress and atherosclerosis
BP
CC
MF
KEGG
Up
Down
P adj
0.075
0.050
0.025
Counts
5
10
15
20
25
B
GO-BP: Positive regulation of response to endoplasmic reticulum stress
0.2
0.6
AUC score
UMAP_2
UMAP_1
C
Xbp1
Ddit3
Hspa5
P<2.22e-16
P<2.22e-16
P=0.00025
P=1.8e-14
P<2.22e-16
P=3.3e-06
P<2.22e-16
P<2.22e-16
P<2.22e-16
Average Expression
1.0
-1.0
Percent Expressed
20
60
Pi4ka^wt/wt Ksp^CreERT2
7d
14d
25d
Pi4ka^fl/fl Ksp^CreERT2

Supplementary Fig. 7. Early activation of endoplasmic reticulum stress pathways in PTCs of *Pi4ka*$^{fl/fl}$*Ksp*^{CreERT2} Mice

(A) Differential gene enrichment analysis showing that in the early stages (7 days) of *Pi4ka*-deficient mouse kidney injury, a large number of endoplasmic reticulum (ER) stress-related pathways are activated in PTCs.

(B) AUCell scoring analysis shows the activation levels of ER stress-related pathways at different time points, with results revealing the activation of ER stress pathways at day 7, which progressively increases over time (14 and 25 days).

(C) Differential expression analysis of ER stress-related proteins, such as *Xbp1*, *Ddit3*, and *Hspa5*. ER stress genes are activated at the first time point (7 days) of injury, with expression levels gradually increasing at later time points.

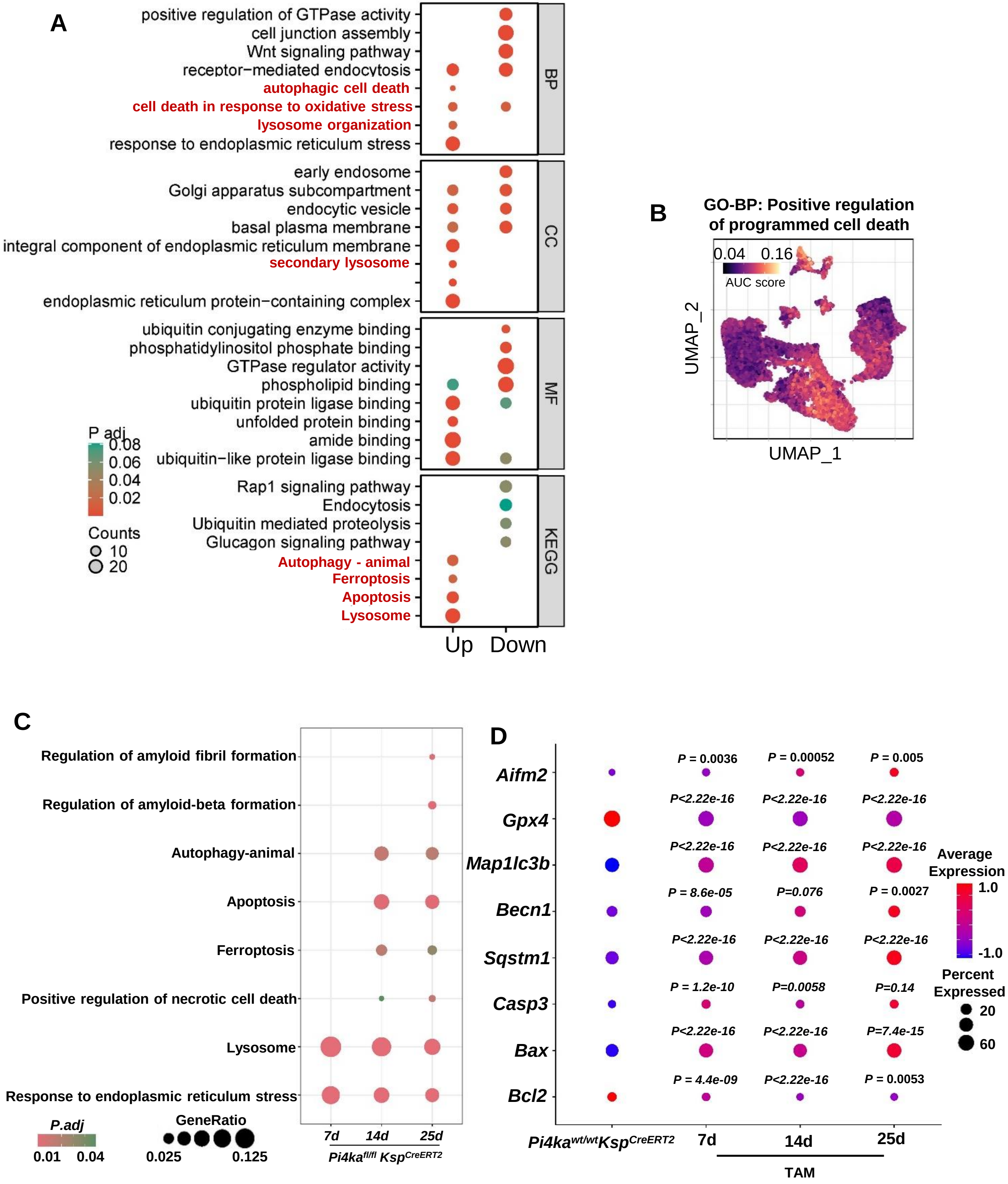
A
positive regulation of GTPase activity
cell junction assembly
Wnt signaling pathway
receptor−mediated endocytosis
autophagic cell death
cell death in response to oxidative stress
lysosome organization
response to endoplasmic reticulum stress
BP
early endosome
Golgi apparatus subcompartment
endocytic vesicle
basal plasma membrane
integral component of endoplasmic reticulum membrane
secondary lysosome
endoplasmic reticulum protein−containing complex
CC
ubiquitin conjugating enzyme binding
phosphatidylinositol phosphate binding
GTPase regulator activity
phospholipid binding
ubiquitin protein ligase binding
unfolded protein binding
amide binding
ubiquitin−like protein ligase binding
MF
Rap1 signaling pathway
Endocytosis
Ubiquitin mediated proteolysis
Glucagon signaling pathway
Autophagy - animal
Ferroptosis
Apoptosis
Lysosome
KEGG
Up
Down
P adj
0.08
0.06
0.04
0.02
Counts
10
20
B
GO-BP: Positive regulation of programmed cell death
0.04
0.16
AUC score
UMAP_2
UMAP_1
C
Regulation of amyloid fibril formation
Regulation of amyloid-beta formation
Autophagy-animal
Apoptosis
Ferroptosis
Positive regulation of necrotic cell death
Lysosome
Response to endoplasmic reticulum stress
P.adj
0.01
0.04
GeneRatio
0.025
0.125
7d
14d
25d
Pi4ka fl/fl Ksp CreERT2
D
Aifm2
Gpx4
Map1lc3b
Becn1
Sqstm1
Casp3
Bax
Bcl2
P = 0.0036
P = 0.00052
P = 0.005
P<2.22e-16
P<2.22e-16
P<2.22e-16
P<2.22e-16
P<2.22e-16
P<2.22e-16
P = 8.6e-05
P=0.076
P = 0.0027
P<2.22e-16
P<2.22e-16
P<2.22e-16
P = 1.2e-10
P=0.0058
P=0.14
P<2.22e-16
P<2.22e-16
P=7.4e-15
P = 4.4e-09
P<2.22e-16
P = 0.0053
Average Expression
1.0
-1.0
Percent Expressed
20
60
Pi4ka wt/wt Ksp CreERT2
7d
14d
25d
TAM

Supplementary Fig. 8. Time-dependent activation of regulated cell death pathways in PTCs of *Pi4ka*$^{fl/fl}$*Ksp*^{CreERT2} mice at mid-term (14 Days) post-TAM treatment.

(A) Differential gene enrichment analysis showing that in *Pi4ka*-deficient mouse kidneys at mid-term (14 days), several regulated cell death pathways (such as ferroptosis and autophagy) are significantly activated in PTCs.

(B) UMAP visualization of selective autophagy pathway activation in *Pi4ka*$^{fl/fl}$*Ksp*^{CreERT2} mouse kidneys, demonstrating the spatial distribution of cells with elevated autophagy pathway activity.

(C) The bubble chart shows the stage-specific activation of signaling pathways following PTC-specific *Pi4ka* deletion. Early activation (7d) includes lysosomal and endoplasmic reticulum stress pathways. Mid-stage (14d) activation involves cell death-related pathways, while late-stage (25d) activation is associated with amyloid fibril formation pathways.

(D) Expression of key genes associated with ferroptosis and autophagy (such as *Aifm2*, *Gpx4*, *Map1lc3b*, *Beclin1*, *Sgqst3*, *Casp3*, and *Bcl2*) in *Pi4ka*$^{fl/fl}$*Ksp*^{CreERT2} mice at 7, 14, and 25 days post-TAM treatment.

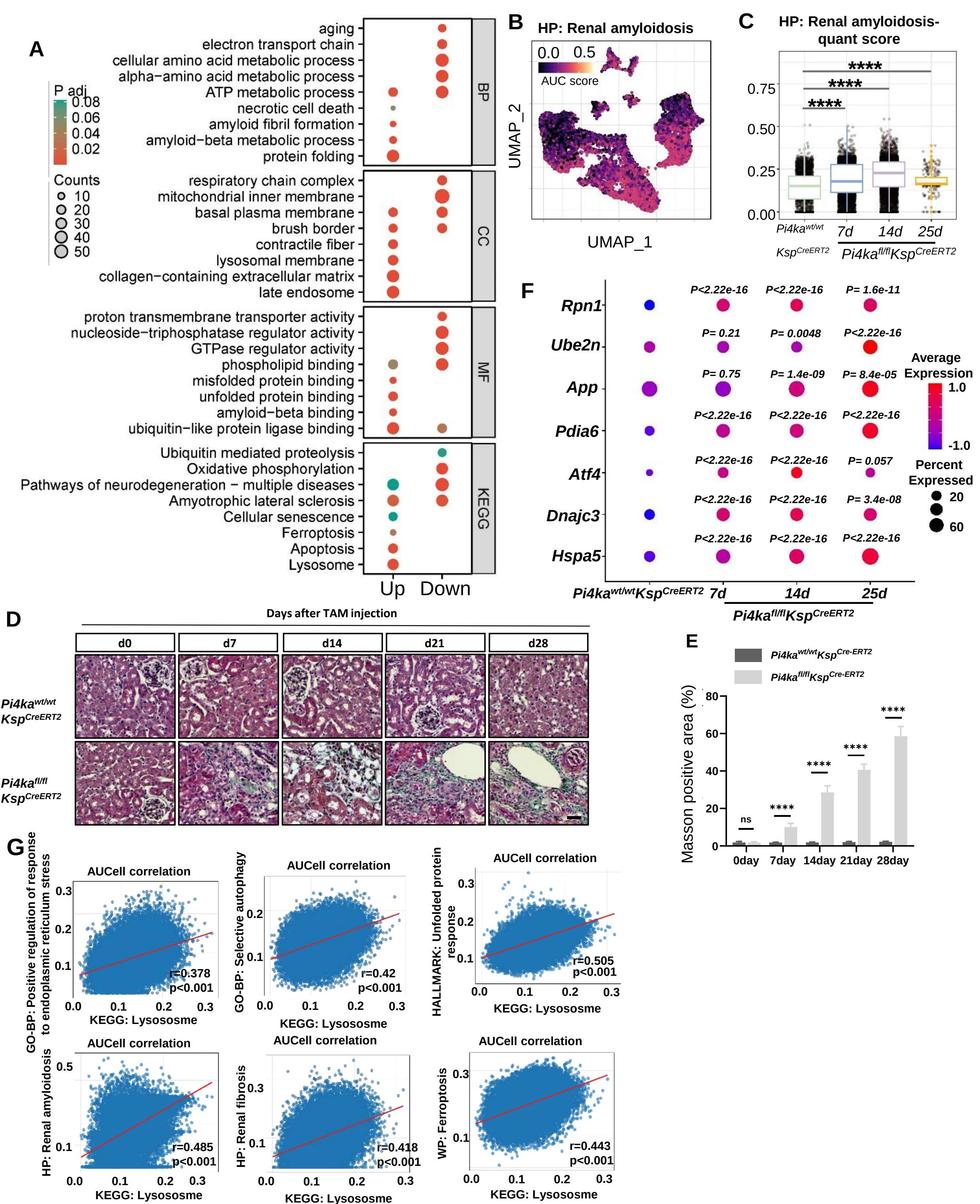

A
P adj
Counts
aging
electron transport chain
cellular amino acid metabolic process
alpha−amino acid metabolic process
ATP metabolic process
necrotic cell death
amyloid fibril formation
amyloid−beta metabolic process
protein folding
respiratory chain complex
mitochondrial inner membrane
basal plasma membrane
brush border
contractile fiber
lysosomal membrane
collagen−containing extracellular matrix
late endosome
proton transmembrane transporter activity
nucleoside−triphosphatase regulator activity
GTPase regulator activity
phospholipid binding
misfolded protein binding
unfolded protein binding
amyloid−beta binding
ubiquitin−like protein ligase binding
Ubiquitin mediated proteolysis
Oxidative phosphorylation
Pathways of neurodegeneration − multiple diseases
Amyotrophic lateral sclerosis
Cellular senescence
Ferroptosis
Apoptosis
Lysosome
BP
CC
MF
KEGG
Up
Down
B
HP: Renal amyloidosis
AUC score
UMAP_1
UMAP_2
C
HP: Renal amyloidosis-quant score
****
Pi4ka wt/wt Ksp CreERT2
7d
14d
25d
Pi4ka fl/fl Ksp CreERT2
F
Rpn1
Ube2n
App
Pdia6
Atf4
Dnajc3
Hspa5
P<2.22e-16
P= 1.6e-11
P= 0.21
P= 0.0048
P= 0.75
P= 1.4e-09
P= 8.4e-05
P= 0.057
P= 3.4e-08
Average Expression
Percent Expressed
D
Days after TAM injection
d0
d7
d14
d21
d28
E
Masson positive area (%)
ns
0day
7day
14day
21day
28day
G
AUCell correlation
KEGG: Lysosome
GO-BP: Positive regulation of response to endoplasmic reticulum stress
GO-BP: Selective autophagy
HALLMARK: Unfolded protein response
HP: Renal amyloidosis
HP: Renal fibrosis
WP: Ferroptosis
r=0.378
r=0.42
r=0.505
r=0.485
r=0.418
r=0.443
p<0.001

Supplementary Fig. 9. Activation of protein folding dysregulation and renal amyloidosis pathways in PTCs of *Pi4ka*$^{fl/fl}$*Ksp*^{CreERT2} mice at late-term (25 Days) post-TAM treatment

(A) Differential gene enrichment analysis reveals that at the late-term (25 days) in *Pi4ka*-deficient mouse kidneys, significant activation of protein folding dysregulation and renal amyloidosis pathways occurs in PTCs, suggesting that these pathways may be key mechanisms of late-stage kidney injury.

(B) AUCell scoring analysis of the renal amyloidosis pathway shows activation at early (7 days) and mid-term (14 days) stages but a significant downregulation at the late stage (25 days), suggesting that amyloidosis may not be a major mechanism in late-stage kidney injury.

(C) Quantification of the renal amyloidosis pathway at 7, 14, and 25 days shows activation at early (7d) and mid-term (14d) stages, with a significant downregulation at 25 days, suggesting a reduced role of amyloidosis in late-stage kidney injury.

(D) Masson's trichrome staining of kidney tissue sections from *Pi4ka*$^{fl/fl}$*Ksp*^{CreERT2} mice reveals a progressive increase in renal fibrosis as the injury time extends (0, 7, 14, 21, 28 days), indicating ongoing fibrosis progression during late-stage kidney injury, n=6 per group. Scale bars =100μm.

(E) Quantification of fibrosis shows significantly higher renal injury in *Pi4ka*$^{fl/fl}$*Ksp*^{CreERT2} mice at 7, 14, 21, and 28 days compared to *Pi4ka*$^{wt/wt}$*Ksp*^{CreERT2} mice, n=6 per group. Data are shown as mean ± SD, ****$P < 0.0001$, *ns*= not significant.

(F) Differential expression analysis of genes associated with protein folding dysregulation shows a time-dependent increase in their expression at the late stage (25 days).

(G) Correlation analyses revealed that lysosomal signaling activation was positively associated with ER stress, regulated cell death, unfolded protein response, and renal fibrosis pathways.

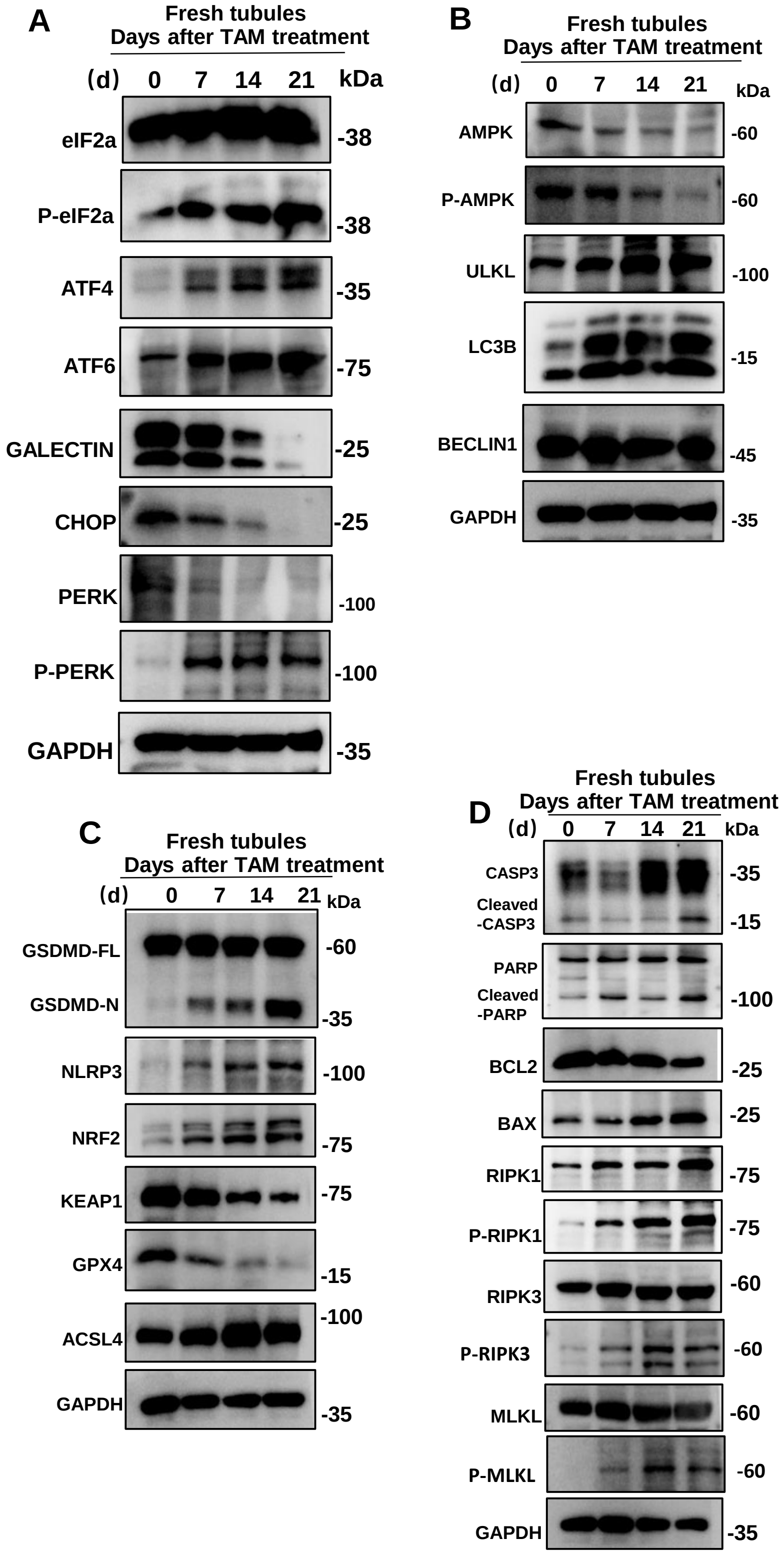
A
Fresh tubules
Days after TAM treatment
(d) 0 7 14 21 kDa
eIF2a -38
P-eIF2a -38
ATF4 -35
ATF6 -75
GALECTIN -25
CHOP -25
PERK -100
P-PERK -100
GAPDH -35
B
Fresh tubules
Days after TAM treatment
(d) 0 7 14 21 kDa
AMPK -60
P-AMPK -60
ULKL -100
LC3B -15
BECLIN1 -45
GAPDH -35
C
Fresh tubules
Days after TAM treatment
(d) 0 7 14 21 kDa
GSDMD-FL -60
GSDMD-N -35
NLRP3 -100
NRF2 -75
KEAP1 -75
GPX4 -15
ACSL4 -100
GAPDH -35
D
Fresh tubules
Days after TAM treatment
(d) 0 7 14 21 kDa
CASP3 -35
Cleaved -CASP3 -15
PARP
Cleaved -PARP -100
BCL2 -25
BAX -25
RIPK1 -75
P-RIPK1 -75
RIPK3 -60
P-RIPK3 -60
MLKL -60
P-MLKL -60
GAPDH -35

Supplementary Fig. 10. Immunoblotting analyses of freshly isolated renal tubules at indicated time points.

(A-D) ER stress markers (eIF2α, ATF4, ATF6, CHOP) (A), autophagy-related proteins (AMPK, ULK1, LC3B, BECLIN1) (B), ferroptosis- and pyroptosis–associated proteins (GSDMD, NLRP3, NRF2, KEAP1, GPX4, ACSL4) (C), and apoptosis/necroptosis regulators (CASP3, BCL2, BAX, RIPK1/3, MLKL) (D) were activated, n=4 per group. GAPDH served as a loading control.

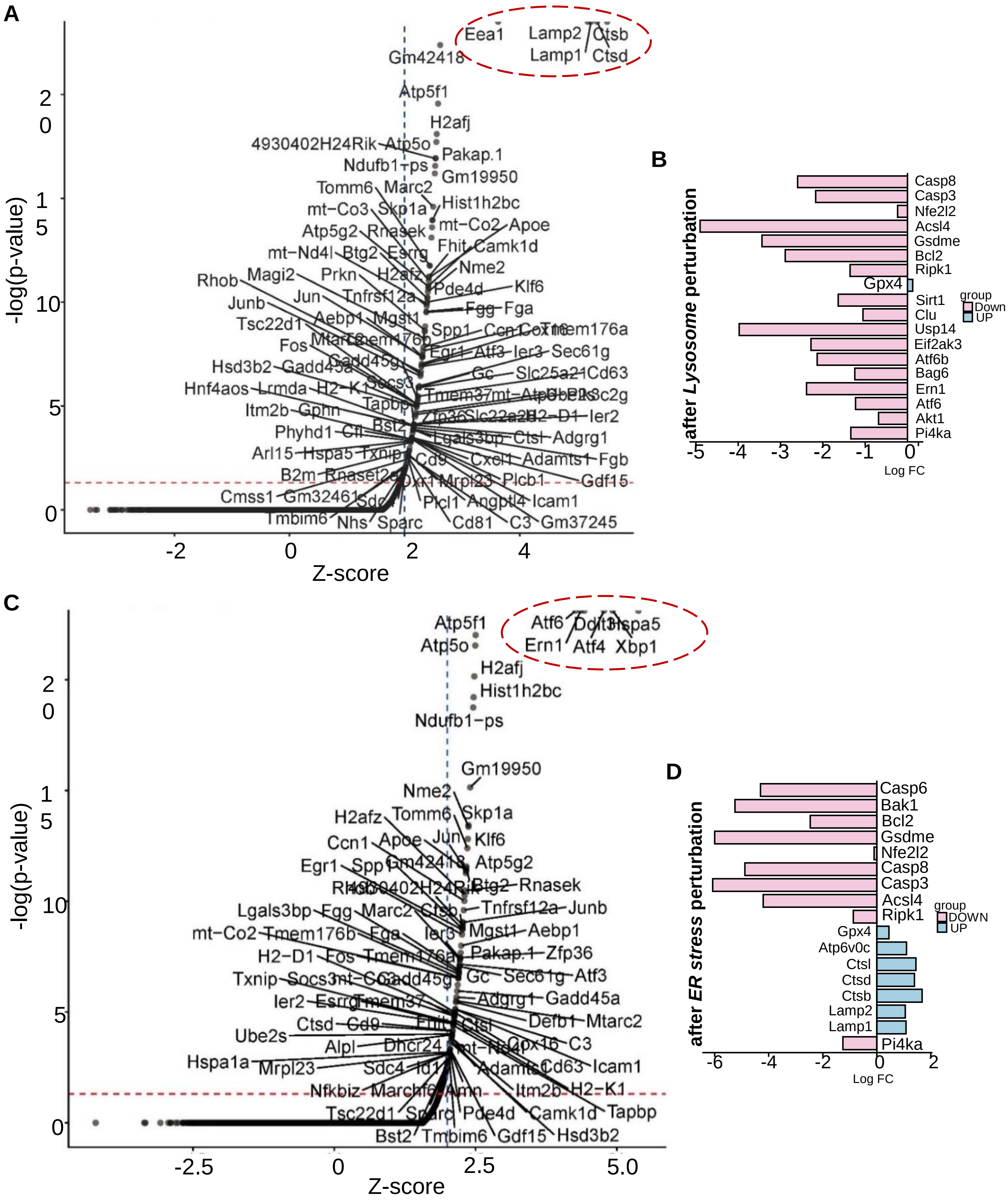

A
-log(p-value)
Z-score
Eea1
Lamp2
Ctsb
Lamp1
Ctsd
Gm42418
Atp5f1
H2afj
4930402H24Rik
Atp5o
Pakap.1
Ndufb1−ps
Gm19950
Tomm6
Marc2
Hist1h2bc
mt−Co3
Skp1a
mt−Co2
Apoe
Atp5g2
Rnasek
Fhit
Camk1d
mt−Nd4l
Btg2
Esrrg
Nme2
Rhob
Magi2
Prkn
H2afz
Pde4d
Klf6
Junb
Jun
Tnfrsf12a
Fgg
Fga
Tsc22d1
Aebp1
Mgst1
Spp1
Fos
Egr1
Atf3
Ier3
Sec61g
Hsd3b2
Gadd45a
Gadd45g
Gc
Slc25a21
Cd63
Hnf4aos
Lrmda
H2−K1
Socs3
Tapbp
Itm2b
Gphn
Zfp36
Ier2
Phyhd1
Cfl
Bst2
Lgals3bp
Ctsl
Adgrg1
Arl15
Hspa5
Txnip
Cd9
Cxcl1
Adamts1
Fgb
B2m
Rnaset2
Mrpl23
Plcb1
Gdf15
Cmss1
Plcl1
Angptl4
Icam1
Tmbim6
Nhs
Sparc
Cd81
C3
Gm37245
B
after Lysosome perturbation
Casp8
Casp3
Nfe2l2
Acsl4
Gsdme
Bcl2
Ripk1
Gpx4
Sirt1
Clu
Usp14
Eif2ak3
Atf6b
Bag6
Ern1
Atf6
Akt1
Pi4ka
group
Down
UP
Log FC
C
-log(p-value)
Z-score
Atf6
Ddit3
Hspa5
Ern1
Atf4
Xbp1
Atp5f1
Atp5o
H2afj
Hist1h2bc
Ndufb1−ps
Gm19950
Nme2
H2afz
Tomm6
Skp1a
Ccn1
Apoe
Jun
Klf6
Egr1
Spp1
Gm42418
Atp5g2
Btg2
Rnasek
Lgals3bp
Egg
Marc2
Ctsb
Tnfrsf12a
Junb
mt−Co2
Tmem176b
Fga
Ier3
Mgst1
Aebp1
Pakap.1
Zfp36
H2−D1
Fos
Gc
Sec61g
Atf3
Txnip
Socs3
Gadd45g
Ier2
Esrrg
Tmem37
Adgrg1
Gadd45a
Ctsd
Cd9
Ctsl
Defb1
Mtarc2
Ube2s
Alpl
Dhcr24
Cpx16
C3
Hspa1a
Mrpl23
Sdc4
Id1
Cd63
Icam1
Nfkbiz
Amn
Itm2b
H2−K1
Tsc22d1
Sparc
Pde4d
Camk1d
Tapbp
Bst2
Tmbim6
Gdf15
Hsd3b2
D
after ER stress perturbation
Casp6
Bak1
Bcl2
Gsdme
Nfe2l2
Casp8
Casp3
Acsl4
Ripk1
Gpx4
Atp6v0c
Ctsl
Ctsd
Ctsb
Lamp2
Lamp1
Pi4ka
group
DOWN
UP
Log FC

Supplementary Fig. 11. Gene perturbation analysis and its impact on lysosome or ER stress pathway.

(A) Gene perturbation analysis using scTenifoldKnk following lysosome-related gene interference. The plot illustrates the differential expression of genes, with genes associated with lysosome signaling (highlighted in the red circle) showing significant changes.

(B) Z-score analysis of gene expression changes following lysosome pathway perturbation reveals significant downregulation of genes involved in endoplasmic reticulum (ER) stress (*Sirt1, Clu, Usp14, Eif2ak3, Atf6b, Bag6, Ern1 , Atf6 , Akt1*) and cell death (*Casp8, Casp3, Nfe2l2, Acsl4, Gsdme, Bcl2, Ripk1*). These results indicate a possible link between lysosome dysfunction and reduced activation of ER stress and apoptotic pathways, suggesting that lysosomal signaling may play a role in regulating cellular stress responses.

(C) Gene perturbation analysis using scTenifoldKnk following ER stress-related gene interference. The plot illustrates the differential expression of genes, with genes associated with lysosome signaling (highlighted in the red circle) showing significant changes.

(D) Z-score analysis of gene expression changes following ER stress pathway perturbation reveals no change in the expression direction of genes involved in the lysosome pathway (*Lamp1, Lamp2, Ctsb, Ctsd, Ctsl, Atp6v0c*). However, there is downregulation of cell death-related genes (*Casp6, Casp8, Casp3, Nfe2l2, Acsl4, Gsdme, Bcl2*, *Bak1*, *Ripk1*), suggesting that ER stress activation affects cell survival pathways without directly influencing lysosomal function.

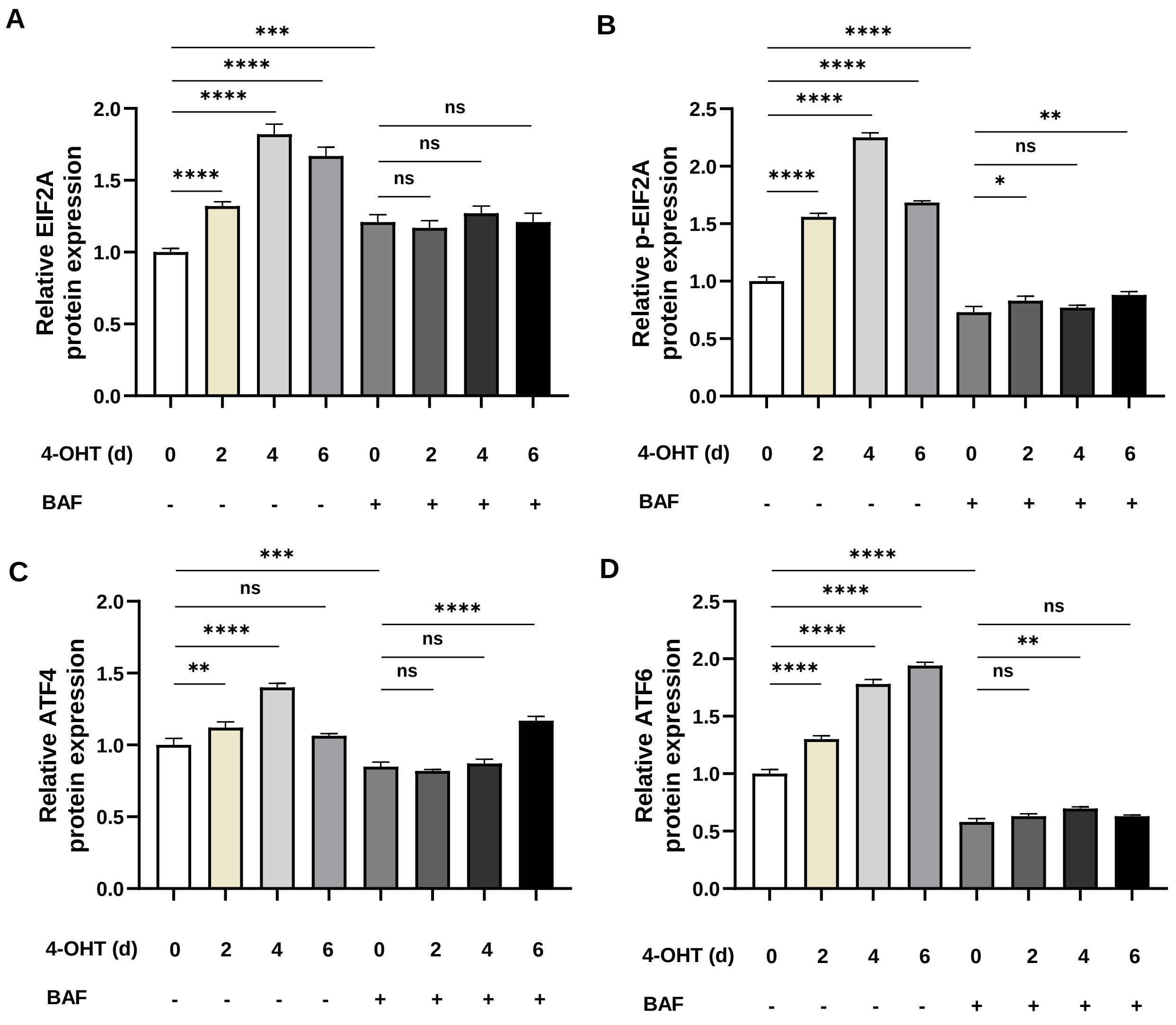

Supplementary Fig. 12. Quantification of immunoblot signals shown in Fig. 5G.

(A-D) Quantification of immunoblot signals shown in Fig. 5G. Protein levels were normalized to GAPDH, *n*=4 per group. Data are shown as mean ± SD, ***P* < 0.01, ****P* < 0.001, *****P* < 0.0001, *ns* = not significant.

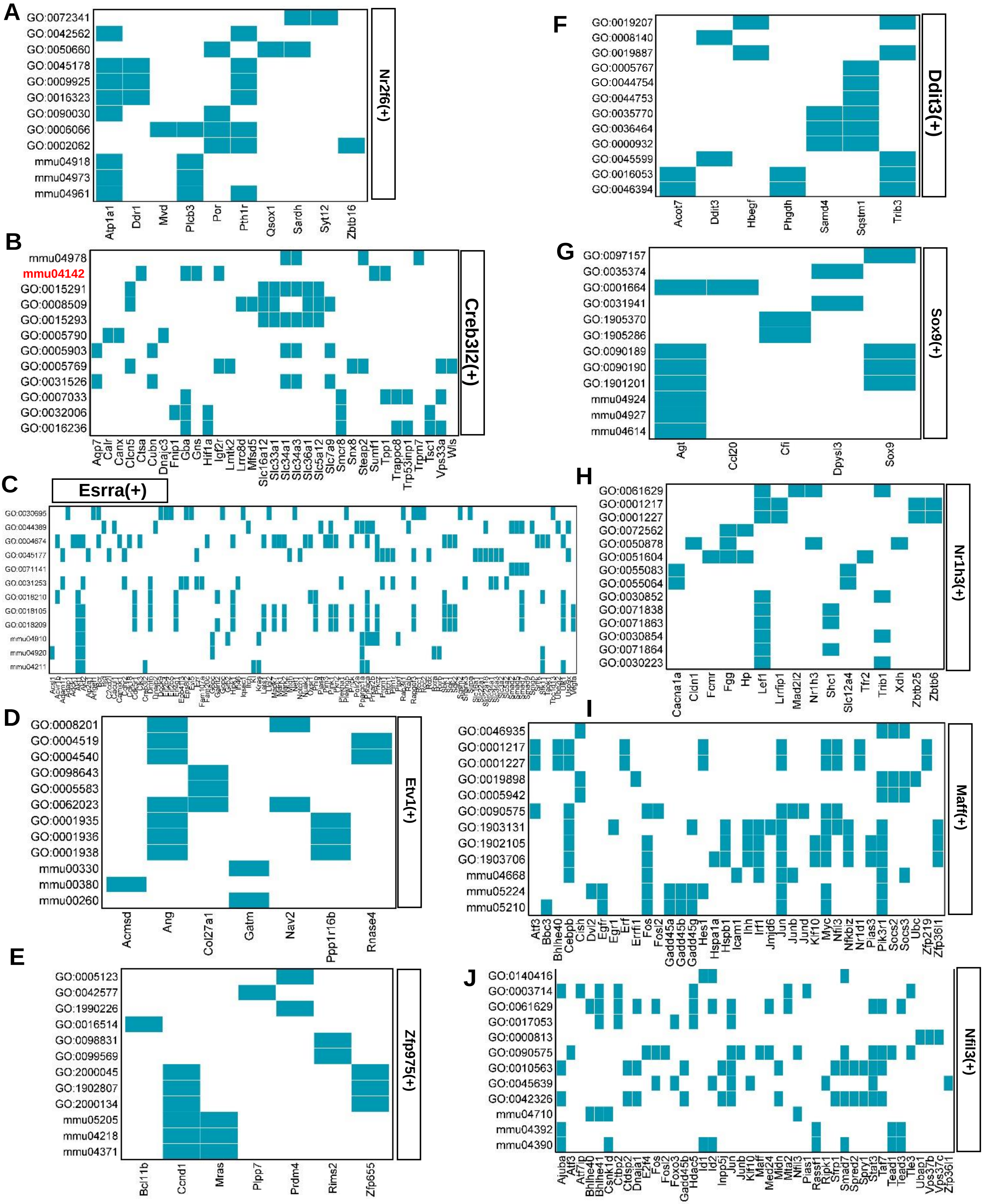

A
GO:0072341
GO:0042562
GO:0050660
GO:0045178
GO:0009925
GO:0016323
GO:0090030
GO:0006066
GO:0002062
mmu04918
mmu04973
mmu04961
Nr2f6(+)
Atp1a1
Ddr1
Mvd
Plcb3
Por
Pth1r
Qsox1
Sardh
Syt12
Zbtb16
B
mmu04978
mmu04142
GO:0015291
GO:0008509
GO:0015293
GO:0005790
GO:0005903
GO:0005769
GO:0031526
GO:0007033
GO:0032006
GO:0016236
Creb3l2(+)
Aqp7
Calr
Canx
Clcn5
Ctsa
Cubn
Dnajc3
Fnip1
Gba
Gns
Hif1a
Igf2r
Lmtk2
Lrrc8d
Mfsd5
Slc16a12
Slc33a1
Slc34a1
Slc34a3
Slc36a1
Slc5a12
Slc7a9
Smcr8
Snx8
Steap2
Sumf1
Tpp1
Trappc8
Trp53inp1
Trpm7
Tsc1
Vps33a
Wls
C
Esrra(+)
GO:0030695
GO:0044389
GO:0004674
GO:0045177
GO:0071141
GO:0031253
GO:0018210
GO:0018105
GO:0018209
mmu04910
mmu04920
mmu04211
D
GO:0008201
GO:0004519
GO:0004540
GO:0098643
GO:0005583
GO:0062023
GO:0001935
GO:0001936
GO:0001938
mmu00330
mmu00380
mmu00260
Etv1(+)
Acmsd
Ang
Col27a1
Gatm
Nav2
Ppp1r16b
Rnase4
E
GO:0005123
GO:0042577
GO:1990226
GO:0016514
GO:0098831
GO:0099569
GO:2000045
GO:1902807
GO:2000134
mmu05205
mmu04218
mmu04371
Zfp975(+)
Bcl11b
Ccnd1
Mras
Plpp7
Prdm4
Rims2
Zfp655
F
GO:0019207
GO:0008140
GO:0019887
GO:0005767
GO:0044754
GO:0044753
GO:0035770
GO:0036464
GO:0000932
GO:0045599
GO:0016053
GO:0046394
Ddit3(+)
Acot7
Ddit3
Hbegf
Phgdh
Samd4
Sqstm1
Trib3
G
GO:0097157
GO:0035374
GO:0001664
GO:0031941
GO:1905370
GO:1905286
GO:0090189
GO:0090190
GO:1901201
mmu04924
mmu04927
mmu04614
Sox9(+)
Agt
Ccl20
Cfi
Dpysl3
Sox9
H
GO:0061629
GO:0001217
GO:0001227
GO:0072562
GO:0050878
GO:0051604
GO:0055083
GO:0055064
GO:0030852
GO:0071838
GO:0071863
GO:0030854
GO:0071864
GO:0030223
Nr1h3(+)
Cacna1a
Cldn1
Fcmr
Fgg
Hp
Lef1
Lrrfip1
Mad2l2
Nr1h3
Shc1
Slc12a4
Tfr2
Trib1
Xdh
Zbtb25
Zbtb6
I
GO:0046935
GO:0001217
GO:0001227
GO:0019898
GO:0005942
GO:0090575
GO:1903131
GO:1902105
GO:1903706
mmu04668
mmu05224
mmu05210
Maff(+)
Atf3
Bbc3
Bhlhe40
Cebpb
Cish
Dvl2
Egfr
Egr1
Erf
Errfi1
Fos
Fosl2
Gadd45a
Gadd45b
Gadd45g
Hes1
Hspa1a
Hspb1
Icam1
Ihh
Irf1
Jmjd6
Jun
Junb
Jund
Klf10
Myc
Nfil3
Nfkbiz
Nr1d1
Pias3
Pik3r1
Socs2
Socs3
Ubc
Zfp219
Zfp36l1
J
GO:0140416
GO:0003714
GO:0061629
GO:0017053
GO:0000813
GO:0090575
GO:0010563
GO:0045639
GO:0042326
mmu04710
mmu04392
mmu04390
Nfil3(+)
Ajuba
Atf3
Atf7ip
Bhlhe40
Bhlhe41
Csnk1d
Ctbp2
Ctdsp2
Dnaja1
E2f4
Fos
Fosl2
Foxo3
Gadd45b
Hdac5
Id1
Id2
Inpp5j
Jun
Junb
Klf10
Maff
Med24
Midn
Mta2
Nfil3
Pias1
Rassf1
Ripk1
Sfrp1
Smad7
Spred2
Spry1
Stat3
Taf7
Tead1
Tead3
Tle3
Ubap1
Vps37b
Vps37c
Zfp36l1

Supplementary Fig. 13. Enrichment of GO and KEGG Pathways for transcription factors and their target genes in Cluster 1 and Cluster 3

(A-J) Gene Ontology (GO) and KEGG pathway enrichment analyses were performed on the transcription factors identified in Cluster 1 and Cluster 3 (*Nfkb1*, *Creb3l2*, *Esrra*, *Evl*, *Zbtb32*, *Ddit3*, *Maf*, *Nf1*, *Maff*, and *Jun*). The target genes of each transcription factor were analyzed for significant enrichment in various biological processes, molecular functions, cellular components, and KEGG pathways. The bars represent the degree of enrichment, with longer bars indicating stronger enrichment in the corresponding terms.

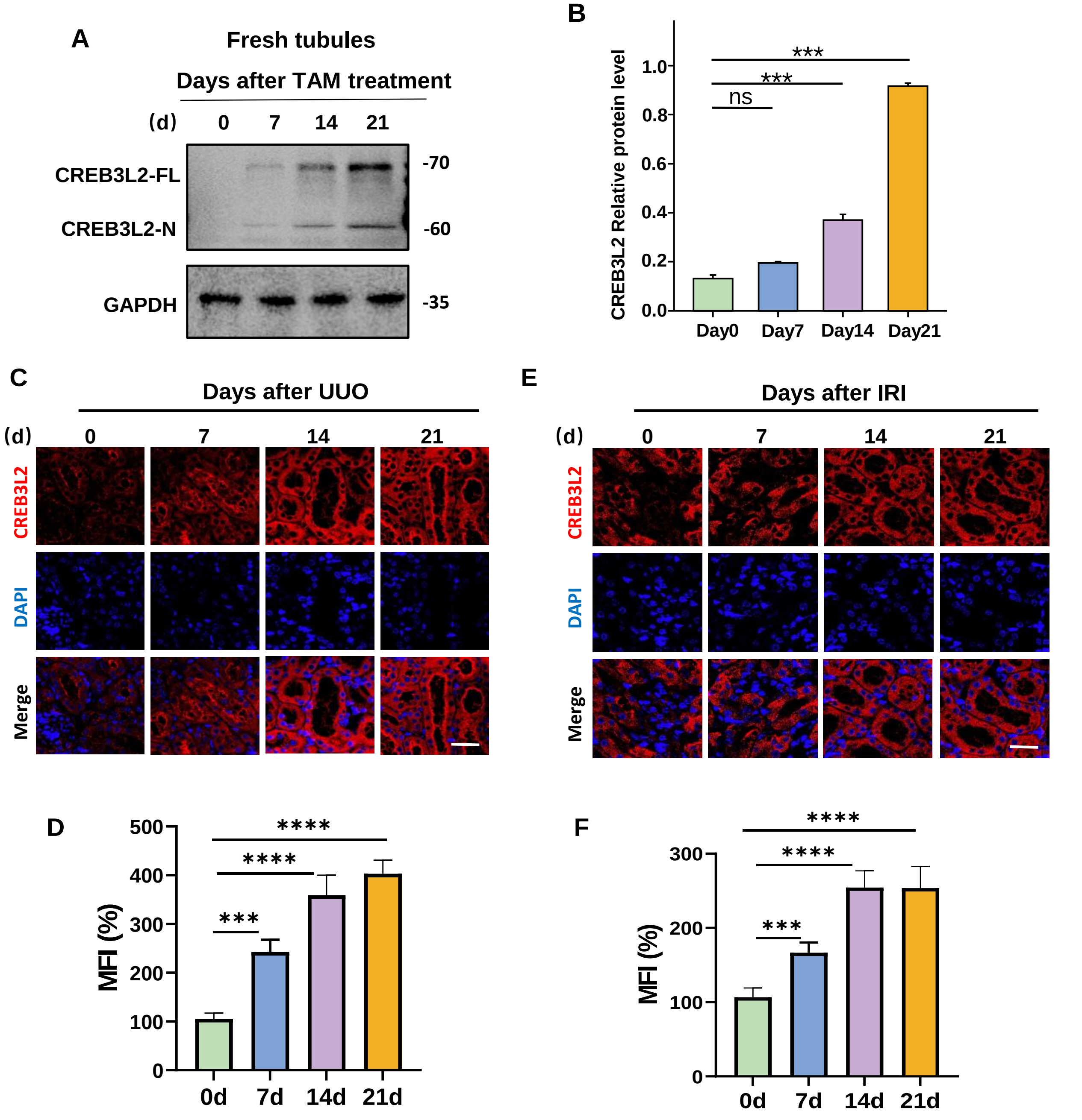
A
Fresh tubules
Days after TAM treatment
(d) 0 7 14 21
CREB3L2-FL
CREB3L2-N
GAPDH
-70
-60
-35
B
CREB3L2 Relative protein level
1.0
0.8
0.6
0.4
0.2
0.0
***
***
ns
Day0
Day7
Day14
Day21
C
Days after UUO
(d) 0 7 14 21
CREB3L2
DAPI
Merge
E
Days after IRI
(d) 0 7 14 21
CREB3L2
DAPI
Merge
D
MFI (%)
500
400
300
200
100
0
****
****
***
0d
7d
14d
21d
F
MFI (%)
300
200
100
0
****
****
***
0d
7d
14d
21d

Supplementary Fig. 14. CREB3L2 is progressively upregulated during renal injury in vivo.

(A) Representative immunoblot of freshly isolated renal tubules from *Pi4ka*$^{fl/fl}$ *Ksp*^{CreERT2} mice at the indicated days after TAM administration, showing both full-length CREB3L2 (CREB3L2-FL) and the cleaved N-terminal form (CREB3L2-N), consistent with CREB3L2 activation. n = 4 per group. GAPDH served as a loading control.

(B) Densitometric quantification of the immunoblot in panel A. CREB3L2 signals were normalized to GAPDH and expressed relative to Day 0, n=4 per group. Data are shown as mean ± SD, ***P<0.001, *ns*=not significant

(C, E) Representative immunofluorescence images showing CREB3L2 (red) with DAPI nuclear counterstaining (blue) at the indicated time points. (C) Kidneys collected at 0, 7, 14, and 21 days after UUO. (E) Kidneys collected at 0, 7, 14, and 21 days after IRI injury, n=6 per group. Scale bars=50μm.

(D, F). Quantification of CREB3L2 fluorescence intensity (MFI) corresponding to (C) and (E) at the indicated days after UUO and IRI, respectively, n = 4 per group. Data are expressed as mean ± SD. ***P < 0.001, ****P < 0.0001.

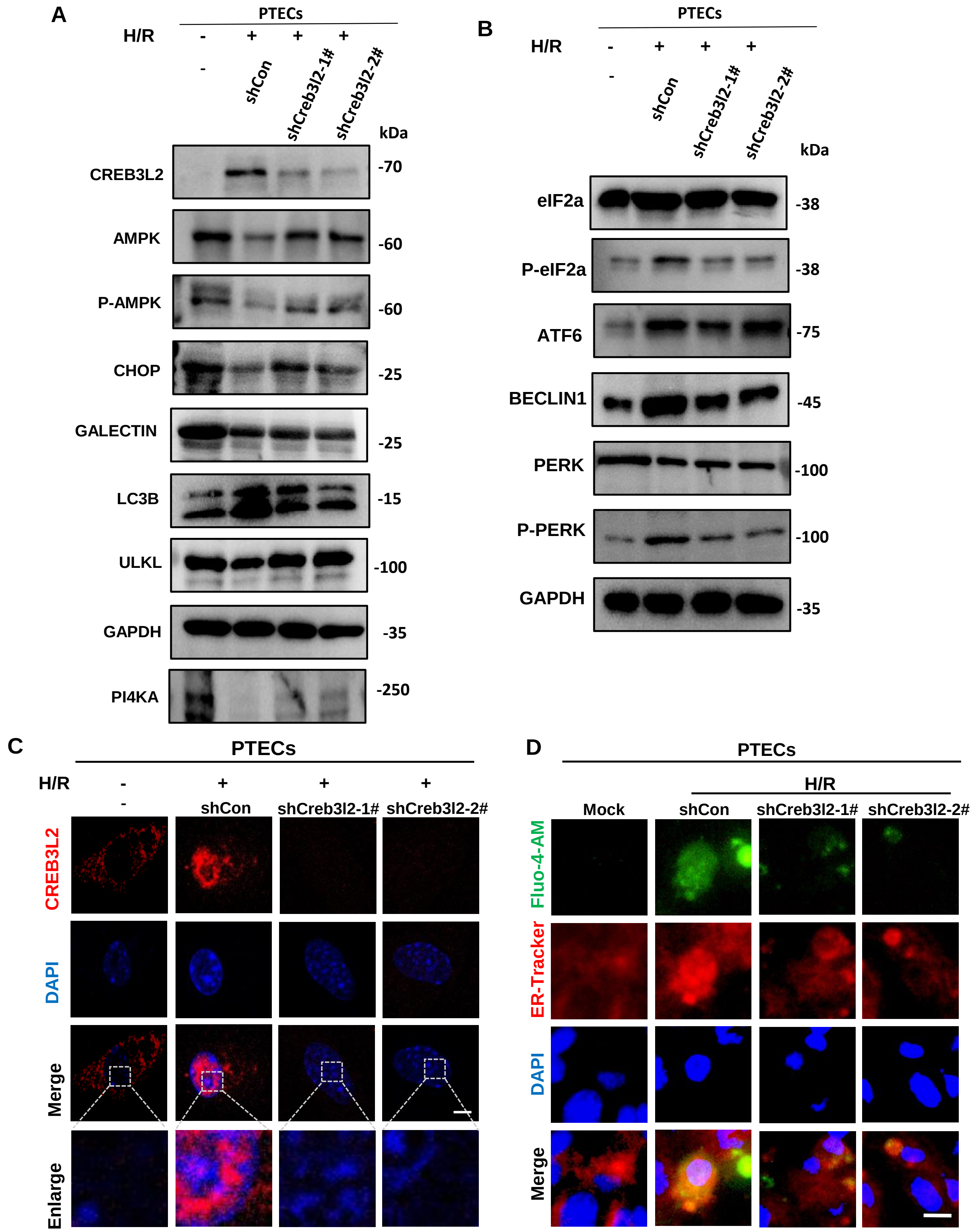
A
PTECs
H/R - + + +
- shCon shCreb3l2-1# shCreb3l2-2#
kDa
CREB3L2 -70
AMPK -60
P-AMPK -60
CHOP -25
GALECTIN -25
LC3B -15
ULKL -100
GAPDH -35
PI4KA -250
B
PTECs
H/R - + + +
- shCon shCreb3l2-1# shCreb3l2-2#
kDa
eIF2a -38
P-eIF2a -38
ATF6 -75
BECLIN1 -45
PERK -100
P-PERK -100
GAPDH -35
C
PTECs
H/R - + + +
- shCon shCreb3l2-1# shCreb3l2-2#
CREB3L2
DAPI
Merge
Enlarge
D
PTECs
H/R
Mock shCon shCreb3l2-1# shCreb3l2-2#
Fluo-4-AM
ER-Tracker
DAPI
Merge

Supplementary Fig. 15. CREB3L2 induction and ER stress–associated Ca^{2+} dysregulation in PTECs after hypoxia/reoxygenation are attenuated by Creb3l2 knockdown.

(A–B) PTECs were subjected to hypoxia/reoxygenation (H/R) and transduced with control shRNA or two independent Creb3l2 shRNAs. Immunoblot analyses showing CREB3L2, PI4KA, and representative markers of ER stress signaling, lysosomal damage–associated readouts, and autophagy-related proteins, n=4 per group. GAPDH was used as a loading control.

(C) Representative immunofluorescence images of PTECs showing CREB3L2 (red) and DAPI nuclear staining (blue) under basal conditions or after hypoxia/reoxygenation (H/R). In control shRNA–transduced cells, H/R induces a nuclear-enriched CREB3L2 signal consistent with activation, whereas transduction with two independent Creb3l2 shRNAs markedly reduces both overall CREB3L2 staining and the nuclear signal. n=6 per group. Scale bars=10μm .

(D) Representative fluorescence images of PTECs after hypoxia/reoxygenation. Intracellular Ca^{2+} was assessed using Fluo-4 AM (green) and the endoplasmic reticulum was visualized with ER-Tracker (red); nuclei were counterstained with DAPI (blue). Mock and shCon denote untransduced and control shRNA conditions, respectively, n=6 per group. Scale bars=50μm .

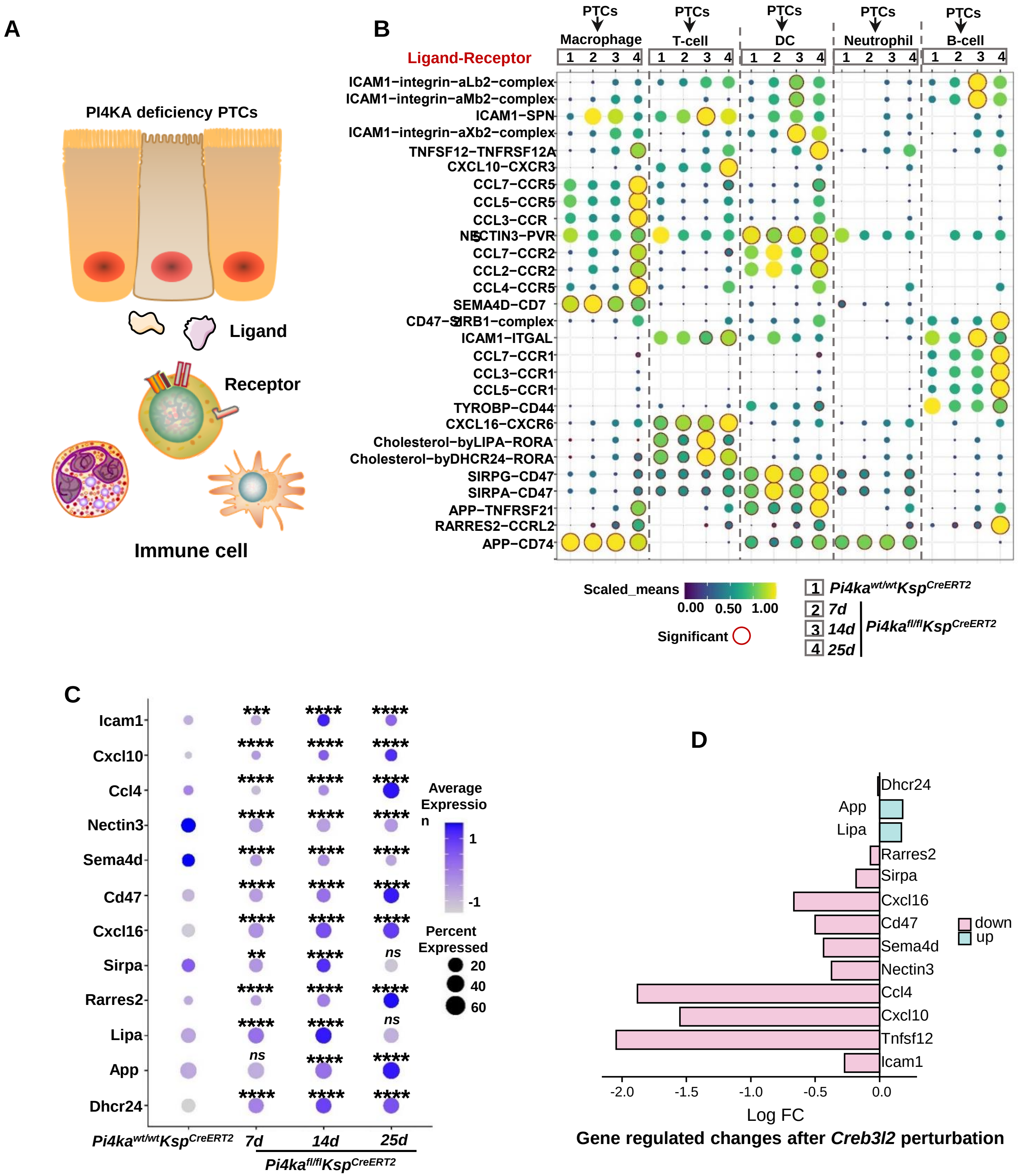
A
PI4KA deficiency PTCs
Ligand
Receptor
Immune cell
B
Ligand-Receptor
PTCs
Macrophage
T-cell
DC
Neutrophil
B-cell
1 2 3 4
ICAM1−integrin−aLb2−complex
ICAM1−integrin−aMb2−complex
ICAM1−SPN
ICAM1−integrin−aXb2−complex
TNFSF12−TNFRSF12A
CXCL10−CXCR3
CCL7−CCR5
CCL5−CCR5
CCL3−CCR
NECTIN3−PVR
CCL7−CCR2
CCL2−CCR2
CCL4−CCR5
SEMA4D−CD72
CD47−SIRB1−complex
ICAM1−ITGAL
CCL7−CCR1
CCL3−CCR1
CCL5−CCR1
TYROBP−CD44
CXCL16−CXCR6
Cholesterol−byLIPA−RORA
Cholesterol−byDHCR24−RORA
SIRPG−CD47
SIRPA−CD47
APP−TNFRSF21
RARRES2−CCRL2
APP−CD74
Scaled_means
0.00 0.50 1.00
Significant
1 Pi4ka wt/wt Ksp CreERT2
2 7d
3 14d
4 25d
Pi4ka fl/fl Ksp CreERT2
C
Icam1
Cxcl10
Ccl4
Nectin3
Sema4d
Cd47
Cxcl16
Sirpa
Rarres2
Lipa
App
Dhcr24
***
****
**
ns
Average Expression
1
-1
Percent Expressed
20
40
60
Pi4ka wt/wt Ksp CreERT2
7d
14d
25d
Pi4ka fl/fl Ksp CreERT2
D
Dhcr24
App
Lipa
Rarres2
Sirpa
Cxcl16
Cd47
Sema4d
Nectin3
Ccl4
Cxcl10
Tnfsf12
Icam1
down
up
-2.0
-1.5
-1.0
-0.5
0.0
Log FC
Gene regulated changes after Creb3l2 perturbation

.**Supplementary Fig. 16. Creb3l2 Is Required for the Pro-Inflammatory Response in *Pi4ka*-Deficient PTCs.**

(A) A sketch demonstrating the *Pi4ka*-Deficient PTCs secrete the ligands to promote the recruitment of immune cells.

(B) CellPhoneDB-based ligand–receptor interaction analysis was performed using PTCs as ligand-expressing cells and immune cell populations (macrophages, T cells, dendritic cells, neutrophils, and B cells) as receptor-expressing cells. The analysis revealed multiple significantly enriched ligand–receptor pairs.

(C) Dot plot analysis of PTCs demonstrated that the majority of ligand-related genes identified by the interaction analysis (e.g., *Icam1*, *Cxcl10*, *Ccl4*, *Nectin3*, *Sema4d*, *Cd47*, *Cxcl16*, *Sirpa*, *Rarres2*, *Lipa*, *App*, *Dhcr24*) were significantly upregulated following *Pi4ka* deletion. $^{**}P<0.01$, $^{***}P<0.001$, $^{****}P<0.0001$, *ns*= not significant.

(D) Differential expression analysis of *Creb3l2*-deficient PTCs showed that most of these ligand-associated genes were markedly downregulated after *Creb3l2* knockdown.

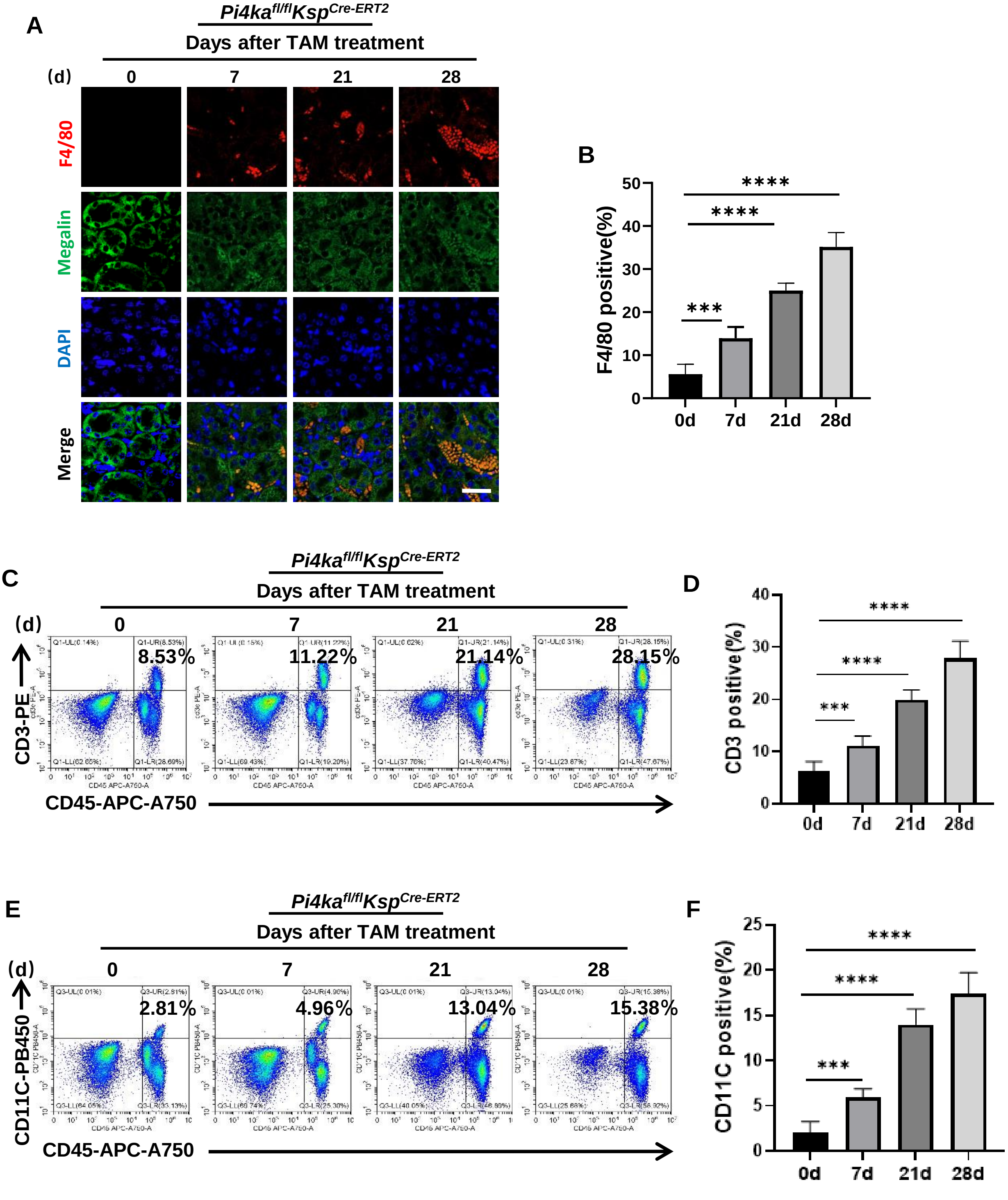
A
Pi4ka fl/fl Ksp Cre-ERT2
Days after TAM treatment
(d)
0
7
21
28
F4/80
Megalin
DAPI
Merge
B
F4/80 positive(%)
0
10
20
30
40
50
0d
7d
21d
28d
***
****
****
C
Pi4ka fl/fl Ksp Cre-ERT2
Days after TAM treatment
(d)
0
7
21
28
8.53%
11.22%
21.14%
28.15%
CD3-PE
CD45-APC-A750
D
CD3 positive(%)
0
10
20
30
40
0d
7d
21d
28d
***
****
****
E
Pi4ka fl/fl Ksp Cre-ERT2
Days after TAM treatment
(d)
0
7
21
28
2.81%
4.96%
13.04%
15.38%
CD11C-PB450
CD45-APC-A750
F
CD11C positive(%)
0
5
10
15
20
25
0d
7d
21d
28d
***
****
****

Supplementary Fig. 17. Spatial localization and temporal dynamics of immune cell infiltration following TAM-induced Pi4ka deletion.

(A) Representative immunofluorescence images of kidney sections from $Pi4ka^{fl/fl}\ Ksp^{Cre\text{-}ERT2}$ mice at the indicated time points after TAM treatment (day 0, 7, 21, and 28). Macrophages (F4/80, Red) and proximal tubular cells (Megalin, green) were stained; nuclei were counterstained with DAPI (blue). Merged images show the spatial distribution of infiltrating immune cells relative to $Megalin^{+}$ tubules over time. Scale bar = 50 μm. $n = 6$ per group.

(B) Quantification of $F4/80^{+}$ signals（positive, %）at indicated time points after TAM treatment, n=6 per group. Data are shown as mean ± SD, ***$P < 0.001$， ****$P < 0.0001$.

(C–F) Flow cytometry analysis of kidney immune cells at the indicated time points after TAM treatment. Representative plots show the frequency of $CD3^{+}$ T cells (C) and $CD11C^{+}$ dendritic cells (E) among $CD45^{+}$ immune cells. Bar graphs summarize the percentages of $CD3^{+}$ (D) and $CD11C^{+}$ (F) cells (as % of $CD45^{+}$ cells). Data are shown as mean ± SD. $n = 6$ per group. ***$P < 0.001$, ****$P < 0.0001$.

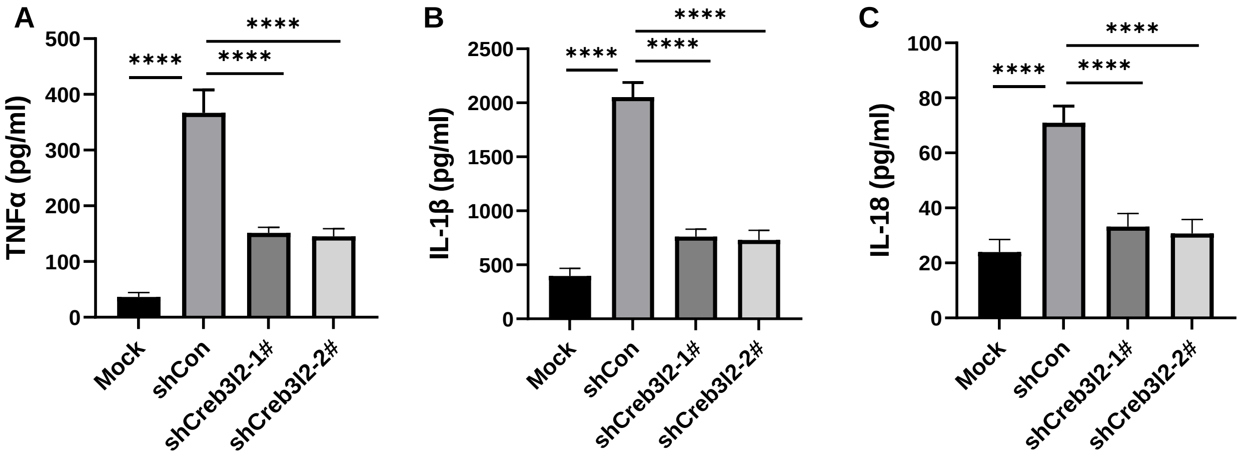

Supplementary Fig. 18. *Creb3l2* knockdown suppresses inflammatory cytokine release in PTECs after hypoxia/reoxygenation.

(A–C) Concentrations of TNFα, IL-1β, and IL-18 in cell culture supernatants measured under the indicated conditions, n=6 per group. Data are presented as mean ± SD, ****$P < 0.0001$.

Reference

1. Chen Z, Li Y, Yuan Y, Lai K, Ye K, Lin Y, Lan R, Chen H, Xu Y. Single-cell sequencing reveals homogeneity and heterogeneity of the cytopathological mechanisms in different etiology-induced AKI. Cell Death Dis 2023, 14(5): 318.

2. Lai K, Chen Z, Lin S, Ye K, Yuan Y, Li G, Song Y, Ma H, Mak TW, Xu Y. The IDH1-R132H mutation aggravates cisplatin-induced acute kidney injury by promoting ferroptosis through disrupting NDUFA1 and FSP1 interaction. Cell Death Differ 2024.

3. Chen C, Xie J, Chen Z, Ye K, Wu C, Dai X, Yuan Y, Lin Y, Wang Y, Chen H, Wu J, Ma H, Xu Y. Role of Z-DNA Binding Protein 1 Sensing Mitochondrial Z-DNA and Triggering Necroptosis in Oxalate-Induced Acute Kidney Injury. J Am Soc Nephrol 2025, 36(3): 361-377.

4. Wang Y, Li Y, Chen Z, Yuan Y, Su Q, Ye K, Chen C, Li G, Song Y, Chen H, Xu Y. GSDMD-dependent neutrophil extracellular traps promote macrophage-to-myofibroblast transition and renal fibrosis in obstructive nephropathy. Cell Death Dis 2022, 13(8): 693.

5. Lai K, Wang J, Lin S, Chen Z, Lin G, Ye K, Yuan Y, Lin Y, Zhong CQ, Wu J, Ma H, Xu Y. Sensing of mitochondrial DNA by ZBP1 promotes RIPK3-mediated necroptosis and ferroptosis in response to diquat poisoning. Cell Death Differ 2024, 31(5): 635-650.

6. Ye K, Lan R, Chen Z, Lai K, Song Y, Li G, Ma H, Chen H, Xu Y. Roles of ACSL4/GPX4 and FSP1 in oxalate-induced acute kidney injury. Cell Death Discov 2025, 11(1): 279.

7. Chen Z, Lin G, Ye K, Wang J, Tang M, Lai K, Yuan Y, Lin S, Dai X, Chen H, Ma H, Zhou J, Xu Y. Single-cell analysis of diquat-induced oxidative stress and its impact on organ-specific toxicity. Ecotoxicol Environ Saf 2025, 297: 118246.

8. Troulé K, Petryszak R, Cakir B, Cranley J, Harasty A, Prete M, Tuong ZK, Teichmann SA, Garcia-Alonso L, Vento-Tormo R. CellPhoneDB v5: inferring cell-cell communication from single-cell multiomics data. Nat Protoc 2025.

9. Osorio D, Zhong Y, Li G, Xu Q, Yang Y, Tian Y, Chapkin RS, Huang JZ, Cai JJ. scTenifoldKnk: An efficient virtual knockout tool for gene function predictions via single-cell gene regulatory network perturbation. Patterns (N Y) 2022, 3(3): 100434.
