# Supplementary material for: Pi4ka downregulation triggers Creb3l2-dependent lysosomal dysfunction to promote maladaptive tubular remodeling and immune activation in acute kidney injury
Source: Cell Death Dis. 2026 Apr 27;17(1):557. doi: 10.1038/s41419-026-08794-y (PMC13250169; doi:10.1038/s41419-026-08794-y)

Figure5G

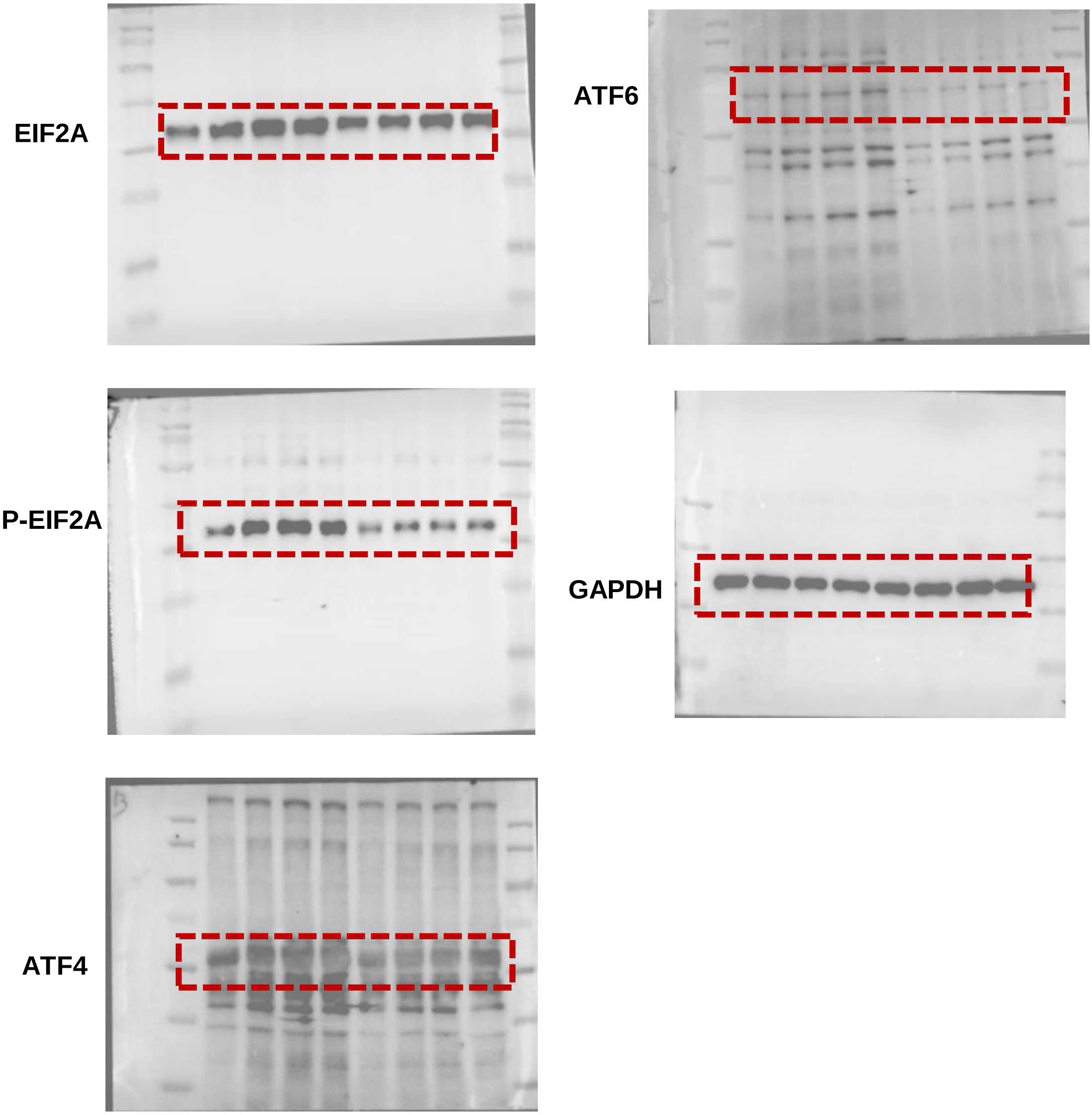

Figure7B

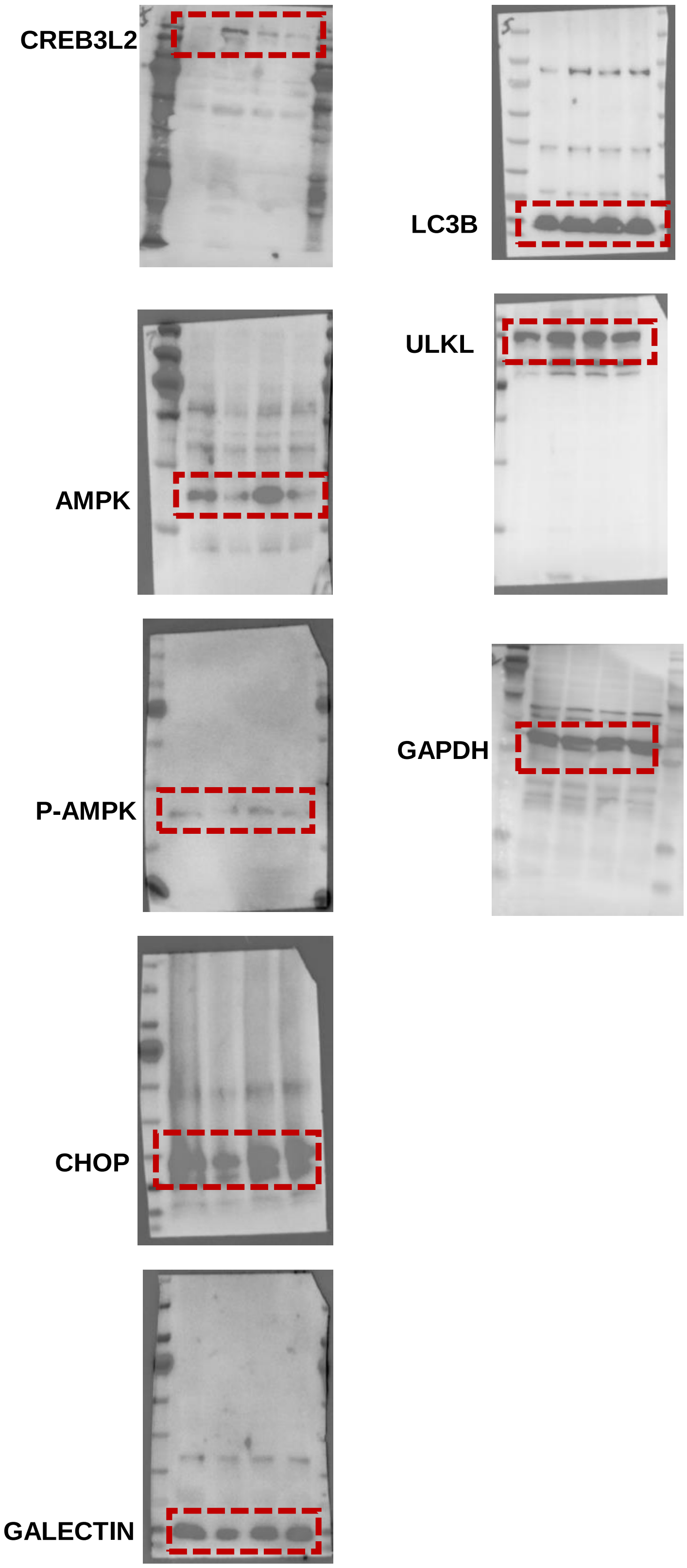

Figure7C

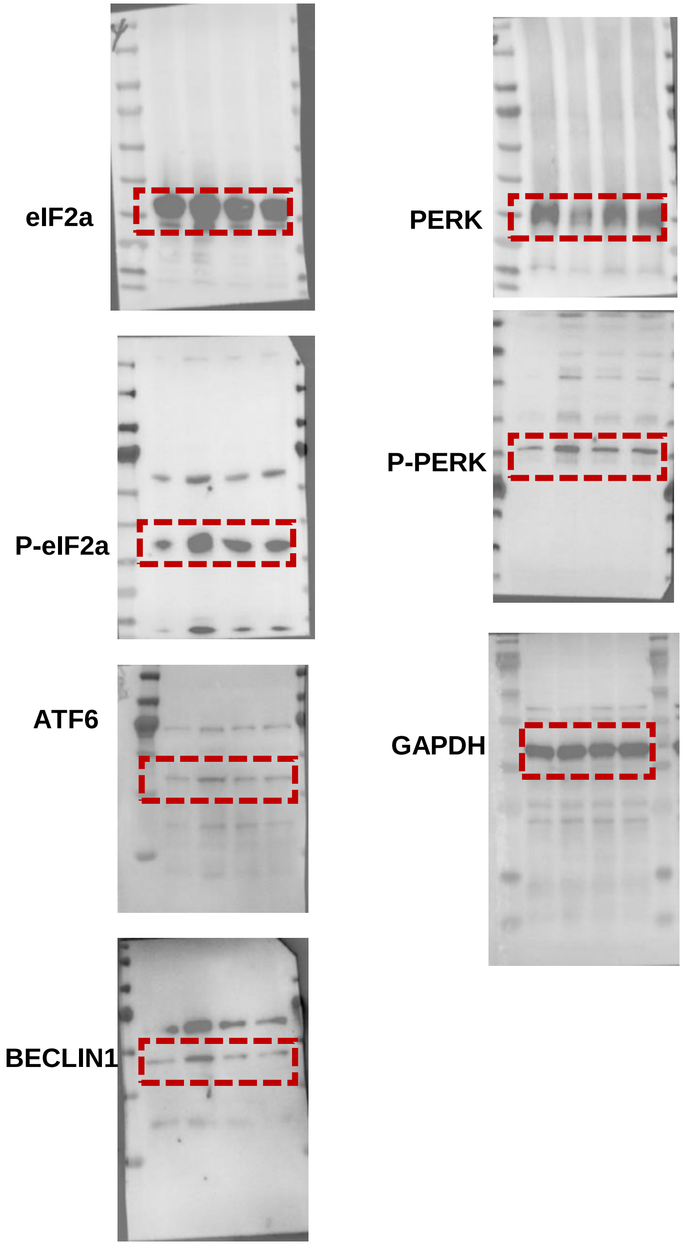

Supplementary Fig. 2A

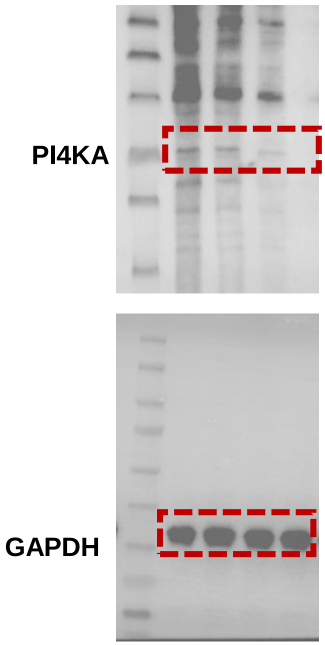

Supplementary Fig. 10A

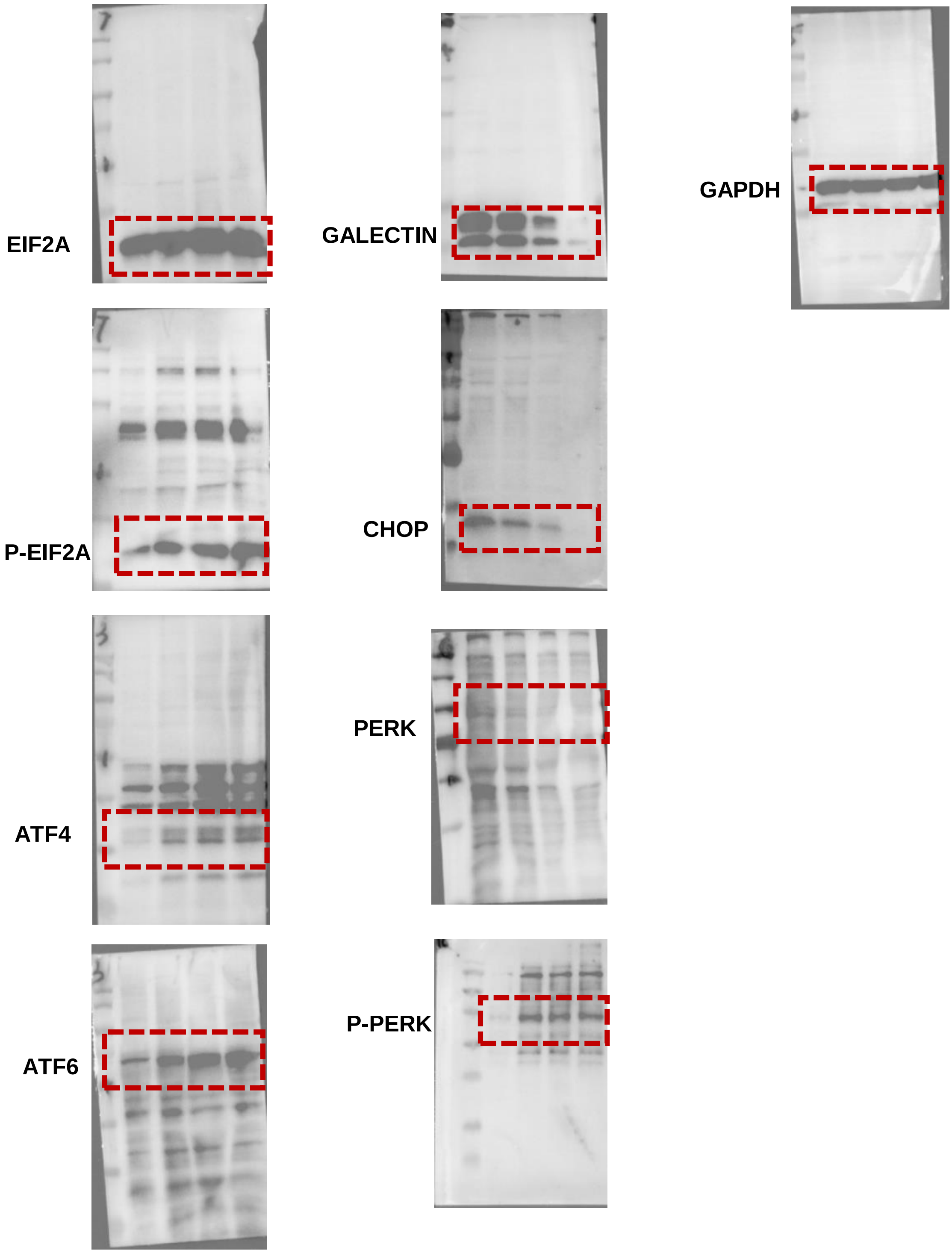

Supplementary Fig. 10B

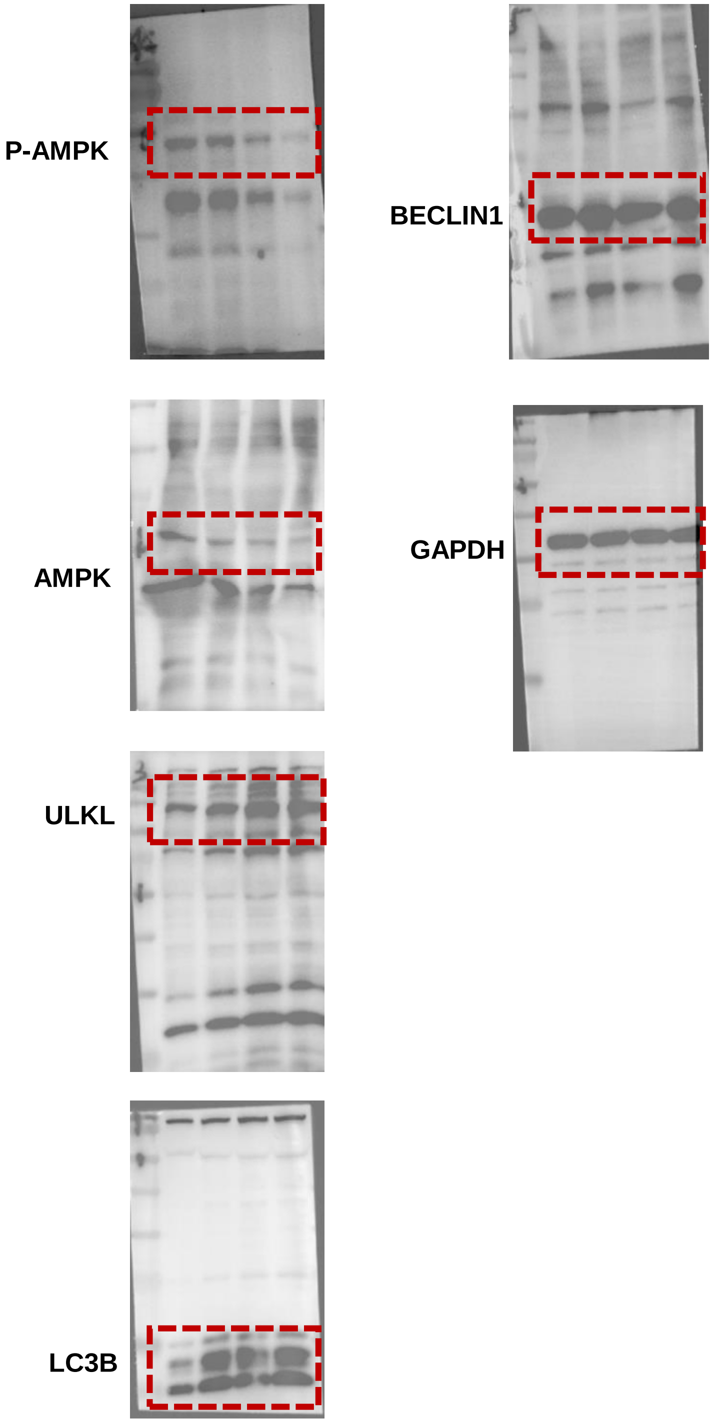

Supplementary Fig. 10C

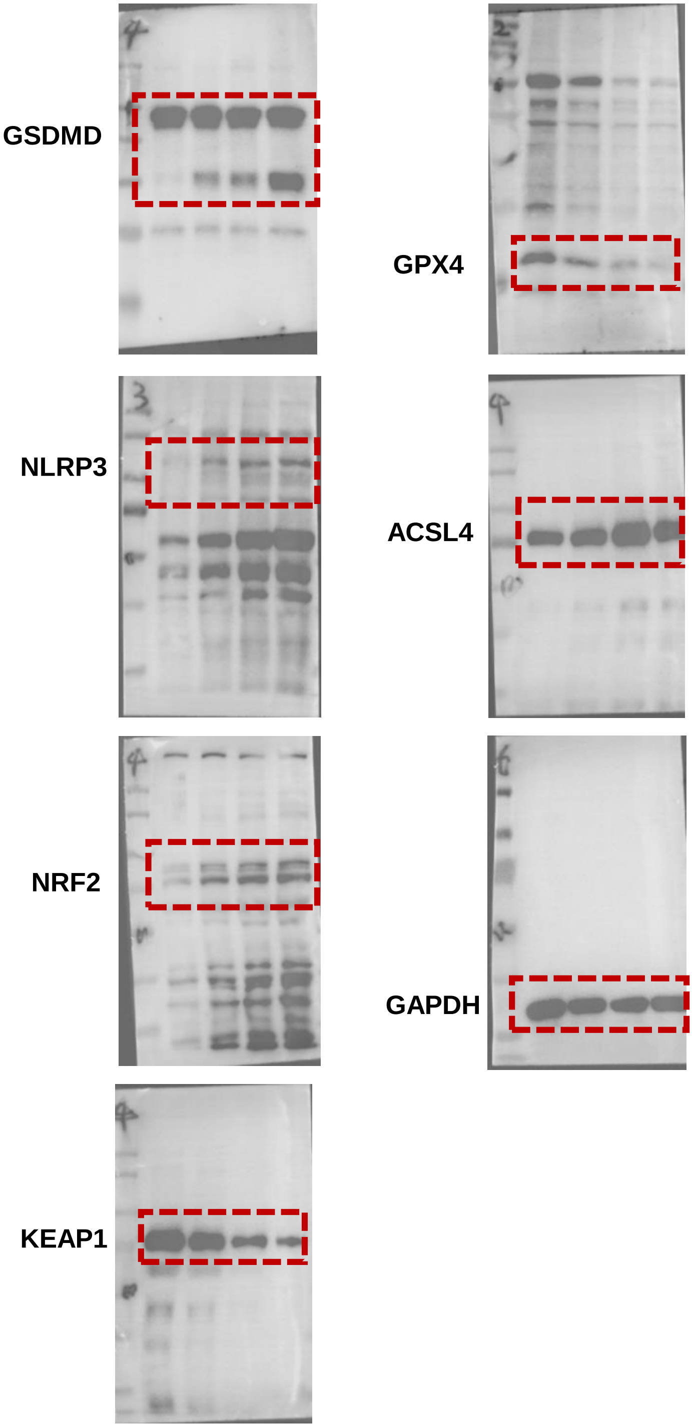

Supplementary Fig. 10D

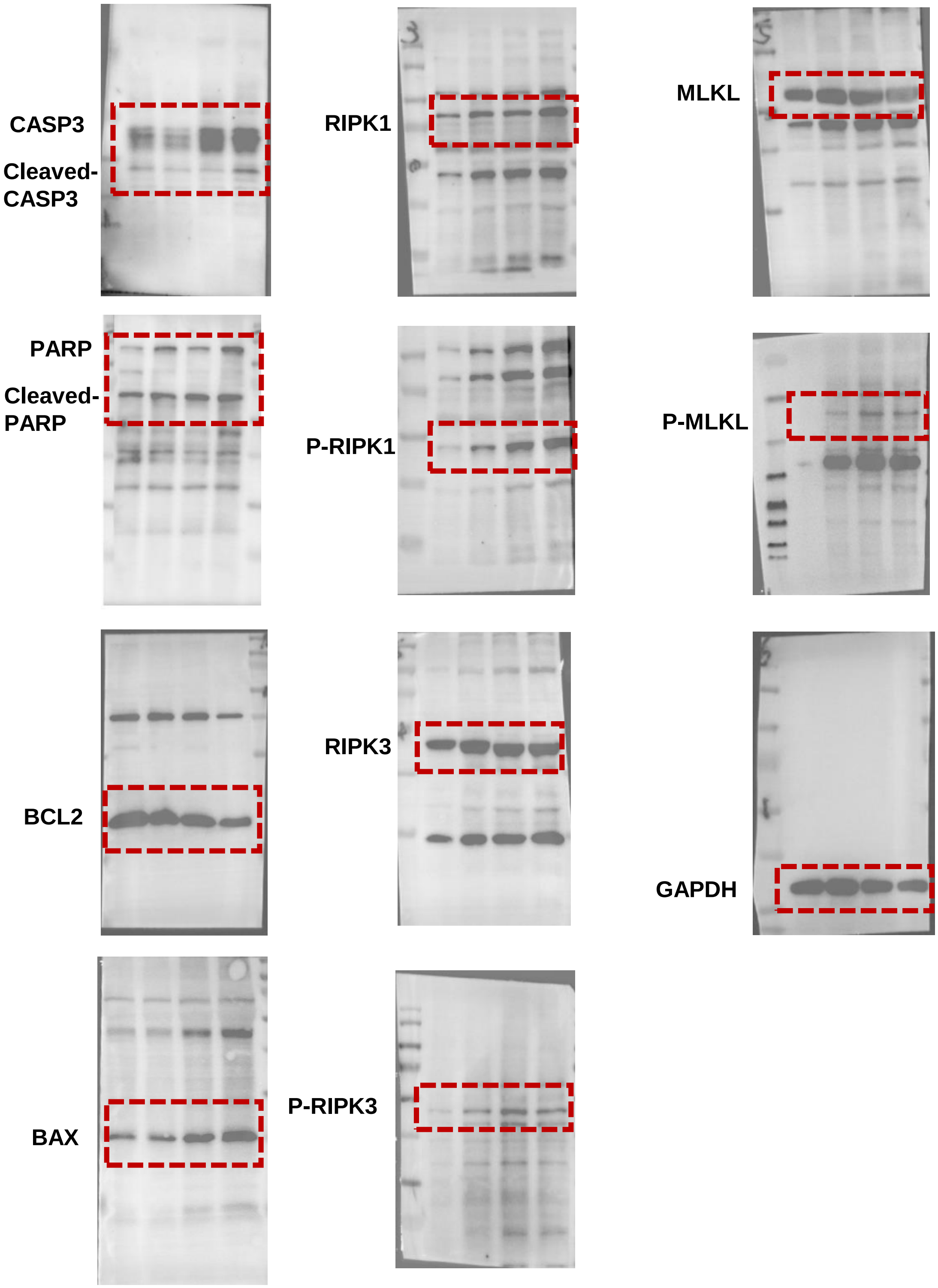

Supplementary Fig. 14A

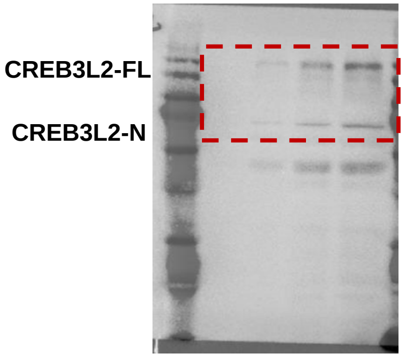

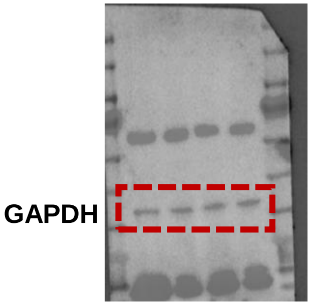

Supplementary Fig. 15A

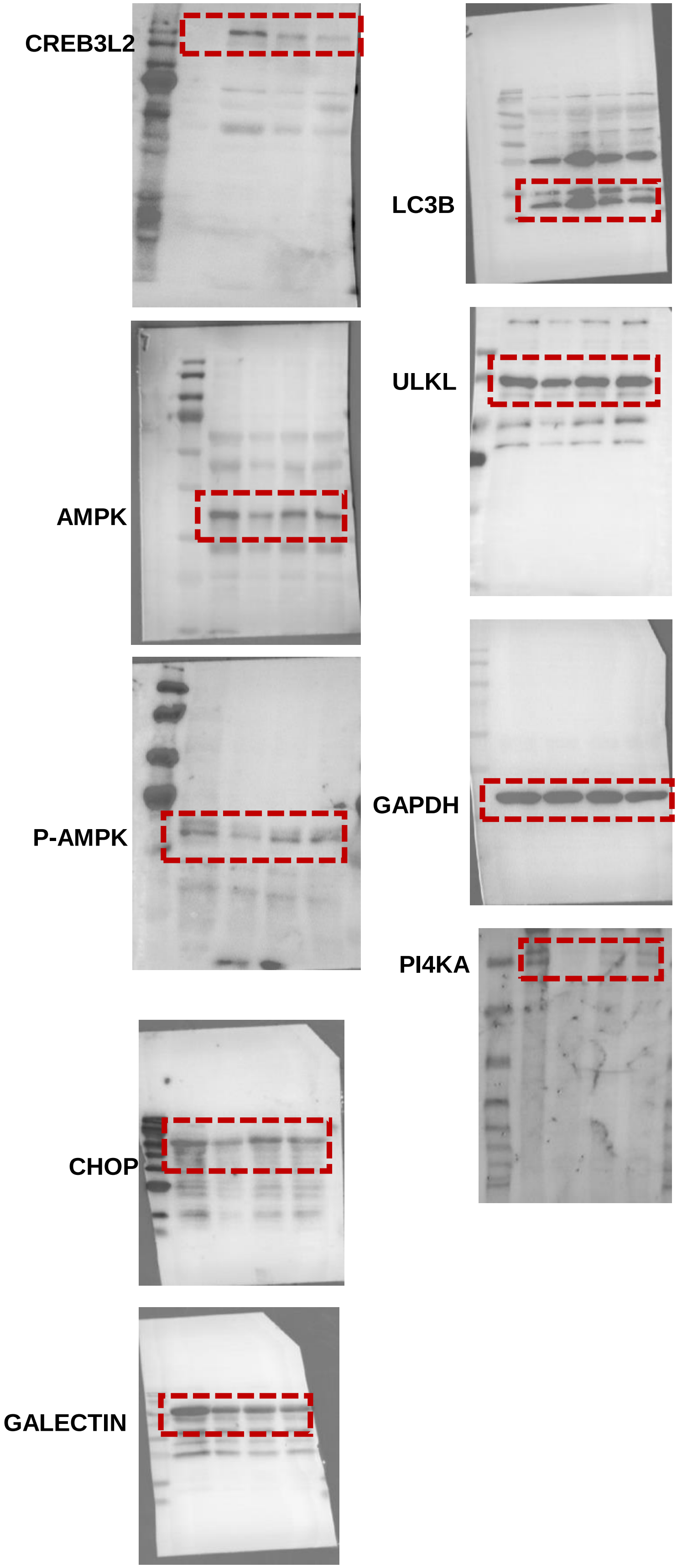

Supplementary Fig. 15B

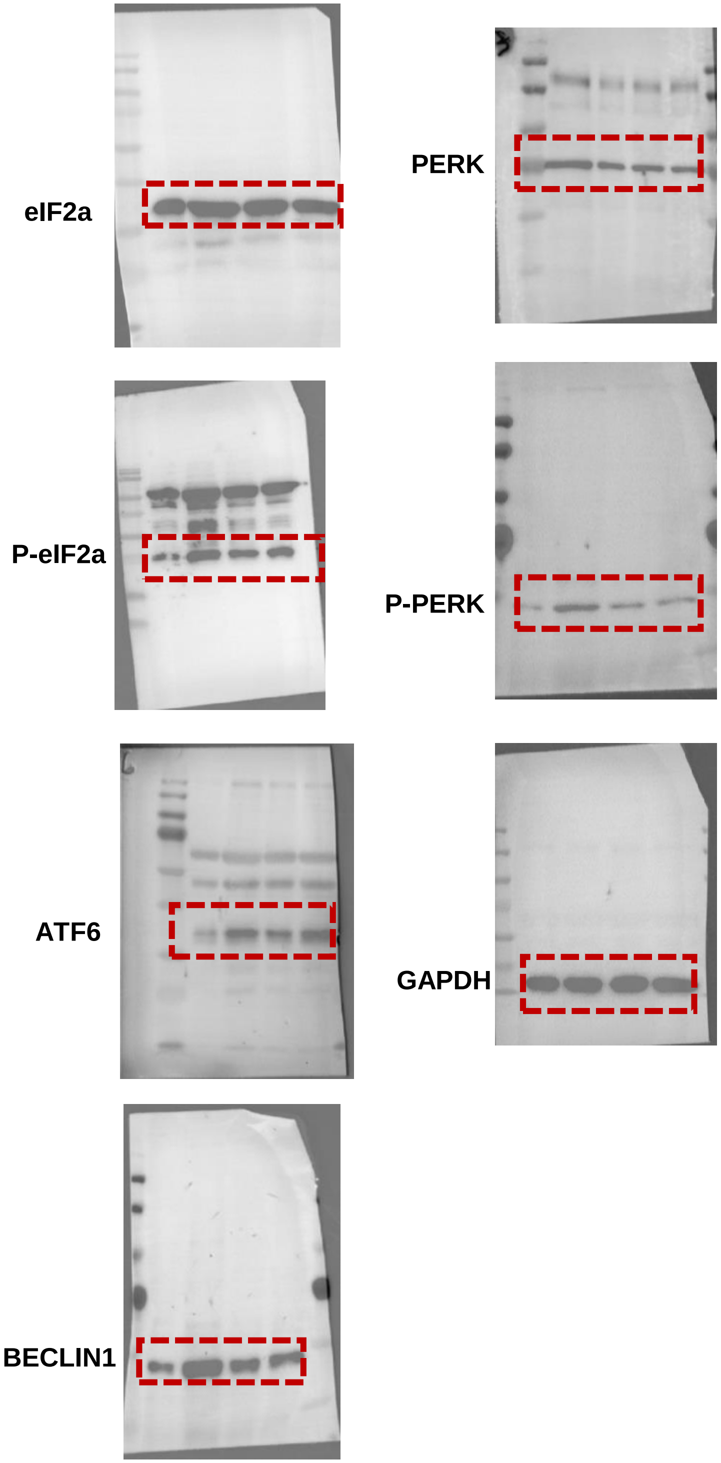

Supplement: Supplementary file 5 — Original WB images [file 41419_2026_8794_MOESM5_ESM.pdf]
